# 1*H*-1,2,3-triazolyl-1,6-naphthyridin-7(6*H*)-ones as Potential Fluorescent Nucleoside Analogues: Synthesis and Optical Properties

**DOI:** 10.3390/molecules29030687

**Published:** 2024-02-01

**Authors:** Anissa Beghennou, Océane Rondot, Vincent Corcé, Candice Botuha

**Affiliations:** Institut Parisien de Chimie Moléculaire, CNRS UMR 9232, Sorbonne Université, F-75252 Paris, France; anissa.beghennou@gmail.com (A.B.); oceane.rondot@sorbonne-universite.fr (O.R.)

**Keywords:** nucleoside, 1,2,3 triazole, CuAAC reaction, fluorescence, solvatochromism

## Abstract

In this article, we present the synthesis and the optical properties of three original molecules as potential fluorescent ribonucleoside analogues incorporating a 1,6-naphthyridin-7(6*H*)-one scaffold as a fluorescent nucleobase and a 1,2,3-triazole as a linkage. The nucleosides were prepared via a Cu alkyne-azide cycloaddition (CuAAC) reaction between a ribofuranosyl azide and a 4-ethynylpyridine partner. Construction of substituted 1,6-naphthyridin-7(6*H*)-ones was achieved through two additional steps. Optical property studies were investigated on nucleoside analogues. Powerful fluorescence properties have been evidenced with a remarkable change of emissivity depending on the polarity of the solvent, making these molecules suitable as a new class of artificial fluorescent nucleosides for investigating enzyme binding sites as well as probing nucleic acids. In addition, we are convinced that such analogues could be of great interest in the search for new antiviral or antitumoral drugs based on nucleosides.

## 1. Introduction

Nucleic acid structure and dynamics are of fundamental importance in understanding biological processes in cells [1,2,3]. In this context, nucleoside derivatives and analogues have been designed to interfere with cell metabolism and are being developed as antiviral, anticancer, and antibacterial agents [4,5,6] or as tools for several purposes, such as investigating enzyme binding sites, protein interactions, DNA features, and perturbations [7,8].

DNA-based fluorescent structures capable of labeling nucleic acids are powerful tools to investigate DNA interactions and have been used in a variety of applications in chemical biology [9,10,11,12]. 

In this context, numerous environmentally sensitive unnatural fluorescent nucleosides have been developed to fluorescently label nucleic acids via binding, intercalation, or covalent bonds [13,14]. The design of fluorescent nucleosides is a great challenge and has stimulated much research in various fields of photophysics, synthetic chemistry, and computational studies. The main fluorescence properties should include a change in fluorescence intensity or a shift in the emission maximum when interacting with the DNA environment while maintaining the other key optical properties, such as large Stokes Shift, high brightness, and high quantum yield.

**Figure 1:**
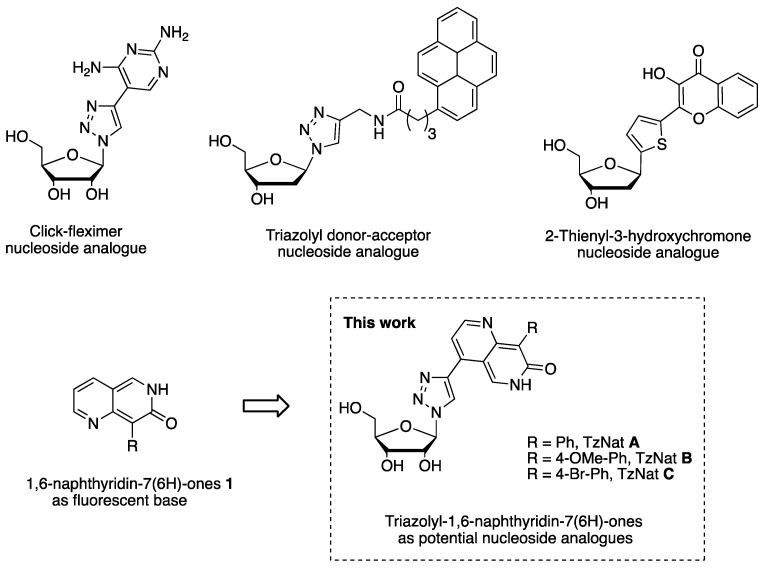
Selection of unnatural fluorescent nucleoside analogues reported in the literature [25,29,30,41] and the design of 1,2,3-triazolyl-1,6-naphthyridin-7(6*H*)-ones nucleoside analogues TzNat **A** to **C** presented in this work.

In this field, unnatural fluorescent nucleosides possessing heterocyclic nucleobases are widely represented. EthenoA [15], Coumarin nucleobase analogues [16], Nile red, imidazophenanthrene, and other types of heterocyclic nucleobases have been developed [17,18,19,20]. In particular, 1,2,3-triazolyl nucleoside analogues, which consist in connecting the appropriate aromatic ring or purine residue to a 1,2,3-triazole moiety, have found a growing interest due to their powerful antiviral activities [21,22,23]. 4-substituted-1,2,3 triazolo nucleotide analogues are also well known for antitumoral activities as inhibitors of human ecto-5′-nucleosidase CD73, a cell-surface protein associated with adenosine metabolism that promotes tumor progression [24]. Interestingly, a nucleoside analogue incorporating a diaminopyrimidine linked to a 1,2,3-triazole [25], also called click fleximer [26,27], was found to be luminescent and a promising tool to investigate enzyme binding sites and to characterize protein and nucleic acid interactions (Figure 1). Indeed, unlike the classic bicyclic fused system of the parent purine nucleobase, the two heterocyclic components of the click fleximers are attached by a C–C bond that permits conformational mobility. As a consequence, the relative flexibility of nucleobase enables it to adapt rapidly to the spatial requirement of an enzyme binding site. The design of flexible bioprobes will clearly provide a better understanding of the conformational effects of enzymes, and elucidate the structure of ligand binding sites in biologically important enzyme systems [28]. Other synthetic fleximer derivatives whose heterocyclic bases contain one or more than one planar ring capable of interacting with their environment, via π stacking and/or H-bonding, have been discovered. For example, a fluorescent triazolyl containing a pyrene ring has shown interesting fluorescence properties and capacity to interact with BSA via hydrophobic and electrostatic bindings [29] (Figure 1). Interestingly, an environment-sensitive fluorescent nucleoside analogue bearing a thienyl ring linked to a 3-hydroxychromone (3HCnt) as ESIPT dye has been successfully incorporated into a DNA sequence with minimal perturbation. In addition, the authors demonstrated that 3HCnt can monitor local conformation changes of oligonucleotides upon interaction with HIV-1 nucleocapsid protein [30].

Naphthyridines [31] are important scaffolds used for biological applications [32,33]. These structures are also known as fluorescent probes [34] or as luminescent materials [35]. 1,8-Naphthyridine C-nucleoside and their base-pairing properties have been reported [36]. Among these structures, naphthyridinones are also well known for their biological properties [37]. Only the 1,8-naphthyridinones have been particularly studied as nucleobase analogues. 1,8-naphthyridin-2(1*H*)-ones were described as efficient bicyclic and tricyclic fluorescent-based analogues of thymines [38] and were further incorporated into DNA [39,40]. The naphthyridinones thus appear as promising scaffolds to be developed as new fluorescent nucleosides.

**Scheme 1:**
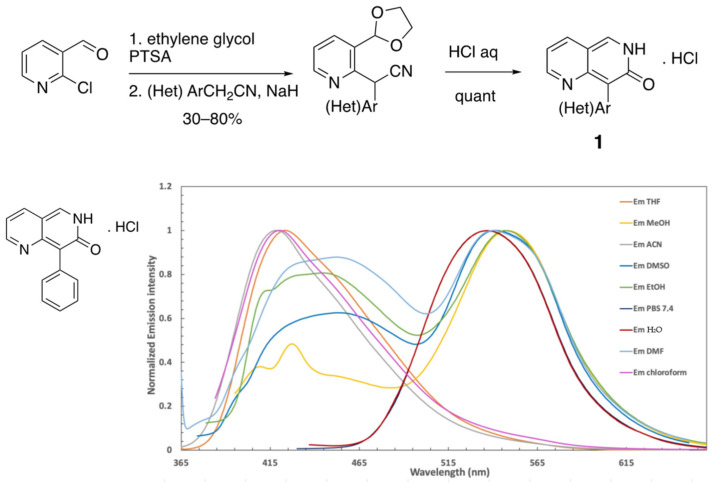
Our reported 8-substituted 1,6-naphthyridin-7(6*H*)-ones **1** for Ar = Ph and their emission spectra in various solvents, adapted with permission from Ref. [41]. 2023, Royal Chemical Society (RCS).

We have recently reported the short synthesis of 1,6-naphthyridin-7(6*H*)-ones **1**, a fluorescent heteroaromatic scaffold with adequate photophysical properties to be incorporated as the nucleobase into a nucleoside (Scheme 1). Indeed, 1,6-naphthyridin-7(6*H*)-one scaffold shows a visible absorption, a solvatochromism, an acidochromism, large Stokes shifts, and high quantum yields depending on the solvent and media. These napthyridinones are also able to generate dual fluorescence in polar solvent from an intramolecular proton transfer at the excited state mechanism (ESIPT) coming from their lactim-lactam forms [41] (Scheme 1).

Therefore, the recent development in the field of triazole-linked fluorescent nucleoside conjugates and the photophysical properties of 1,6-naphthyridin-7(6*H*)-ones encouraged us to design and synthetize fused 1,6-naphthyridin-7(6*H*)-ones with triazole and to study their photophysical properties. We thus expect to develop a new class of fluorescent nucleoside analogues with potentially powerful biological properties as well as to use them as molecular probes to investigate enzyme binding sites or the structural characteristics of nucleic acids.

Herein, we described the synthesis of three original 1,2,3-triazole nucleoside conjugates (TzNat) containing fluorescent 1,6-naphthyridin-7(6*H*)-ones as the nucleobase using CuAAC click chemistry (Figure 1). Photophysical properties (absorption, emission quantum yield) of TzNat **A**, **B**, and **C** were studied in various solvents (Figure 1).

## 2. Results

### 2.1. Fluorescent Nucleosides Synthesis

Heteroaromatic nucleoside analogues possessing 1,2,3 triazoles as a linker between the nucleobase and the ribose are generally designed and synthesized using CuAAC click chemistry [42,43,44] to be used as Furo[2,3-b]pyrazine nucleoside analogues [45], nucleoside–iridium conjugates [46], Hydroxyanthracene triazolyl glycoconjugates [47], or as ligands for Cu(I) catalysis in the synthesis of quinazolinones [48]. Therefore, to design the naphtyridinone–ribose conjugate TzNat, we chose to use a CuAAC click reaction. 

**Scheme 2:**
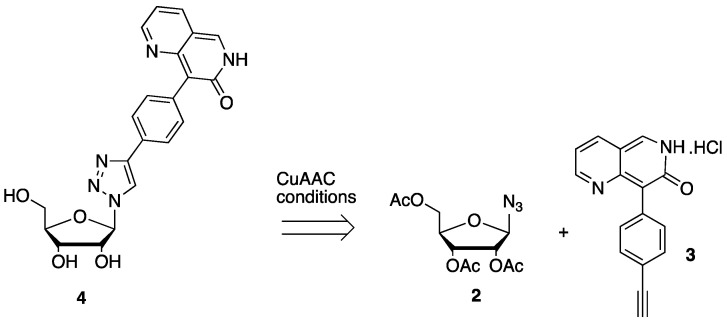
Retrosynthetic approach for the synthesis of 1,2,3-triazolyl naphtyridinone–ribose conjugate **4**.

First, in our earlier design we attempted to link the fluorescent naphtyridinone core at the 8-position to the ribose by a 1,2,3 triazole using a CuAAC reaction to get 1,2,3-triazolyl naphtyridinone–ribose conjugate **4**. To do so, we developed a strategy using azido ribofuranose **2** as an azide partner and 8-(4-ethynylphenyl)-6,7-dihydro-1,6-naphthyridin-7-one **3** as an alkyne partner (Scheme 2).

**Scheme 3:**
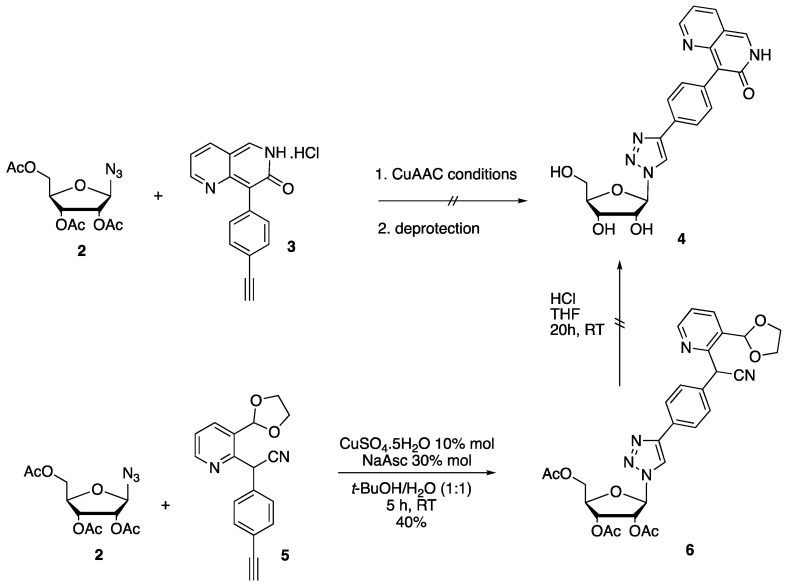
Attempts to use click reaction to prepare triazole conjugate **4** from acetylated ribosyl azide **2**.

Synthesis of 8-(4-ethynylphenyl)-1,6-naphthyridin-7(6*H*)-one **3** was achieved following our previously reported strategy from commercially available 2-chloro-3-formylpyridine. The azide partner **2** was prepared from 1,2,3,4-tetra-*O* acetyl β-d-ribofuranose following a reported procedure [49]. However, attempts to realize the CuAAC click reaction between the two partners failed. The classical procedure using a combination of CuSO_4_ and sodium ascorbate in tertbutanol/water at various temperatures failed. The use of CuI or more activated Cu(iMes)Cl in organic solvent with a base remained unsuccessful (Scheme 3 and Table S1 in Supplementary Materials). We hypothesized that the naphtyridinone bicycle **3** was not compatible with the click conditions because of the high reactivity of the pyridone moiety acting as a ligand with the copper atom. To overcome this issue, the click reaction was performed on the opened form **5** [41], the synthetic precursor of the 8-(4-ethynylphenyl)-1,6-naphthyridin-7(6*H*)-one **3** [41], and the azide **2** using standard conditions. Under these conditions, the click compound **6** was obtained in 40% yield. The last step consisted of an acidic treatment to promote the concomitant deprotection of the ribose, the acetal removal, and the nitrile function hydrolysis–cyclization. Unfortunately, this step did not give satisfactory results and led to an inseparable mixture containing a majority of the starting product, a very small amount of the expected product **4**, and a portion containing the mono-deacetylated sugar unit (Scheme 3).

**Scheme 4:**
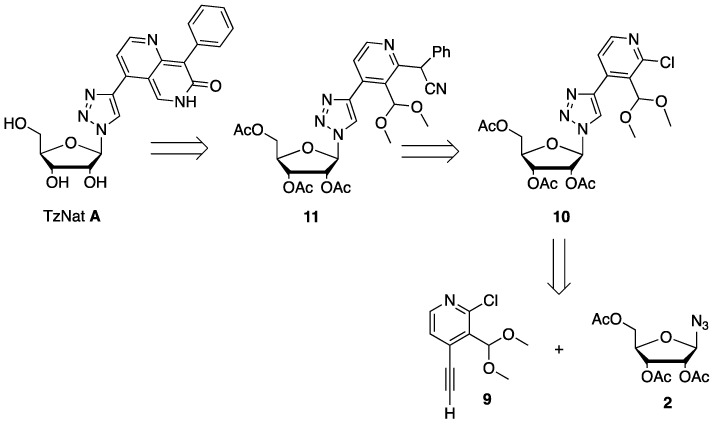
Retrosynthetic analysis of the synthesis of triazole conjugate TzNat **A**.

Encouraged by this last result, we slightly modified the strategy to target triazole conjugate **A** or TzNat **A**, which has the advantage of being easily modified at the 8-position of the naphthyridinone ring, and to allow further photophysical modification. Therefore, we have envisioned performing the click reaction with the 2-chloro-3-(dimethoxymethyl)-4-ethynylpyridine **9** as the alkyne partner and the azide ribose tetraacetate **2** in the first intention (Scheme 4).

**Scheme 5:**
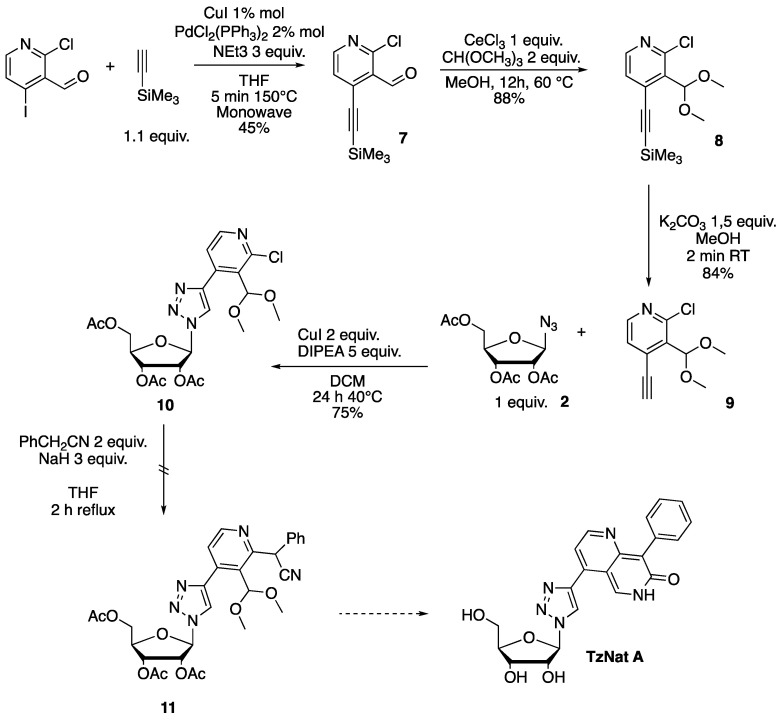
First attempt for the synthesis of TzNat **A**.

The synthesis of alkyne partner **9** was achieved in two steps. The mono-alkynylated product **7** was successfully prepared by a regioselective Sonogashira cross-coupling reaction performed in 5 min at 150 °C using a monowave apparatus. The aldehyde function of **7** was then protected as dimethoxyacetal under the mild conditions developed by Luche using methanol, cerium chloride, and trimethylorthoformate as the water scavenger [50]. Deprotection of the alkyne function appeared very sensitive due to the high reactivity of the triple bond. Two minutes were necessary to cleave the C–Si bond with K_2_CO_3_ in methanol at room temperature and obtain compound **9**. The ribofuranosyl azide partner **2** was reacted with **9** by a CuAAC click reaction with CuI, diisopropylethylene diamine in dichloromethane for 24 h at reflux, affording the desired triazole **10** in good yield. The triazole **10** was subsequently engaged in a nucleophilic aromatic substitution with phenylacetonitrile and sodium hydride. However, the reaction was unsuccessful. The presence of acetate protecting groups of the ribose part was suspected to be part of the observed lack of reactivity, due to its electron-withdrawing character and its propensity to be saponified under basic conditions (Scheme 5).

**Scheme 6:**
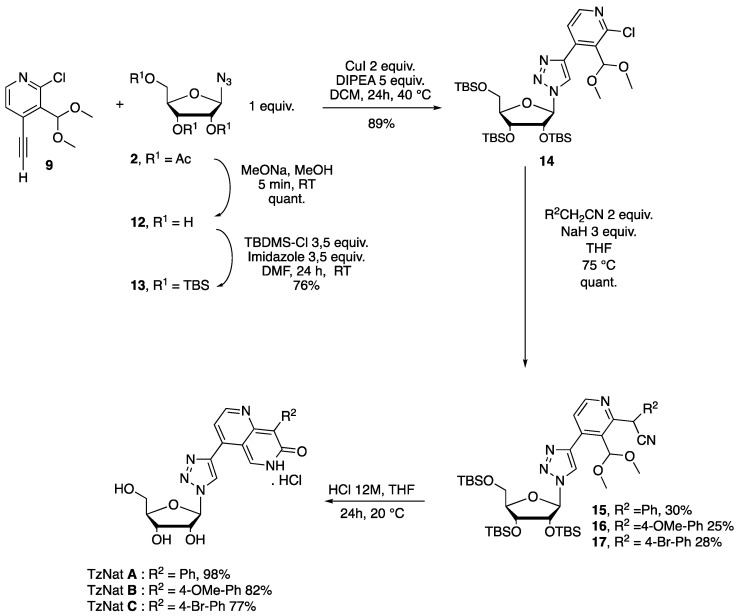
Successful strategy to prepare TzNat **A**, **B**, and **C**.

Therefore, we changed the ribose acetate protecting groups for the more stable tertbutyldimethylsilylether protecting groups. Starting from 2,3,5-triacetate-β-d-ribofuranosyl azide **2**, the protected tertbutyldimethylsilylether ribose azide **13** was obtained in two steps using sodium methoxide, followed by the addition of tertbutyldimethylsilylchloride and imidazole, with excellent yield (Scheme 6). Next, the CuAAC click reaction between the azide **13** and the alkyne **9** proceeded smoothly, affording the desired triazole **14** in good yield. The reaction of **14** with phenylacetonitrile in the presence of sodium hydride gave the substituted product **15** in 30% yield. Finally, the nucleoside napthyridinone TzNat **A** was obtained as its chlorohydrate salt in quantitative yield after acidic treatment of compound **15**. This strategy was employed to successfully prepare TzNAt **B** possessing a 4-methoxyphenyl substituent and TzNAt **C** bearing a 4-bromophenyl, starting from the same precursor **14** and using 4-methoxyphenylacetonitrile and 4-bromophenylacetonitrile respectively for the nucleophilic aromatic substitution step (Scheme 6). 

The structures of nucleosides TzNat **A**, **B**, and **C** were confirmed by NMR spectroscopy using 1D (^1^H, JMOD and ^13^C-DEPT) and 2D (COSY, HSQC, HMBC) experiments (See Supplementary Materials).

### 2.2. Optical Properties of TzNat Molecules

Optical properties of TzNat molecules as their chlorohydrate salts were studied in different solvents (Table 1). As their related compounds 1,6-Naphthyridin-7(6*H*)-ones contain a 2-hydroxypyridine system, the ribonucleoside TzNat may exist under lactim-enol and lactam-keto tautomeric forms in the ground state [41].

**Figure 2:**
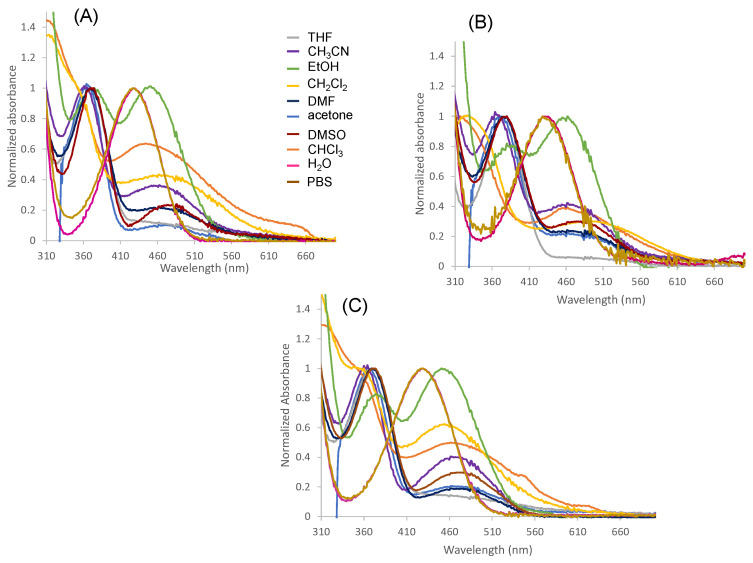
Normalized UV–Vis absorption spectra of TzNat **A**–**C** (at 10^−5^ M) in different solvents: (**A**) TzNat **A**, (**B**) TzNat **B**, (**C**) TzNat **C**.

As shown in the UV–Vis spectra in Figure 2, TzNat **A** to **C** show similar absorption profiles. The wavelength of the maximum absorption depends strongly on the polarity of the solvent. In non-polar solvent and polar aprotic solvent, the maximum absorption is centered between 320 and 386 nm, assigned to a π–π* transition with molar extinction coefficients ranging from 17,200 to 3200 M^−1^ cm^−1^. Interestingly, a bathochromic shift of this band is observed for all the TzNat from nonpolar solvent (CHCl_3_, CH_2_Cl_2_) to polar aprotic solvent (THF, DMSO, DMF, DMSO, acetone). This phenomenon is more pronounced for TzNat **B** possessing a para-methoxyphenyl group, for which a red-shift of 70 nm was found for the position of the maximum absorption in EtOH or DMSO relating to nonpolar solvent CHCl_3_ or CH_2_Cl_2_. This observed red-shift of the maximum absorption band from non-polar to polar solvent can be rationalized by the existence of an intramolecular charge transfer generated between the electron-donating group para-methoxy phenyl and the 1,2,3-triazole ring acting as an acceptor group [51].

A less intense band (ε = 6700 to 400 M^−1^·cm^−1^) centered at 459–474 nm is also observed in all solvents and is enhanced in non-polar solvent (CHCl_3_, CH_2_Cl_2_) as well as in protic solvent EtOH. However, in aqueous solvent (H_2_O and PBS), maximum absorption wavelengths of TzNat **A** to **C** ranging from 425 to 435 nm are recorded. This band is blue-shifted by 20 nm compared with that recorded in ethanol.

Overall, the UV properties of these molecules are in accordance with those recorded for 1,6-Naphthyridin-7(6*H*)-ones [41]. The maximum absorption ranging from 320 to 386 nm can be assigned to π→π* transition of the lactim form, whereas the absorption shift ranging from 425 to 474 nm could be related to the lactam form stabilized by intermolecular hydrogen bond with solvent.

**Figure 3:**
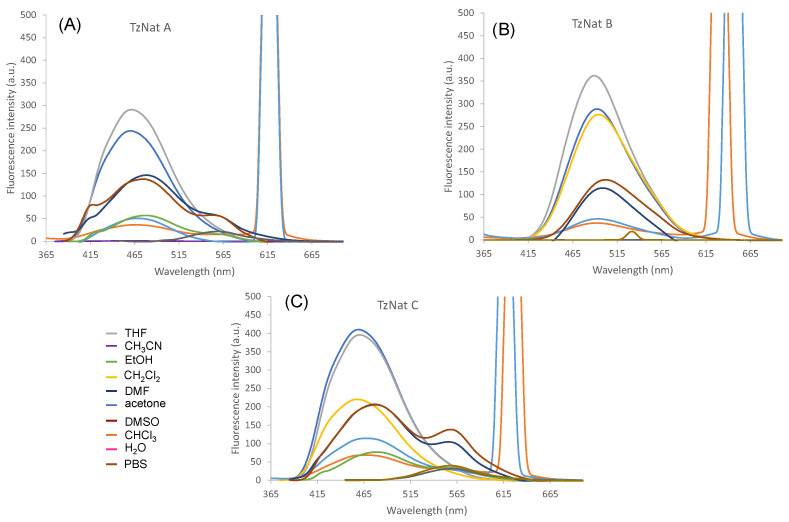
Fluorescence spectra of TzNat **A**–**C** (at C = 10^−5^ μM) in different solvents: (**A**) TzNat **A**, (**B**) TzNat **B**, (**C**) TzNat **C**. The sharp peaks around 615 nm are the 2nd harmonic of the excitation wavelength.

The fluorescence properties of TzNat molecules were studied in solvents with different polarity (Table 1). Stokes shifts and fluorescence quantum yields (Φ) were determined in each solvent. The corresponding spectra are represented in Figure 3. Spectra recorded upon excitation at 320–386 nm in polar aprotic solvent acetone, THF, DMF, and DMSO show an emission band for the three molecules ranging from 458–502 nm, with modest Stokes shifts of 5300 to 6500 cm^−1^ and quantum yield up to Φ = 0.34 for TzNat **B** in THF. In particular, TzNat **B** shows a red-shift of emission wavelength (+10 nm) and a higher quantum yield compared to TzNat **A** and **C** when placed in a medium of increased polarity. The red-shift can be explained by dipole–dipole interactions between the excited fluorophore and the surrounding molecules decreasing the excited-state energy, which is more noticeable for molecule **B** possessing a donor–acceptor system. In addition, the dramatic increase in emission quantum yield (×15) in DMF and DMSO observed for TzNat **B** is in accordance with the presence of an intramolecular charge transfer (ICT) stabilized in a high polar solvent as mentioned above.

Interestingly, for TzNat **A** and **C** possessing a phenyl and a para-bromophenyl group respectively, an additional emission band is observed in DMSO and DMF at 560 nm.

Surprisingly, upon excitation of the lactim form at 320–386 nm in non-polar solvent CHCl_3_ and CH_2_Cl_2_ and in polar protic solvent EtOH, TzNat **A**, **B**, and **C** show very weak fluorescence emission centered at 470–490 nm, accompanied by a drop in quantum efficiency (Φ < 0.01). Fluorescence quenching was also observed in aqueous solvent H_2_O and PBS buffer after excitation at 420–430 nm corresponding to the lactam form (Φ < 0.01).

By analogy with parent 1,6-Naphthyridin-7(6*H*)-ones, the solvatochromic behavior of TzNat molecules, showing distinct bands in absorption and emission depending on the polarity of the solvent, could be rationalized by the presence of lactam and lactim forms in equilibrium. A lactim form would correspond to the high energy band at 458–502 nm which is favored in polar aprotic solvent, with a red-shift for TzNat **B** of 30 nm related to TzNat **A** and **C**.

The lactam form of TzNat generated by a proton transfer of the lactim form isomer at the excited state is thought to be responsible for the low-energy band at 560 nm in polar solvent for molecules substituted by a phenyl (TzNat **A**) and a para-bromo phenyl group (TzNat **C**). However, the lactam form is not observed for TzNat **B** due to the ICT.

**Figure 4:**
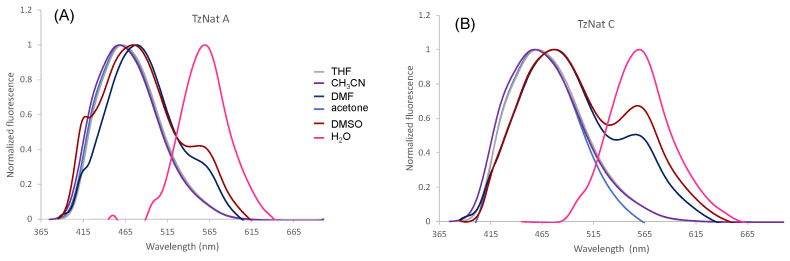
Normalized Fluorescence spectra of TzNat **A** and **C** (at 10^−5^ M) in polar solvents: (**A**) TzNat **A**, (**B**) TzNat **C**.

The quench of fluorescence in protic solvent could be explained by the nonradiative relaxation of the excited fluorophores enhanced by the polarity of the environment and hydrogen bonding with protic solvent molecules. Therefore, a twisted intramolecular charge transfer (TICT) resulting from the shift of the π-electron density on the donor–acceptor system at the excited state could also be involved to explain the quenching of the fluorescence in a protic solvent [52]. The twisted conformation is usually enhanced in a highly polar solvent possessing H-bonds and leads to full charge separation and a bathochromic shift. Indeed, in H_2_O, the weak emission band (Φ < 0.01) of the lactam form is observed red-shifted to 560 nm, and a second band of weak intensity centered at 560 nm is also observed in DMSO and DMF, which could be attributed to the TICT emission from the lactam form (Figure 4).

Nucleosides TzNat **A**, **B**, and **C** are environmentally sensitive fluorescent molecules, which are emissive in polar aprotic solvents with an enhanced quantum yield in the presence of charge transfer (TzNat **B**) and much less emissive in H_2_O. This dramatic change of emissivity can be used to probe interactions with surrounding molecules or as a reported group to study interactions with enzymes in incorporated oligomer [25].

Conformational studies of the triazole nucleosides TzNat **A**–**C** with computational methods will yield additional information about favored conformers, and help to rationalize the optical behavior and give evidence of the TICT mechanism. In-depth photo-physical studies of these compounds is currently under investigation.

## 3. Materials and Methods

### 3.1. General Information

All reagents and solvents were purchased from commercial suppliers, Sigma-Aldrich, TCI (Europe), Alfa Aesar, or Fluorochem. The monowave reactor is a conventionally heated synthesis reactor from Anton Paar. Infra-red spectra were recorded on ATR VariGATR crystal Ge. All reactions were monitored by TLC on silica gel 60 F254 plates and revealed using UV lamp (l 254 nm). Flash chromatography was carried out on a Merck silica gel 60 F 254. Reactions involving air- or moisture-sensitive reagents were carried out under an inert atmosphere (argon) with oven-dried glassware. THF was distillated over sodium/benzophenone. All final compounds were analyzed by high-resolution ESI mass spectrometry (HRMS) in MeOH using a LTQ-Orbitrap XL mass spectrometer (Thermo Scientific, San Jose, CA, USA) equipped with an electrospray ion source. NMR spectra were recorded on a Bruker Avance spectrometer at 300 or 400 MHz for ^1^H and 100 MHz for ^13^C. 2D NMR experiments such as ^1^H-^1^H COSY and ^1^H-^13^C HSQC experiments were performed to enable signal attributions. UV–Vis absorption spectra were measured using a Cary 50 (Varian) spectrophotometer at 20 °C. Emission spectra were performed on a JASCO J-815 CD spectrofluorometer at 20 °C. Quantum yields were determined with a Fluoromax+ (Jobin Yvon) equipped with a quanta phi integration sphere. Data were treated with the dedicated software provided by the supplier. Measurements were performed at room temperature. Synthesis and data concerning compounds **3** and compounds **5** are already described [29].

### 3.2. Attempts to Prepare Triazole Conjugate **4** from Acetylated Ribosyl Azide **2** and Compounds **5**

#### 3.2.1. Synthesis of 2,3,5-Tri-*O*-acetyl-β-d-ribofuranosyl Azide **2**

Trimethylsilyl azide (2.9 mL, 21.98 mmol) and tin chloride 1 M in heptane (4.7 mL, 4.7 mmol) were added to a stirred solution of 1,2,3,5-Tetra-*O*-acetyl-β-d-ribofuranose (5 g, 15.7 mmol) in dry DCM (40 mL). The reaction mixture was stirred at rt under argon for 20 h. The solution was washed with Na_2_CO_3_ + NaCl (10 mL + 20 mL), and the aqueous layer was extracted with DCM (2 × 30 mL). The combined organic layers were dried over MgSO_4_, filtered, and concentrated under vacuum. The resulting residue was purified by silica gel chromatography (Cyclohexane/EtOAc 7/3) to afford the desired compound as a colorless oil. M_pure_ = 4.8 g, Yield: quantitative. ^1^H NMR (300 MHz, Chloroform-d) δ 5.45–5.22 (m, 2H, H_5_), 5.12 (s, 1H, H_1_), 4.54–4.23 (m, 2H, H_3_/H_4_), 4.23–4.00 (m, 1H, H_2_), 2.21–1.93 (m, 9H, OAc). HRMS (ESI+): Calcd for C_11_H_15_N_3_O_7_Na 324.0802; found 324.0805 [M + Na]^+^. Spectral data of **2** were in agreement with the literature [49].

#### 3.2.2. Synthesis of (2*R*,3*R*,4*R*,5*R*)-2-(4-(4-((3-(1,3-dioxolan-2-yl)pyridin-2-yl)(cyano)methyl)phenyl)-1*H*-1,2,3-triazol-1-yl)-5-(acetoxymethyl)tetrahydrofuran-3,4-diyl Diacetate **6**

CuSO_4_·5H_2_O (4.2 mg, 10% mmol) and sodium ascorbate (10 mg, 30% mmol) were added to a stirred solution of compound **5** (50 mg, 0.17 mmol) and azide **2** (51 mg, 0.17 mmol) in *t*BuOH/H_2_O (150 μL *v*/*v* 1:1). The reaction mixture was stirred at rt for 5 h. Then, an aqueous solution of EDTA 0.1 M (2 mL) was added to the mixture, and the solution was extracted with EtOAc (3 × 5 mL). The combined organic layers were washed with an aqueous solution of EDTA 0.1 M (3 × 5 mL), dried over MgSO_4_, filtered, and concentrated under reduced pressure. The resulting residue was purified by silica gel chromatography (Cyclohexane/EtOAc 6/4, Rf = 0.21) to afford the title compound as a white oil. M = 40 mg. Yield: 40%. ^1^H NMR (400 MHz, Chloroform-*d*) δ 8.66 (d, *J* = 4.7 Hz, 1H, H_1_), 8.03 (s, 1H, H_18_), 7.91 (d, *J* = 7.6 Hz 1H, H_3_), 7.81 (d, *J* = 8.0 Hz, 2H, H_14/15_), 7.55 (d, *J* = 8.0 Hz, 2H, H_12/13_), 7.30 (dd, *J* = 8, 4.8Hz, 1H, H_2_), 6.18 (d, *J* = 3.7 Hz, 1H, H_1′_), 6.00–5.84 (m, 3H, H_6/9/4′_), 5.63 (t, *J* = 5.4 Hz, 1H, H_3′_), 4.50 (m, 1H, H_2′_), 4.25 (dd, *J* = 12.4, 4.4 Hz, 1H, H_5′a_), 4.17–4.04 (m, 5H, H_7/8/5′b_), 2.19–1.99 (m, 9H, OAc). ^13^C NMR (100 MHz, Chloroform-*d*) δ 170.7, 169.8, 169.7, 153.6, 150.7, 147.8, 136.1, 135.3, 131.5, 130.5, 129.3, 126.8, 123.5, 119.4, 101.2, 90.5, 81.4, 74.7, 71.17, 65.8, 65.7, 63.2, 60.8, 47.7, 21.1, 20.9, 20.8, 14.6. IR (cm^−1^): 2971, 2360, 1747, 1445, 1373, 1229, 1112, 1073, 805. HRMS (ESI+) Calcd for C_29_H_29_N_5_O_9_H 592.2038; found 592.2035 [M + H]^+^.

### 3.3. Synthesis of the Alkyne Partner **9** for the Click Reaction

#### 3.3.1. Synthesis of 2-chloro-4-((trimethylsilyl)ethynyl)nicotinaldehyde **7**

In a monowave vial (10 mL), 4-iodo-2-chloro-3-pyridine carboxaldehyde (1 g, 3.74 mmol) was dissolved in THF (6 mL). Ethynyltrimethylsilane (570 μL, 4.11 mmol), CuI (7 mg, 0.037 mmol), PdCl_2_(PPh_3_)_3_ (52 mg, 0.075 mmol), and NEt_3_ (1.58 mL, 11.22 mmol) were added into the vial. The reaction was heated using the mode “as fast as possible” at 150 °C for 10 min. The mixture was filtered on celite^®^ and washed with DCM. The residue was purified by silica gel chromatography (cyclohexane/Et_2_O 5%, Rf = 0.57) to afford the title compound as a yellow-brown solid. M = 400 mg. Yield: 45%. ^1^H NMR (300 MHz, Chloroform-*d*) δ 10.56 (s, 1H), 8.48 (d, *J* = 5.0 Hz, 1H), 7.42 (d, *J* = 5.1 Hz, 1H), 0.32 (s, 9H). ^13^C NMR (100 MHz, Chloroform-*d*) δ 188.2, 152.2, 152.0, 135.3, 128.9, 127.1, 109.8, 98.3, −0.6. HRMS (ESI+) Calcd for C_11_H_12_ClNOSiH 238.0449; found: 238.0450 [M + H]^+^.

#### 3.3.2. Synthesis of 2-chloro-3-(dimethoxymethyl)-4-((trimethylsilyl)ethynyl)pyridine **8**

Compound **7** (455 mg, 1.94 mmol) dissolved in MeOH (5 mL) was added to a stirred solution of CeCl_3_ (479 mg, 1.94 mmol) and trimethyl orthoformate CH(OCH_3_)_3_ (1.37 mL, 6.26 mmol) in MeOH (2 mL). The reaction mixture was heated at 60 °C until complete conversion of the starting material. The solution was neutralized with a saturated aqueous solution of NaHCO_3_ (10 mL). The aqueous layer was extracted with DCM (2 × 10 mL). The combined organic layers were dried over MgSO_4_, filtered, and concentrated under pressure to afford the title compound without further purification as an oil. M = 493 mg. Yield: 89%. Rf = 0.42 (cyclohexane/Et_2_O 8/2). ^1^H NMR (300 MHz, Chloroform-d) δ 8.30 (d, *J* = 5.0 Hz, 1H), 7.29 (d, *J* = 5.0 Hz, 1H), 5.81 (s, 1H), 3.49 (s, 6H), 0.31 (s, 9H). ^13^C NMR (100 MHz, Chloroform-d) δ 149.2, 134.0, 133.0, 126.7, 106.8, 104.1, 100.2, 56.1, −0.1. HRMS (ESI+) Calcd for C_13_H_18_ClNO_2_SiH 284.0868; found: 284.0869 [M + H]^+^.

#### 3.3.3. Synthesis of 2-chloro-3-(dimethoxymethyl)-4-ethynylpyridine **9**

K_2_CO_3_ (623 mg, 4.5 mmol) was added to a stirred solution of compound **8** (850 mg, 3 mmol) in MeOH (20 mL). The reaction mixture was stirred at rt for 2 min. The solution was immediately quenched with water (20 mL). The aqueous layer was extracted with DCM (3 × 20 mL). The combined organic layers were dried over MgSO_4_, filtered, and concentrated under reduced pressure to afford a crude product which was engaged in the next step without further purification. M = 556 mg. Yield: 84%. Rf = 0.25 (cyclohexane/Et_2_O 8/2). ^1^H NMR (400 MHz, Chloroform-*d*) δ 8.32 (d, *J* = 5.0 Hz, 1H), 7.36 (d, *J* = 5.0 Hz, 1H), 5.76 (s, 1H), 3.60 (s, 1H), 3.49 (s, 6H). ^13^C NMR (100 MHz, Chloroform-*d*) δ 151.0, 148.8, 132.9, 132.2, 127.3, 103.6, 87.3, 78.9, 55.8. IR (cm^−1^): 3217, 2995, 2940, 2838, 2107, 1573, 1531, 1371, 1210, 1184, 1060, 961, 827.HRMS (ESI+) Calcd for C_10_H_10_ClNO_2_H 212.0473, found: 212.0473 [M + H]^+^.

### 3.4. Synthesis of Target 1,2,3 Triazole Nucleoside TzNat **A**, **B**, and **C**

#### 3.4.1. Synthesis of (2*R*,3*R*,4*R*,5*R*)-2-(acetoxymethyl)-5-(4-(2-chloro-3-(dimethoxymethyl)pyridin-4-yl)-1*H*-1,2,3-triazol-1-yl)tetrahydrofuran-3,4-diyl Diacetate **10**

CuI (79 mg, 0.42 mmol), DIPEA (182 μL, 1.05 mmol) and compound **9** (65 mg, 0.21 mmol) were added to a stirred solution of compound **2** (45.5 mg, 0.21 mmol) in DCM (3 mL). The reaction mixture was stirred at 40 °C for 7 h. Then an aqueous solution of EDTA 0.1 M (10 mL) was added to the mixture which was stirred for 1 h until the organic layer was colorless. The organic layer was dried over MgSO_4_, filtered and concentrated under reduced pressure. The crude was purified by silica gel chromatography (Cyclohexane/EtOAc 1/1) to afford the title compound as a colorless oil, m_pure_ = 70 mg. Yield: 65%. ^1^H NMR (300 MHz, Chloroform-*d*) δ 8.50 (s, 1H), 8.38 (d, *J* = 5.1 Hz, 1H), 8.04 (d, *J* = 5.1 Hz, 1H), 6.18 (d, *J* = 3.6 Hz, 1H), 5.90–5.84 (m, 2H), 5.64 (t, *J* = 5.4 Hz, 1H), 4.51–4.39 (m, 2H,), 4.28–4.13 (m, 1H), 3.39 (s, 6H), 2.14–2.00 (m, 9H). ^13^C NMR (100 MHz, Chloroform-*d*) δ 170.4, 169.4, 169.2, 151.7, 149.5, 143.2, 140.3 126.6, 123.9, 104.8, 90.0, 80.9, 77.2, 74.4, 70.8, 62.9, 56.1, 56.0, 20.6, 20.4, 20.4. HRMS (ESI+) Calcd for C_21_H_25_ClN_4_O_9_Na 535.1202; found: 535.1199 [M + Na]^+^.

#### 3.4.2. Synthesis of β-d-ribofuranosyl Azide **12**

A freshly prepared solution of MeONa (1 M in MeOH) was added to a stirred solution of 2,3,5-tri-*O*-acetate-β-d-ribofuranosyl azide (4.8 g, 15.9 mmol) in MeOH (90 mL). The reaction mixture was stirred for 5 min at rt and quenched with DOWEX resin. The suspension was vigorously stirred for 10 min, and the resin was filtered and washed with DCM. The desired compound was obtained as a pure colorless oil without purification. M_pure_ = 2.7 g. Yield: quantitative. ^1^H NMR (300 MHz, Methanol-d4) δ 5.21 (d, *J =* 1.9 Hz, 1H), 4.10–4.05 (m, 1H), 4.02–3.97 (m, 1H), 3.84–3.82 (m, 1H), 3,76 (m, 1H), 3.63 (dd, *J =* 12.0, 5.5 Hz, 1H) HRMS (ESI+): Calcd for C_5_H_8_N_3_O_4_ 174.0520; found 174.0522 [M − H]^−^. Spectral data of **12** were in agreement with the literature [53].

#### 3.4.3. Synthesis of 2,3,5-Tri-*O*-(tertbutyldimethylsilyle)-β-d-ribofuranosyl Azide **13**

Imidazole (3.9 g, 57 mmol) and tertbutyldimethylchlorosilane (8.2 g, 54 mmol) were added to a stirred solution of D-ribofuranosyl azide (2.7 g, 15.4 mmol) in DMF (30 mL). The reaction mixture was stirred for 24 h at rt. The mixture was quenched with a saturated aqueous solution of NaHCO_3_, and the aqueous layer was extracted with DCM (3 × 40 mL). The combined organic layers were washed with water (3 × 50 mL), dried over MgSO_4_, filtered, and concentrated under reduced pressure. The resulting residue was purified by silica gel chromatography (Cyclohexane/Et_2_O 5%, Rf = 0.20) to afford the title compound as a white solid. M = 6.1 g. Yield: 76%. ^1^H NMR (300 MHz, Chloroform-*d*) δ 5.10 (d, *J* = 2.7, 1H, H_1′_), 4.20 (dd, *J* = 5.9, 4.0 Hz, 1H, H_3′_), 4.01 (dt, *J* = 6.2, 3.2, 1H, H_4′_), 3.82 (m, 1H, H_2′_), 3.82 (dd, *J* = 11.5, 3.4 Hz, 1H, H_5′_) 3.67 (dd, *J* = 11.5, 3.4 Hz, 1H, H_5′_), 0.92 (s, 9H, H_7′_), 0.91 (s, 9H, H_7′_), 0.90 (s, 9H, H_7′_), 0.11–0.08 (s, 18H, H_6′_). ^13^C NMR (100 MHz, Chloroform-*d*) δ 94.7 (CH_1′_), 84.3 (CH4′), 76.5 (CH_2′_), 71.1 (CH_3′_), 62.1(CH_5′_), 25.9 (CH_7′_), 25.8 (CH_7′_), 25.8 (CH_7′_), −4.4, −4.6, −5.5 (CH_6′_). IR (cm^−1^): 2951, 2931, 2859, 2117, 1472, 1142, 1128, 1073, 999, 777. HRMS (ESI+): Calcd for C_23_H_51_N_3_O_4_Si_3_Na 540.3080. Found 540.3080 [M + Na]^+^.

#### 3.4.4. Synthesis of 4-(1-((2*R*,3*R*,4*R*,5*R*)-3,4-bis((*tert*-butyldimethylsilyl)oxy)-5-(((*tert*-butyldimethylsilyl)oxy)methyl)tetrahydrofuran-2-yl)-1*H*-1,2,3-triazol-4-yl)-2-chloro-3-(dimethoxymethyl)pyridine **14**

Under inert condition, in a sealed tube, CuI (1.06 g, 5.56 mmol), DIPEA (2.40 mL, 14 mmol), and compound **13** (1.58 g, 3.06 mmol) were added portionwise to a stirred solution of compound **9** (588 mg, 2.78 mmol) in DCM (23 mL). The reaction mixture was stirred at 40 °C for 24 h. Then, the solution was cooled to room temperature and an aqueous solution of EDTA 0.1 M (50 mL) was added to the mixture, which was stirred for 1 h until the organic layer was colorless. The aqueous layer was extracted with DCM (3 × 20 mL). The combined organic layers were washed with a solution of EDTA 0.1 M until the aqueous layer was colorless, with water (20 mL) and brine (20 mL). Then, it was dried over MgSO_4_, filtered, and concentrated under reduced pressure. The crude was purified by silica gel chromatography (cyclohexane/Et_2_O 20%, Rf = 0.30) to afford the title compound as a yellow oil. M = 1.8 g. Yield: 89%. ^1^H NMR (400 MHz, Chloroform-*d*) δ 8.40 (s, 1H, H_10_), 8.38 (d, *J =* 5.1 Hz, 1H, H_5_), 8.01 (d, *J* = 5.1 Hz, 1H, H_4_), 5.91 (d, *J* = 4.8 Hz, 1H, H_1′_), 5.84 (s, 1H, H_6_), 4.80 (t, *J* = 4.8 Hz, H_2′_), 4.35 (t, *J* = 3.9 Hz, 1H, H_3′_), 4.15 (q, *J* = 4.6, 4.3 Hz, 1H, H_4′_), 3.80 (dd, *J* = 11.1, 5.4 Hz, 1H, H_5′_), 3.75 (dd, *J* = 11.1, 5.4 Hz, 1H, H_5′_), 3.37 (s, 3H, H_7_), 3.41 (s, 3H, H_8_), 0.97 (s, 9H, H_7′_), 0.87 (s, 9H, H_7′_), 0.84 (s, 9H, H_7′_), 0.12 (s, 6H, H_6′_), 0.04 (s, 3H, H_6′_), 0.03 (s, 3H, H_6′_), 0.01 (s, 3H, H_6′_), −0,14 (s, 3H, H_6′_). ^13^C NMR (100 MHz, Chloroform-*d*) δ 151.8 (C_3_), 149.5 (CH_5_), 142.8 (C_1_), 140.9 (C_9_), 128.0 (C_2_), 127.4 (CH_10_), 124.1 (CH_4_), 104.9 (CH_6_), 92.3 (CH_1′_), 86.3 (CH_4′_), 76.3 (CH_2′_), 72.5 (CH_3′_), 63.1 (CH_5′_), 56.2 (CH_7/8_), 56.1 (CH_7/8_), 27.0 (CH_7′_), 18.5 (C_8′_), 18.1 (C_8′_), 18.1 (C_8′_), −4.3 (CH_6′_), −4.6 (CH_6′_), −5.0 (CH_6′_), −5,3 (CH_6′_), −5.4 (CH_6′_), 5.86 (CH_6′_). IR (cm^−1^): 2953, 2930, 2896, 2857, 1253, 1110, 1076, 836, 777. HRMS (ESI+) Calcd for C_33_H_61_ClN_4_O_6_Si_3_Na 751.3480. Found 751.3480 [M + Na]^+^.

#### 3.4.5. Synthesis of 2-(4-(1-((2*R*,3*R*,4*R*,5*R*)-3,4-bis((*tert*-butyldimethylsilyl)oxy)-5-(((*tert*-butyldimethylsilyl)oxy)methyl)tetrahydrofuran-2-yl)-1*H*-1,2,3-triazol-4-yl)-3-(dimethoxymethyl)pyridin-2-yl)-2-phenylacetonitrile **15**

Under inert conditions, in a sealed tube, sodium hydride (3 equiv., 60% in oil) was added to a stirred solution of compound 14 (255.3 mg, 0.35 mmol) in dry THF (0.18 M). Phenylacetonitrile (80 μL, 0.7 mmol.) was added in one portion, and the reaction mixture was refluxed for 20 h. The solution was cooled to room temperature and quenched by addition of water. The compound was extracted three times with ethyl acetate. The combined organic layers were washed with brine, dried over MgSO_4_, filtered, and concentrated under reduced pressure. The resulting residue was purified by silica gel chromatography (Cyclohexane/Et_2_O 8/2, Rf = 0.21) to afford the title compound as a yellow oil and as a mixture of two diastereoisomers: 55/45. M = 85 mg. Yield: 30%. ^1^H NMR (400 MHz, CDCl_3_) δ 8.62 (d, *J* = 2.5 Hz, 1H, H_5_, dia 1), 8.60 (d, *J* = 2.5 Hz, 1H, H_5_, dia 2), 8.09 (s, 1H, H_10_, dia 1 or dia 2), 8.08 (s, 1H, H_10_, dia 1 or dia 2), 7.60–7.58 (m, 2H, H_12,16_, dia 1 or dia 2), 7.58–7.56 (m, 2H, H_12,16_, dia 1 or dia 2), 7.37–7.34 (m, 2H, H_13,15_, dia 1), 7.34–7.31 (m, 2H, H_13,15_, dia 2), 7.30–7.28 (m, 2H, H_14_, dia 1), 7.28–7.26 (m, 2H, H_14_, dia 2), 7.22 (d, *J* = 5.0 Hz, 1H, H_4_, dia 1 or dia 2), 7.20 (d, *J* = 5.0 Hz, 1H, H_4_, dia 1 or dia 2), 6.49 (s, 1H, H_11_, dia 1 or dia 2), 6.48 (s, 1H, H_11_, dia 1 or dia 2), 6.08 (d, *J* = 2.4 Hz, 1H, H_1′_, dia 1), 6.06 (d, *J* = 2.3 Hz, 1H, H_1′_, dia 2), 5.83 (s, 1H, H_6_, dia 1 or dia 2), 5.83 (s, 1H, H_6_, dia 1 or dia 2), 4.64–4.59 (m, 1H, H_2′_, dia 2), 4.60–4.58 (m, 1H, H_2′_, dia 1), 4.30–4.27 (m, 1H, H_3′_, dia 2), 4.27–4.24 (m, 1H, H_3′_, dia 1), 4.20–4.18 (m, 1H, H_4′_, dia 1), 4.18–4.16 (m, 1H, H_4′_, dia 2), 3.93–3.91 (m, 1H, H_5′_, dia 2), 3.90–3.88 (m, 1H, H_5′_, dia 1), 3.79 (d, *J* = 2.9 Hz, 1H, H_5′_, dia 1), 3.77 (d, *J* = 2.9 Hz, 1H, H_5′_, dia 2), 3.49 (s, 3H, H_7_ or H_8_, dia 1 or dia 2), 3.45 (s, 3H, H_7_ or H_8_, dia 1 or dia 2), 3.42 (s, 3H, H_7_ or H_8_, dia 1 or dia 2), 3.40 (s, 3H, H_7_ or H_8_, dia 1 or dia 2), 0.95 (s, 1H, H_7′_, dia 1 or dia 2), 0.94 (s, 9H, H_7′_, dia 1 or dia 2), 0.89 (s, 9H, H_7′_, dia 1 or dia 2), 0.88 (s, 9H, H_7′_, dia 1 or dia 2), 0.86 (s, 9H, H_7′_, dia 1 or dia 2), 0.85 (s, 9H, H_7′_, dia 1 or dia 2), 0.13 (s, 3H, H_6′_, dia 1 or dia 2), 0.12 (s, 3H, H_6′_, dia 1 or dia 2), 0.12 (s, 3H, H_6′_, dia 1 or dia 2), 0.11 (s, 3H, H_6′_, dia 1 or dia 2), 0.08 (s, 3H, H_6′_, dia 1 or dia 2), 0.08 (s, 3H, H_6′_, dia 1 or dia 2), 0.08 (s, 3H, H_6′_, dia 1 or dia 2), 0.07 (s, 3H, H_6′_, dia 1 or dia 2), 0.01 (s, 3H, H_6′_, dia 1), 0.00 (s, 3H, H_6′_, dia 2), −0.14 (s, 3H H_6′_, dia 1), −0.16 (s, 3H, H_6′_, dia 2). ^13^C NMR (101 MHz, CDCl_3_) δ 156.3 (C_1_, dia 1 or dia 2), 156.3 (C_1_, dia 1 or dia 2), 149.8 (C_5_, dia 1 or dia 2), 149.8 (C_5_, dia 1 or dia 2), 144.6 (C_9_, dia 1 or dia 2), 144.6 (C_9_, dia 1 or dia 2), 138.8 (C_3_, dia 1 or dia 2), 138.7 (C_3_, dia 1 or dia 2), 136.0 (C_17_, dia 1 or dia 2), 136.0 (C_17_, dia 1 or dia 2), 130.1 (C_2_, dia 1 or dia 2), 130.1 (C_2_, dia 1 or dia 2), 128.8 (C_12,16_ or C_13,15_, dia 1 or dia 2), 128.7 (C_12,16_ or C_13,15_, dia 1 or dia 2), 128.7 (C_12,16_ or C_13,15_, dia 1 or dia 2), 127.8 (C_12,16_ or C_13,15_, dia 1 or dia 2), 127.8 (C_14_, dia 1 or dia 2), 127.8 (C_14_, dia 1 or dia 2), 123.0 (C_4_, dia 1 or dia 2), 122.9 (C_4_, dia 1 or dia 2), 122.6 (C_10_, dia 1 or dia 2), 122.6 (C_10_, dia 1 or dia 2), 120.3 (CN, dia 1 or dia 2), 120.2 (CN, dia 1 or dia 2), 103.2 (C_6_, dia 1 or dia 2), 103.2 (C_6_, dia 1 or dia 2), 92.9 (C_1′_, dia 1 or dia 2), 92.8 (C_1′_, dia 1 or dia 2), 86.8 (C_4′_, dia 1 or dia 2), 86.6 (C_4′_, dia 1 or dia 2), 77.2 (C_2′_, dia 1 or dia 2), 77.2 (C_2′_, dia 1 or dia 2), 72.6 (C_3′_, dia 1 or dia 2), 72.4 (C_3′_, dia 1 or dia 2), 62.9 (C_5′_, dia 1 or dia 2), 62.8 (C_5′_, dia 1 or dia 2), 56.3 (C_7_ or C_8_, dia 1 or dia 2), 56.2 (C_7_ or C_8_, dia 1 or dia 2), 55.9 (C_7_ or C_8_, dia 1 or dia 2), 55.9 (C_7_ or C_8_, dia 1 or dia 2), 41.6 (C_11_, dia 1 or dia 2), 41.5 (C_11_, dia 1 or dia 2), 26.1 (3C_7′_, dia 1 or dia 2), 26.1 (3C_7′_, dia 1 or dia 2), 25.9 (3C_7′_, dia 1 or dia 2), 25.9 (3C_7′_, dia 1 or dia 2), 25.9 (3C_7′_, dia 1 or dia 2), 25.9 (3C_7′_, dia 1 or dia 2), 18.1 (3C_8′_, dia 1 or dia 2), 18.1 (3C_8′_, dia 1 or dia 2), −4.3 (2C_6′_, dia 1 or dia 2), −4.3 (2C_6′_, dia 1 or dia 2), −4.5 (2C_6′_, dia 1 or dia 2), −4.5 (2C_6′_, dia 1 or dia 2), −5.0 (2C_6′_, dia 1 or dia 2), −5.0 (2C_6′_, dia 1 or dia 2). IR (cm^−1^): 2953, 2930, 2857, 2359, 2341, 2254, 1595, 1494, 1259, 1253, 1109, 1074, 836, 779. HRMS (ESI) *m*/*z*: [M + H]^+^ Calcd for C_41_H_67_N_5_O_6_Si_3_H 810.4472. Found 810.4476.

#### 3.4.6. Synthesis of 2-(4-(1-((2*R*,3*R*,4*R*,5*R*)-3,4-bis((*tert*-butyldimethylsilyl)oxy)-5-(((*tert*-butyldimethylsilyl)oxy)methyl)tetrahydrofuran-2-yl)-1*H*-1,2,3-triazol-4-yl)-3-(dimethoxymethyl)pyridin-2-yl)-2-(4-methoxyphenyl)acetonitrile **16**

Under inert condition, in a sealed tube, sodium hydride (3 equiv., 60% in oil) was added to a stirred solution of compound **14** (295 mg, 0.4 mmol) in dry THF (0.2 M). 4-methoxyphenylacetonitrile (108.5 μL, 0.80 mmol) was added in one portion, and the reaction mixture was refluxed for 48 h. The solution was cooled to room temperature and quenched by addition of water. The compound was extracted three times with ethyl acetate. The combined organic layers were washed with brine, dried over MgSO_4_, filtered, and concentrated under reduced pressure. The resulting residue was purified by silica gel chromatography (Cyclohexane/Et_2_O 7/3, Rf = 0.28) to afford the title compound as a yellow oil and as a mixture of two diastereoisomers: M = 85.2 mg. Yield: 25%. ^1^H NMR (300 MHz, CDCl_3_) δ 8.62 (d, *J* = 1.8 Hz, 1H, H_5_, dia 1), 8.61 (d, *J* = 1.8 Hz, 1H, H_5_, dia 2), 8.09 (s, 1H, H_10_, dia 1 or dia 2), 8.08 (s, 1H, H_10_, dia 1 or dia 2), 7.55–7.51 (m, 2H, H_13,17_, dia 1 or dia 2), 7.51–7.48 (m, 2H, H_13,17_, dia 1 or dia 2), 7.21 (d, *J* = 5.2 Hz, 1H, H_4_, dia 2), 7.19 (d, *J* = 5.6 Hz, 1H, H_4_, dia 1), 6.89–6.86 (m, 2H, H_13,16_, dia 1 or dia 2), 6.86–6.83 (m, 2H, H_13,16_, dia 1 or dia 2), 6.42 (s, 1H, H_11_, dia 1 or dia 2), 6.41 (s, 1H, H_11_, dia 1 or dia 2), 6.08 (d, *J* = 1.9 Hz, 1H, H_1′_, dia 1), 6.06 (d, *J* = 1.7 Hz, 1H, H_1′_, dia 2), 5.82 (s, 1H, H_6_, dia 1 or dia 2), 5.82 (s, 1H, H_6_, dia 1 or dia 2), 4.64–4.60 (m, 1H, H_2′_, dia 2), 4.60–4.56 (m, 1H, H_2′_, dia 1), 4.30–4.26 (m, 1H, H_3′_, dia 2), 4.26–4.23 (m, 1H, H_3′_, dia 1), 4.20–4.18 (m, 1H, H_4′_, dia 1), 4.18–4.15 (m, 1H, H_4′_, dia 2), 3.94–3.90 (m, 1H, H_5′_, dia 2), 3.90–3.87 (m, 1H, H_5′_, dia 1), 3.83–3.74 (m, 1H, H_5′_, dia 1), 3.78 (s, 3H, H_18_, dia 1 or dia 2), 3.78 (s, 3H, H_18_, dia 1 or dia 2), 3.77–3.74 (m, 1H, H_5′_, dia 2), 3.49 (s, 3H, H_7_ or H_8_, dia 1 or dia 2), 3.45 (s, 3H, H_7_ or H_8_, dia 1 or dia 2), 3.41 (s, 3H, H_7_ or H_8_, dia 1 or dia 2), 3.39 (s, 3H, H_7_ or H_8_, dia 1 or dia 2), 0.94 (s, 9H, H_7′_, dia 1 or dia 2), 0.94 (s, 9H, H_7′_, dia 1 or dia 2), 0.89 (s, 9H, H_7′_, dia 1 or dia 2), 0.87 (s, 9H, H_7′_, dia 1 or dia 2), 0.86 (s, 9H, H_7′_, dia 1 or dia 2), 0.85 (s, 9H, H_7′_, dia 1 or dia 2), 0.12 (s, 3H, H_6′_, dia 1 or dia 2), 0.12 (s, 3H, H_6′_, dia 1 or dia 2), 0.11 (s, 3H, H_6′_, dia 1 or dia 2), 0.11 (s, 3H, H_6′_, dia 1 or dia 2), 0.08 (s, 3H, H_6′_, dia 1 or dia 2), 0.08 (s, 3H, H_6′_, dia 1 or dia 2), 0.07 (s, 3H, H_6′_, dia 1 or dia 2), 0.00 (s, 3H, H_6′_, dia 1 or dia 2), 0.00 (s, 3H, H_6′_, dia 1 or dia 2), −0.15 (s, 3H, H_6′_, dia 1 or dia 2), −0.17 (s, 3H, H_6′_, dia 1 or dia 2). ^13^C NMR (75 MHz, CDCl_3_) δ 159.2 (C_15_, dia 1 or dia 2), 159.1 (C_15_, dia 1 or dia 2), 156.5 (C_1_, dia 1 or dia 2), 156.5 (C_1_, dia 1 or dia 2), 149.8 (C_5_, dia 1 or dia 2), 149.8 (C_5_, dia 1 or dia 2), 144.7 (C_9_, dia 1 or dia 2), 144.6 (C_9_, dia 1 or dia 2), 138.7 (C_3_, dia 1 or dia 2), 138.7 (C_3_, dia 1 or dia 2), 130.0 (C_2_, dia 1 or dia 2), 130.0 (C_2_, dia 1 or dia 2), 129.9 (C_13,14_, dia 1 or dia 2), 129.9 (C_13,14_, dia 1 or dia 2), 128.1 (C_12_, dia 1 or dia 2), 128.1 (C_12_, dia 1 or dia 2), 122.9 (C_4_, dia 1 or dia 2), 122.9 (C_4_, dia 1 or dia 2), 122.6 (C_10_, dia 1 or dia 2), 122.5 (C_10_, dia 1 or dia 2), 120.5 (CN, dia 1 or dia 2), 120.5 (CN, dia 1 or dia 2), 114.0 (C_14,16_, dia 1 or dia 2), 114.0 (C_14,16_, dia 1 or dia 2), 103.2 (C_6_, dia 1 or dia 2), 103.2 (C_6_, dia 1 or dia 2), 92.9 (C_1′_, dia 1 or dia 2), 92.8 (C_1′_, dia 1 or dia 2), 86.8 (C_4′_, dia 1 or dia 2), 86.6 (C_4′_, dia 1 or dia 2), 77.4 (C_2′_, dia 1 or dia 2), 77.3 (C_2′_, dia 1 or dia 2), 72.6 (C_3′_, dia 1 or dia 2), 72.4 (C_3′_, dia 1 or dia 2), 62.9 (C_5′_, dia 1 or dia 2), 62.8 (C_5′_, dia 1 or dia 2), 56.2 (C_7_ or C_8_, dia 1 or dia 2), 56.2 (C_7_ or C_8_, dia 1 or dia 2), 55.9 (C_7_ or C_8_, dia 1 or dia 2), 55.9 (C_7_ or C_8_, dia 1 or dia 2), 55.4 (C_18_, dia 1 or dia 2), 55.4 (C_18_, dia 1 or dia 2), 40.7 (C_11_, dia 1 or dia 2), 40.7 (C_11_, dia 1 or dia 2), 26.1 (3C, C_7′_, dia 1 or dia 2), 26.1 (3C, C_7′_, dia 1 or dia 2), 25.9 (3C, C_7′_, dia 1 or dia 2), 25.9 (3C, C_7′_, dia 1 or dia 2), 25.8 (3C, C_7′_, dia 1 or dia 2), 18.5 (C_8′_, dia 1 or dia 2), 18.5 (C_8′_, dia 1 or dia 2), 18.2 (C_8′_, dia 1 or dia 2), 18.2 (C_8′_, dia 1 or dia 2), 18.1 (C_8′_, dia 1 or dia 2), 18.1 (C_8′_, dia 1 or dia 2), −4.3 (C_6′_, dia 1 or dia 2), −4.3 (C_6′_, dia 1 or dia 2), −4.5 (C_6′_, dia 1 or dia 2), −4.5 (C_6′_, dia 1 or dia 2), −4.5 (C_6′_, dia 1 or dia 2), −4.5 (C_6′_, dia 1 or dia 2), −5.0 (C_6′_, dia 1 or dia 2), −5.0 (C_6′_, dia 1 or dia 2), −5.1 (C_6′_, dia 1 or dia 2), −5.1 (C_6′_, dia 1 or dia 2) −5.3 (C_6′_, dia 1 or dia 2), −5.3 (C_6′_, dia 1 or dia 2). IR (cm^−1^): 2952, 2930, 2857, 2359, 2342, 2254, 1596, 1254, 1108, 1073, 836, 778. HRMS (ESI) *m*/*z*: [M + H]^+^ Calcd for C_42_H_69_N_5_O_7_Si_3_H 840.4578. Found 840.4581.

#### 3.4.7. Synthesis of 2-(4-(1-((2*R*,3*R*,4*R*,5*R*)-3,4-bis((*tert*-butyldimethylsilyl)oxy)-5-(((*tert*-butyldimethylsilyl)oxy)methyl)tetrahydrofuran-2-yl)-1*H*-1,2,3-triazol-4-yl)-3-(dimethoxymethyl)pyridin-2-yl)-2-(4-bromophenyl)acetonitrile **17**

Under inert condition, in a sealed tube, sodium hydride (3 equiv., 60% in oil) was added to a stirred solution of compound 14 (297 mg, 0.41 mmol) in dry THF (0.2 M). 4-bromophenylacetonitrile (155.8 mg, 0.82 mmol) was added in one portion, and the reaction mixture was refluxed for 72 h. The solution was cooled to room temperature and quenched by addition of water. The compound was extracted three times with ethyl acetate. The combined organic layers were washed with brine, dried over MgSO_4_, filtered, and concentrated under reduced pressure. The resulting residue was purified by silica gel chromatography (Cyclohexane/Et_2_O 7/3, Rf = 0.24) to afford the title compound as an orange oil and as a mixture of two diastereoisomers. M = 102 mg. Yield: 28%. ^1^H NMR (300 MHz, CDCl_3_) δ 8.60 (d, *J* = 1.4 Hz, 1H, H_5_, dia 1), 8.59 (d, *J* = 1.4 Hz, 1H, H_5_, dia 2), 8.10 (s, 1H, H_10_, dia 1 or dia 2), 8.10 (s, 1H, H_10_, dia 1 or dia 2), 7.48–7.47 (m, 4H, H_13,14,16,17_, dia 1 or dia 2), 7.47–7.46 (m, 4H, H_13,14,16,17_, dia 1 or dia 2), 7.21 (d, *J* = 5.0 Hz, 1H, H_4_, dia 2), 7.19 (d, *J* = 5.0 Hz, 1H, H_4_, dia 1), 6.45 (s, 1H, H_11_, dia 1 or dia 2), 6.44 (s, 1H, H_11_, dia 1 or dia 2), 6.09 (d, *J* = 1.6 Hz, 1H, H_1′_, dia 1 or dia 2), 6.07 (d, *J* = 1.4 Hz, 1H, H_1′_, dia 1 or dia 2), 5.85 (s, 1H, H_6_, dia 1 or dia 2), 5.85 (s, 1H, H_6_, dia 1 or dia 2), 4.61 (d, *J* = 4.6 Hz, 1H, H_2′_, dia 1), 4.58 (d, *J* = 4.8 Hz, 1H, H_2′_, dia 2), 4.29–4.26 (m, 1H, H_3′_, dia 1), 4.26–4.23 (m, 1H, H_3′_, dia 2), 4.20–4.18 (m, 1H, H_4′_, dia 2), 4.18–4.16 (m, 1H, H_4′_, dia 1), 3.93 (dd, *J* = 3.7, 1.5 Hz, 1H, H_5′_, dia 1), 3.89 (dd, *J* = 3.7, 1.5 Hz, 1H, H_5′_, dia 2), 3.80 (d, *J* = 2.8 Hz, 1H, H_5′_, dia 2), 3.76 (d, *J* = 2.8 Hz, 1H, H_5′_, dia 1), 3.51 (s, 3H, H_7_ or H_8_, dia 1 or dia 2), 3.47 (s, 3H, H_7_ or H_8_, dia 1 or dia 2), 3.42 (s, 3H, H_7_ or H_8_, dia 1 or dia 2), 3.39 (s, 3H, H_7_ or H_8_, dia 1 or dia 2), 0.95 (s, 9H, H_7′_, dia 1 or dia 2), 0.94 (s, 9H, H_7′_, dia 1 or dia 2), 0.89 (s, 9H, H_7′_, dia 1 or dia 2), 0.88 (s, 9H, H_7′_, dia 1 or dia 2), 0.86 (s, 9H, H_7′_, dia 1 or dia 2), 0.85 (s, 9H, H_7′_, dia 1 or dia 2), 0.13 (s, 3H, H_6′_, dia 1 or dia 2), 0.12 (s, 3H, H_6′_, dia 1 or dia 2), 0.11 (s, 3H, H_6′_, dia 1 or dia 2), 0.11 (s, 3H, H_6′_, dia 1 or dia 2), 0.10 (s, 3H, H_6′_, dia 1 or dia 2), 0.09 (s, 3H, H_6′_, dia 1 or dia 2), 0.08 (s, 3H, H_6′_, dia 1 or dia 2), 0.08 (s, 3H, H_6′_, dia 1 or dia 2), 0.00 (s, 3H, H_6′_, dia 1 or dia 2), 0.00 (s, 3H, H_6′_, dia 1 or dia 2), −0.15 (s, 3H, H_6′_, dia 1 or dia 2), −0.17 (s, 3H, H_6′_, dia 1 or dia 2). ^13^C NMR (75 MHz, CDCl_3_) δ 155.8 (C_1_, dia 1 or dia 2), 155.8 (C_1_, dia 1 or dia 2), 149.9 (C_5_, dia 1 or dia 2), 149.8 (C_5_, dia 1 or dia 2), 144.6 (C_9_, dia 1 or dia 2), 144.5(C_9_, dia 1 or dia 2), 138.8 (C_3_, dia 1 or dia 2), 138.7 (C_3_, dia 1 or dia 2), 135.1 (C_12_, dia 1 or dia 2), 135.1 (C_12_, dia 1 or dia 2), 131.8 (C_14,16_ or C_13,17_, dia 1 or dia 2), 131.8 (C_14,16_ or C_13,17_, dia 1 or dia 2),130.5 (C_14,16_ or C_13,17_, dia 1 or dia 2), 130.5, (C_14,16_ or C_13,17_, dia 1 or dia 2), 130.3 (C_2_, dia 1 or dia 2), 130.2 (C_2_, dia 1 or dia 2), 123.0 (C_4_, dia 1 or dia 2), 123.0 (C_4_, dia 1 or dia 2), 122.5 (C_10_, dia 1 or dia 2), 122.5 (C_10_, dia 1 or dia 2), 122.0 (C_15_, dia 1 or dia 2), 122.0 (C_15_, dia 1 or dia 2), 119.8 (CN, dia 1 or dia 2), 119.8 (CN, dia 1 or dia 2), 103.2 (C_6_, dia 1 or dia 2), 103.2 (C_6_, dia 1 or dia 2), 92.9 (C_1′_, dia 1 or dia 2), 92.9 (C_1′_, dia 1 or dia 2), 86.9 (C_4′_, dia 1 or dia 2), 86.6 (C_4′_, dia 1 or dia 2), 77.5 (C_2′_, dia 1 or dia 2), 77.3 (C_2′_, dia 1 or dia 2), 72.6 (C_3′_, dia 1 or dia 2), 72.4 (C_3′_, dia 1 or dia 2), 62.9 (C_5′_, dia 1 or dia 2), 62.8 (C_5′_, dia 1 or dia 2), 56.4 (C_7_ or C_8_, dia 1 or dia 2), 56.4 (C_7_ or C_8_, dia 1 or dia 2), 56.0 (C_7_ or C_8_, dia 1 or dia 2), 56.0 (C_7_ or C_8_, dia 1 or dia 2), 41.0 (C_11_, dia 1 or dia 2), 41.0 (C_11_, dia 1 or dia 2), 26.1 (3C, C_7′_, dia 1 or dia 2), 26.1 (3C, C_7′_, dia 1 or dia 2), 25.9 (2C, C_7′_, dia 1 or dia 2), 25.8 (2C, C_7′_, dia 1 or dia 2), 18.5 (C_8′_, dia 1 or dia 2), 18.5 (C_8′_, dia 1 or dia 2), 18.2 (C_8′_, dia 1 or dia 2), 18.2 (C_8′_, dia 1 or dia 2),18.1 (C_8′_, dia 1 or dia 2), 18.1 (C_8′_, dia 1 or dia 2), −4.3 (2C, C_6′_, dia 1 or dia 2), −4.3 (2C, C_6′_, dia 1 or dia 2), −4.4 (C_6′_, dia 1 or dia 2), −4.4 (C_6′_, dia 1 or dia 2), −4.5 (C_6′_, dia 1 or dia 2), −4.5 (C_6′_, dia 1 or dia 2), −4.5 (C_6′_, dia 1 or dia 2), −4.5 (C_6′_, dia 1 or dia 2), −5.0 (C_6′_, dia 1 or dia 2), −5.0 (C_6′_, dia 1 or dia 2), −5.0 (C_6′_, dia 1 or dia 2), −5.0 (C_6′_, dia 1 or dia 2). IR (cm^−1^): 2930, 2857, 2360, 2341, 2255, 1596, 1256, 1109, 1043, 836, 735. HRMS (ESI) *m*/*z*: [M + H]^+^ Calcd for C_41_H_66_BrN_5_O_6_Si_3_H 888.3577. Found 888.3576.

#### 3.4.8. Synthesis of 4-(1-((2*R*,3*R*,4*S*,5*R*)-3,4-dihydroxy-5-(hydroxymethyl)tetrahydrofuran-2-yl)-1*H*-1,2,3-triazol-4-yl)-8-phenyl-1,6-naphthyridin-7(6*H*)-one hydrochloride Salt TzNat **A**

A 37% *w*/*w* aqueous solution of hydrochloric acid (3 equiv.) was added to a stirred solution of compound **15** (1.25 g, 1.54 mmol) in THF (0.1 M). The solution was stirred at room temperature for 24 h. The mixture was tritured in Et_2_O and filtered to yield the TzNat A as a hydrochloride salt. Red powder, M = 695 mg. Yield: 98%. ^1^H NMR (400 MHz, CH_3_OD) δ 10.34 (s, 1H, H_6_), 9.36 (s, 1H, H_16_), 8.77 (d, *J* = 5,8 Hz, 1H, H_1_), 7.88 (d, *J* = 5.7, 1H, H_2_), 7.70–7.45 (m, 5H, H_10/11/12/13/14_), 6.23 (d, *J* = 3.7 Hz, 1H, H_1′_), 4.65 (dd, *J* = 4.3, 4.3 Hz, 1H, H_2′_), 4.40 (t, *J* = 5.1 Hz, 1H, H_3′_), 4.23 (dt, *J* = 4.1, 4.0 Hz 1H, H_4′_), 3.91 (dd, *J* = 12.3, 3.1 Hz, 1H, H_5′_), 3.78 (dd, *J* = 12.3, 3.1 Hz, 1H, H_5′_). ^13^C NMR (101 MHz, DMSO) δ 160.9 (C_7_), 155.0 (C_1_), 147.9 (C_6_), 143.6 (C_3,_ C_15_), 134.2 (C_8,9_), 131.9 (C_10,14_), 128.0 (C_11,13_) 127.4 (C_12_), 125.6 (C_16_), 117.4 (C_2_), 114.2 (C_4,5_), 93.1 (C_1′_), 86.6 (C_4′_), 75.6 (C_2′_), 70.7 (C_3′_), 61.6 (C_5′_). IR (cm^−1^): 3349 (O-H), 2963, 2924, 2360, 2341, 1631, 1589, 1263, 1101, 814. HRMS (ESI) *m*/*z*: [M + H]^+^ Calcd for C_21_H_19_N_5_O_5_H 422.1459. Found 422.1458.

#### 3.4.9. Synthesis of 4-(1-((2*R*,3*R*,4*S*,5*R*)-3,4-dihydroxy-5-(hydroxymethyl)tetrahydrofuran-2-yl)-1*H*-1,2,3-triazol-4-yl)-8-(4-methoxyphenyl)-1,6-naphthyridin-7(6*H*)-one hydrochloride salt TzNat **B**

A 37% *w*/*w* aqueous solution of hydrochloric acid (3 equiv.) was added to a stirred solution of compound **16** (68 mg, 0.08 mmol) in THF (0.1 M). The solution was stirred at room temperature for 24 h. The mixture was tritured in Et_2_O and filtered to yield the TzNat **B** as a hydrochloride salt. Red powder, M = 30 mg. Yield: 82%.^1^H NMR (400 MHz, CH_3_OD) δ 10.32 (s, 1H, H_6_), 9.36 (s, 1H, H_16_), 8.77 (d, *J* = 6.0 Hz, 1H, H_1_), 7.88 (d, *J* = 5.9 Hz, 1H, H_2_), 7.47–7.40 (m, 2H, H_10,14_ or H_11,12_), 7.21–7.14 (m, 2H, H_10,14_ or H_11,12_), 6.23 (d, *J* = 3.7 Hz, 1H, H_1′_), 4.65 (dd, *J* = 4.9, 3.7 Hz, 1H, H_2′_), 4.40 (t, *J* = 5.1 Hz, 1H, H_3′_), 4.23 (dt, *J* = 5.2, 3.5 Hz, 1H, H_4′_), 3.91 (s, 3H, H_17_), 3.90 (dd, *J* = 12.3, 3.9 Hz, 1H, H_5′_), 3.78 (dd, *J* = 12.3, 3.9 Hz, 1H, H_5′_). ^13^C NMR (101 MHz, DMSO) δ 160.6 (C_7_), 158.5 (C_12,8_), 154.0 (C_1_), 147.9 (C_3_), 144.1 (C_6_), 143.0 (C_15_), 132.6 (C_10,14_ or C_11,13_), 126.0 (C_16_), 125.1 (C_9_), 116.6 (C_2′_), 113.7 (C_4,5_), 113.3 (C_10,14_ or C_11,13_), 92.6 (C_1′_), 86.2 (C_4′_), 75.2 (C_2′_), 70.2 (C_3′_), 61.1 (C_5′_), 55.1 (C_17_). IR (cm^−1^): 3348, 2880, 2857, 2360, 2341, 1630, 1587, 1248, 1179, 1101, 1049, 824. HRMS (ESI) *m*/*z*: [M + H]^+^ Calcd for C_22_H_21_N_5_O_6_H 452.1565. Found 452.1563.

#### 3.4.10. 8-(4-bromophenyl)-4-(1-((2R,3R,4S,5R)-3,4-dihydroxy-5-(hydroxymethyl)tetrahydrofuran-2-yl)-1H-1,2,3-triazol-4-yl)-1,6-naphthyridin-7(6H)-one hydrochloride Salt TzNat C

A 37% *w*/*w* aqueous solution of hydrochloric acid (3 equiv.) was added to a stirred solution of compound **17** (86 mg, 0.09 mmol) in THF (0.1 M). The solution was stirred at room temperature for 24 h. The mixture was tritured in Et_2_O and filtered to yield the Tz Nat C as a hydrochloride salt. Orange-red powder, M = 37 mg. Yield: 77%. ^1^H NMR (400 MHz, CH_3_OD) δ 10.37 (s, 1H, H_6_), 9.36 (s, 1H H_16_), 8.78 (d, *J* = 6.0 Hz, 1H, H_1_), 7.89 (d, *J* = 6.0 Hz, 1H, H_2_), 7.82–7.76 (m, 2H, H_10,14_ or H_11,12_), 7.47–7.41 (m, 2H, H_10,14_ or H_11,12_), 6.22 (d, *J* = 3.7 Hz, 1H, H_1′_), 4.65 (dd, *J* = 4.9, 3.7 Hz, 1H, H_2′_), 4.40 (t, *J* = 5.1 Hz, 1H, H_3′_), 4.22 (dt, *J* = 5.2, 3.4 Hz, 1H, H_4′_), 3.90 (dd, *J* = 12.3, 3.1 Hz, 1H, H_5′_), 3.78 (dd, *J* = 12.3, 3.9 Hz, 1H, H_5′_). ^13^C NMR (101 MHz, DMSO) δ 160.2 (C_7_), 154.9 (C_1_), 152.7 (C_4_), 149.6 (C_5_), 147.3 (C_6_), 143.2 (C_15_), 141.2 (C_3_), 133.7 (C_10,14_ or C_11,13_), 133.2 (C_9_), 130.4 (C_10,14_ or C_11,13_), 125.4 (C_16_), 120.2 (C_12_), 116.9 (C_2_), 113.5 (C_8_), 92.5 (C_1′_), 86.6 (C_4′_), 75.1 (C_2′_), 70.2 (C_3′_), 61.1 (C_5′_). IR (cm^−1^): 3304, 3085, 2980, 2888, 2360, 2341, 1641, 1588, 1261, 1101, 1073, 678. HRMS (ESI) *m*/*z*: [M + H]^+^ Calcd for C_21_H_18_BrN_5_O_5_H 500.0564. Found 500.0561.

## 4. Conclusions

We have synthetized, using a Cu alkyne-azide cycloaddition (CuAAC) reaction, three original molecules as potential fluorescent ribonucleoside analogues, incorporating 1,6-naphthyridin-7(6*H*)-one as the fluorescent nucleobase and a 1,2,3-triazole as a linkage to a ribofuranosyl. Optical properties of these molecules have been studied in solvents of different polarity. Importantly, these molecules are fluorescent, showing a dual emission in highly polar aprotic solvent. In addition, a remarkable change of emissivity depending on the polarity of the solvent has been observed. We posit that these optical properties are useful in developing these molecules as flexible fluorescent probes for studying binding sites of enzymes and protein–nucleic acid interaction. In addition, such analogues could be of great interest in the search for new antiviral or antitumoral drugs based on nucleosides.

## Figures and Tables

**Table 1:** UV–Vis Absorption and Fluorescence Emission Properties of Compounds TzNat **A**–**C** in various solvents.

	UV–Vis ^[b]^		Fluorescence ^[c]^			
Compd	Solvent ^[a]^	λ_abs_ (ε)	λ_em_	Stokes ^[d]^ Shifts in cm^−1^	Φ_F_ ^[e]^	
TzNat **A**	CHCl_3_ CH_2_Cl_2_ THF acetone CH_3_CN DMF DMSO EtOH H_2_O PBS	358 (8.8), 479 (5.8) 356 (10.3), 476 (4.7) 369 (6.4), 479 (0.6) 363 (5.3), 474 (0.5) 360 (5.1), 465 (1.9) 369 (5.2), 480 (1.1) 374 (3.6), 483 (1.1) 374 (3.8), 451(3.8) 427 (4.9) 427 (6.6)	471 474 461 460 458 413, 478, 560 421, 475, 560 477 563 558	6701 6992 5809 5943 6179 6179 5685 5773 5657 5498	<0.01 <0.01 0.32 0.20 0.11 0.01 <0.01 <0.01 <0.01 <0.01	
TzNat **B**	CHCl_3_ CH_2_Cl_2_ THF acetone CH_3_CN DMF DMSO EtOH H_2_O PBS	317 (17.2), 461 (6.7) 325 (16.3), 498 (4.9) 378 (7.6), 490 (0.4) 369 (8.1), 488 (1.6) 369 (7.6), 468 (3.1) 379 (9.5), 486 (2.2) 380 (7.1), 490 (1.9) 386 (3.2), 457 (4.0) 433 (8.1) 431 (7.3)	492 499 489 492 494 499 502 -- -- --	11,220 10,729 6500 6775 6867 6345 6395 -- -- --	<0.01 <0.01 0.34 0.22 0.18 0.14 0.12 -- -- --	
TzNat **C**	CHCl_3_ CH_2_Cl_2_ THF acetone CH_3_CN DMF DMSO EtOH H_2_O PBS	356 (12.5), 461 (6.4) 356 (9.8), 454 (6.1) 369 (8.6), 479 (1.1) 363 (8.4), 470 (1.7) 363 (5.4), 470 (2.1) 372 (9.2), 470 (1.7) 371 (9.2), 472 (2.7) 378 (4.6), 452 (5.5) 427 (7.5) 429 (7.1)	470 467 460 459 458 476, 556 477, 558 479 561 561	6813 6676 5361 5761 5714 5873 5989 5578 5593 5484	<0.01 <0.01 0.31 0.25 0.11 <0.01 <0.01 <0.01 <0.01 <0.01	

^[a]^. All TzNat have been pre-solubilized in DMSO at 10^−3^ M and used it as stock solution to prepare 10^−5^ M diluted solutions in solvents. ^[b]^. Absorption maxima measured in solution (10^−5^ M) at room temperature in nm (extinction coefficient at absorption maxima in 10^3^ M^−1^ cm^−1^). ^[c]^. Fluorescence wavelengths measured at room temperature at a concentration of 10^−5^ M after excitation at the maximum absorption wavelength. ^[d]^. Stokes shift in cm^−1^ defined as the wavelength difference between the absorption and emission peak maxima. ^[e]^. Absolute fluorescence quantum yields were measured.

## Data Availability

All data generated or analyzed during this study are included in this published article and its Supplementary Information Files.

## Supplementary Materials

The following supporting information can be downloaded at: https://www.mdpi.com/article/10.3390/molecules29030687/s1.
