# Supplementary material for: 1H-1,2,3-triazolyl-1,6-naphthyridin-7(6H)-ones as Potential Fluorescent Nucleoside Analogues: Synthesis and Optical Properties
Source: Molecules. 2024 Feb 1;29(3):687. doi: 10.3390/molecules29030687 (PMC10856630; doi:10.3390/molecules29030687)

Supporting information

1H-1,2,3-triazolyl-1,6-naphthyridin-7(6*H*)-ones as potential fluorescent nucleoside analogues : Synthesis and optical properties

Anissa Beghennou, Océane Rondot, Vincent Corcé and Candice Botuha

I. Table S1. CuAAc reaction attempts to get Compound 4

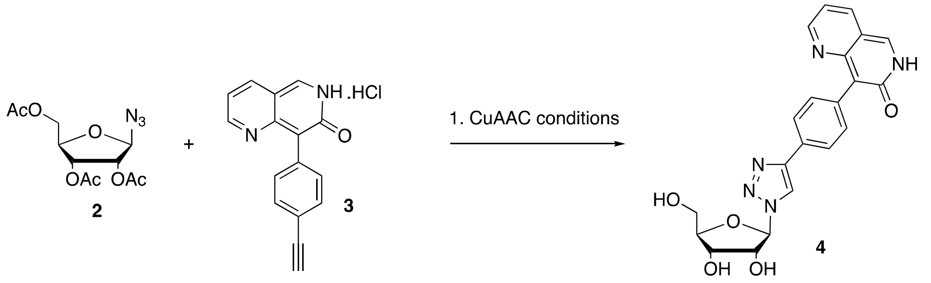

a. Tris(benzyltriazolylmethyl)amine. b. Diisopropylethylamine. c. Solubility issues

Conditions	Solvent	Temp	Time	Product/outcome
5% mol. $CuSO_4.5H_2O$, 10% mol. NaAsc	*t*-BuOH/ Phosphate buffer	20 °C	24h	No reaction
10% mol. $CuSO_4.5H_2O$, 30% mol. NaAsc, 10% mol. TBTA[a]	*t*-BuOH/ H_2O	75 °C	24h	No reaction
10% mol. $CuSO_4.5H_2O$, 30% mol. NaAsc	DMF	150 °C (MW)	30min.	Degradation
10% mol. $CuSO_4.5H_2O$, 30% mol. NaAsc, 1 equiv. DIPEA	CH_3CN	60 °C	24h	No reaction
2 equiv.. $CuSO_4.5H_2O$, 4 equiv. NaAsc, 1 equiv. DIPEA	CH_3CN/ H_2O	20 °C	24h	No reaction[c]
10% mol. CuI, 1 equiv. DIPEA	CH_3CN	20 °C	24h	No reaction
10% mol. Cu(IMes)Cl, 1 equiv. DIPEA	CH_3CN	20 °C	24h	No reaction
10% mol. Cu(IMes)Cl, 1 equiv. DIPEA	MeOH	20 °C	24h	No reaction
2 equiv. CuI, 1 equiv. DIPEA	THF	60 °C	24h	No reaction[c]

II. Absorption spectra in various solvent of TzNat A-C

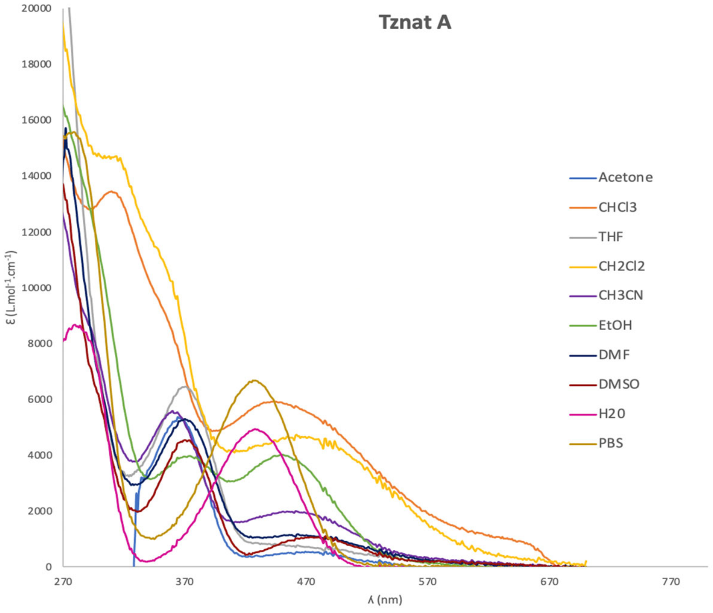

Figure S1

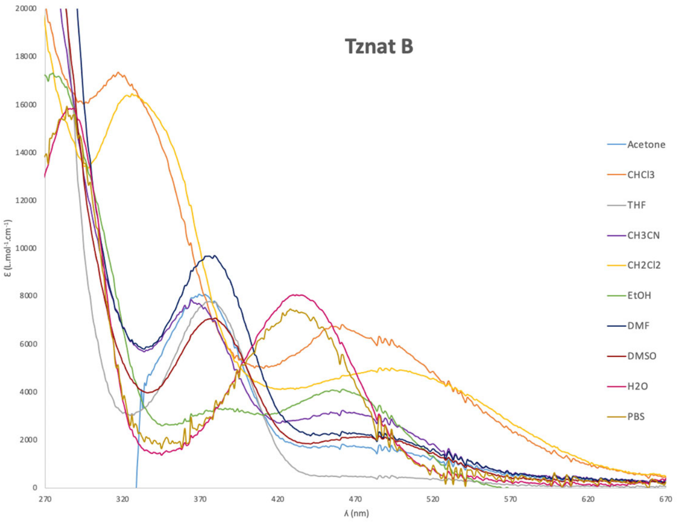

Figure S2

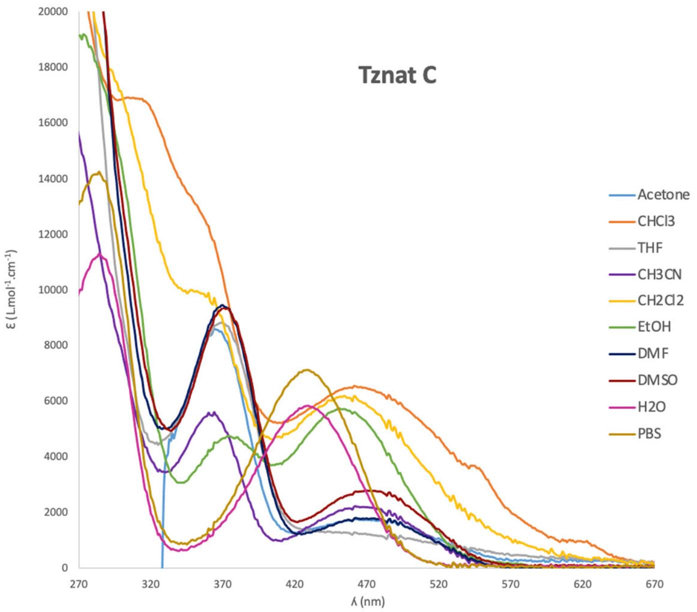
Tznat C
20000
18000
16000
14000
12000
10000
8000
6000
4000
2000
0
270
320
370
420
470
520
570
620
670
λ (nm)
ε (L.mol-1.cm-1)
Acetone
CHCl3
THF
CH3CN
CH2Cl2
EtOH
DMF
DMSO
H2O
PBS

Figure S3

III. ^{1}H and ^{13}C NMR spectra

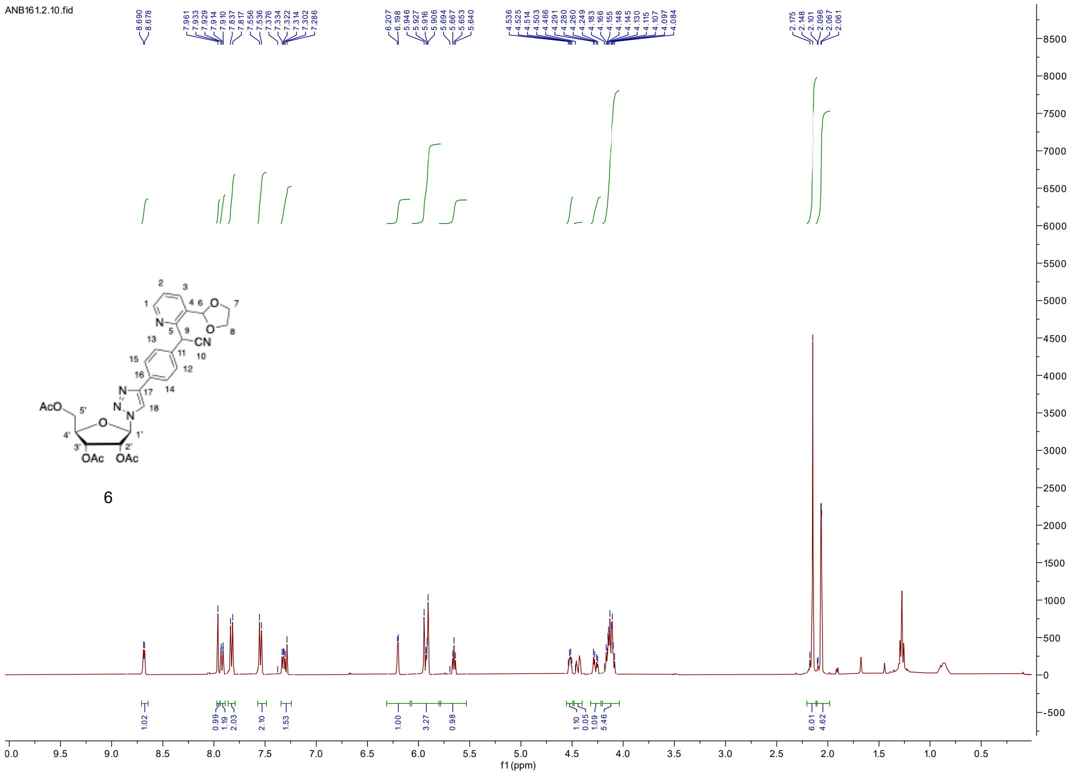

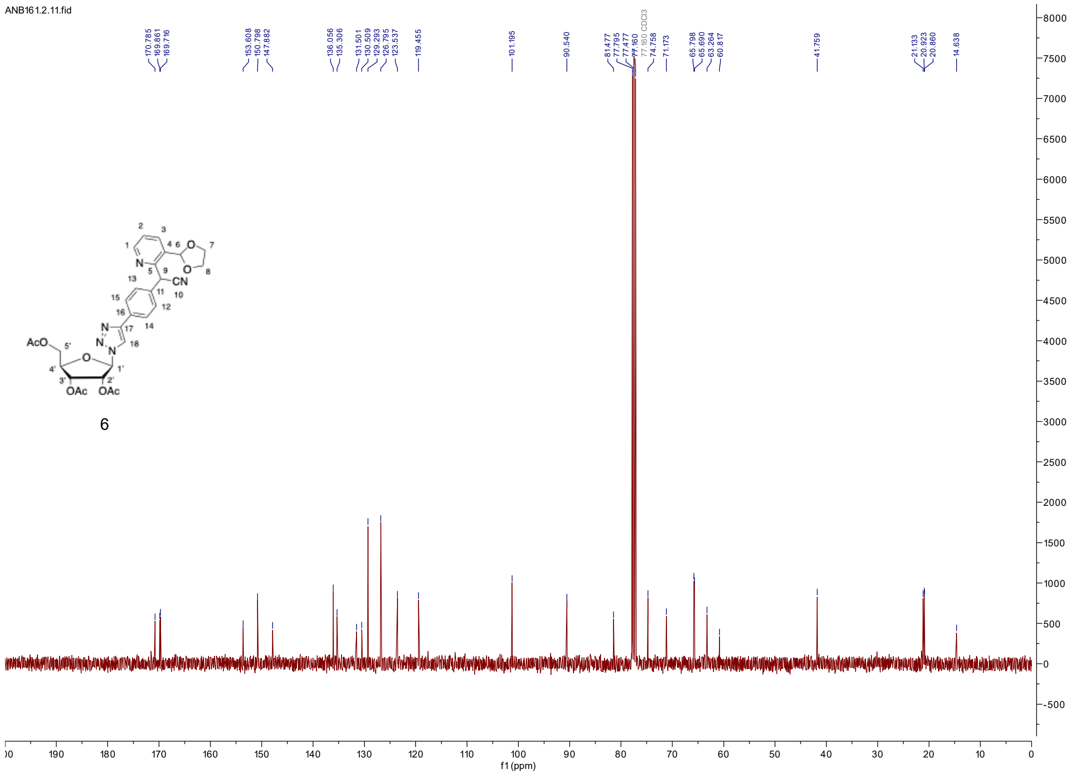

bc194p.10.fid

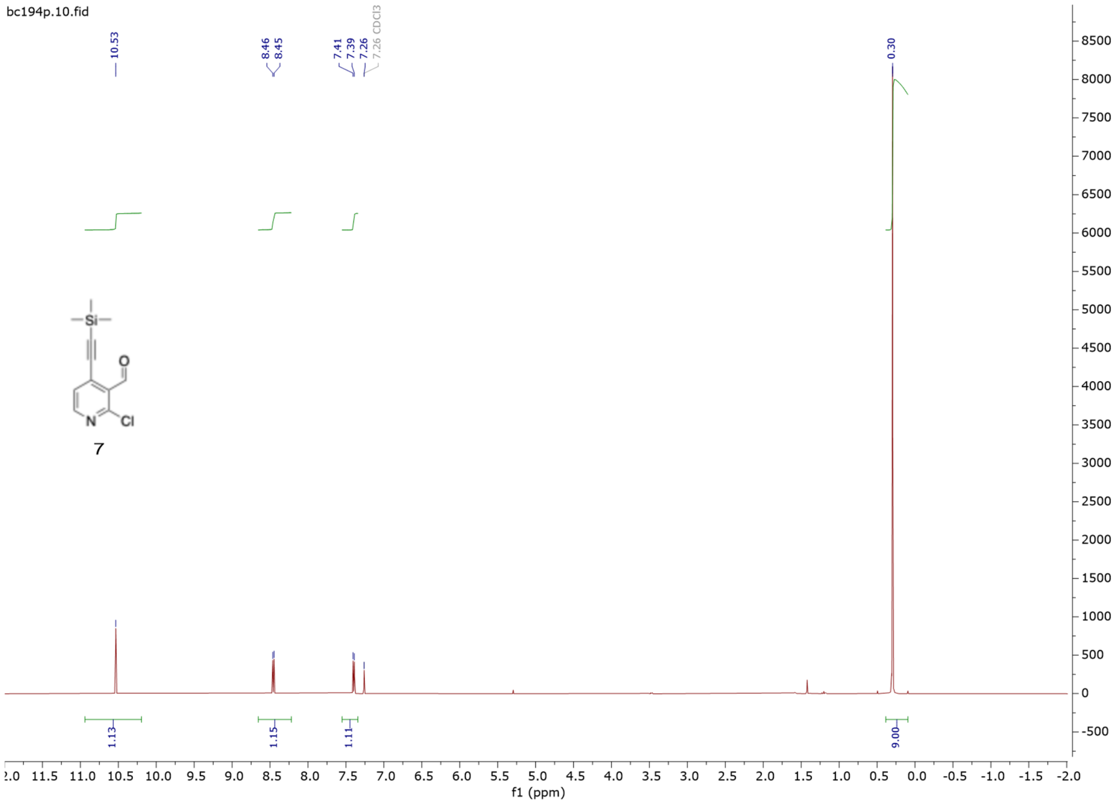

ANB-BC192.11.fid

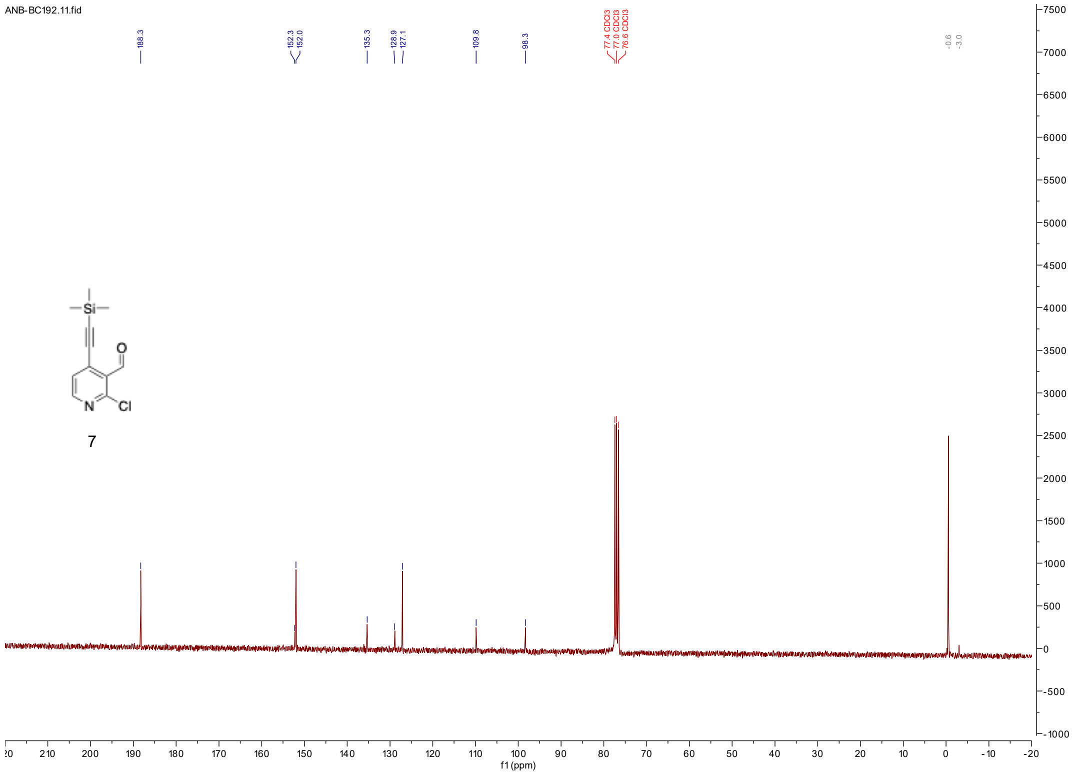

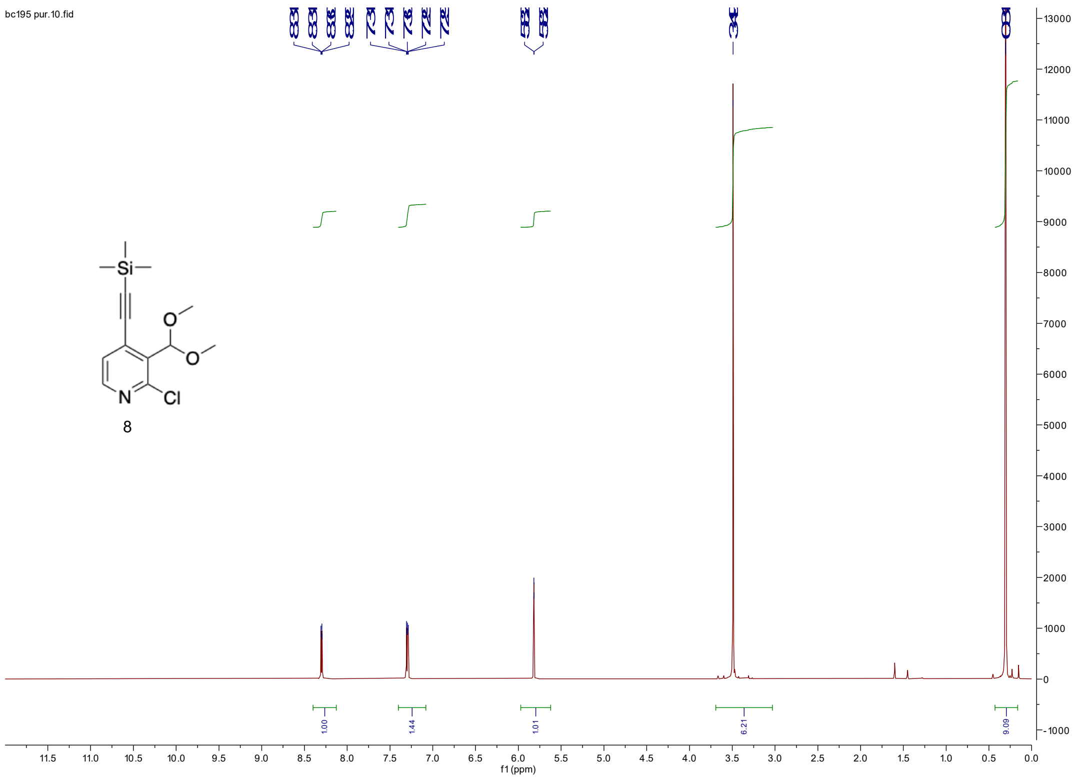
bc195 pur.10.fid
Si
O
O
N
Cl
8
1.00
1.44
1.01
6.21
9.09
f1 (ppm)

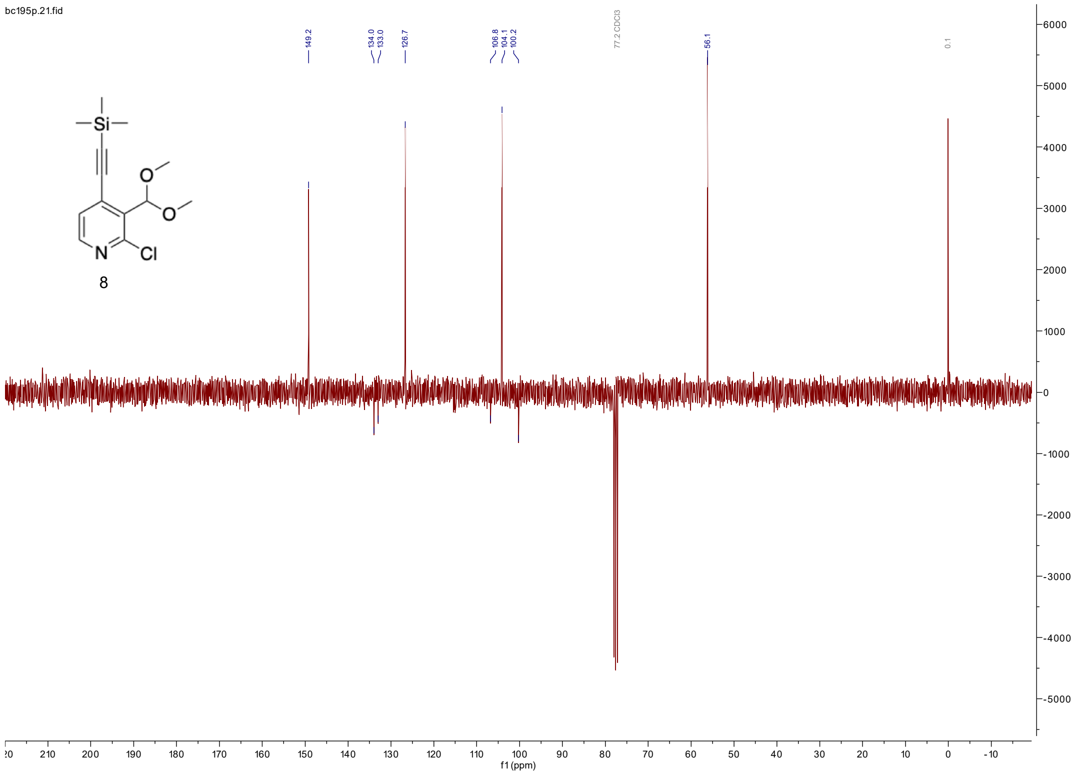
bc195p.21.fid
149.2
134.0
133.0
126.7
106.8
104.1
100.2
77.2 CDCl3
56.1
0.1
Si
O
O
N
Cl
8
f1 (ppm)

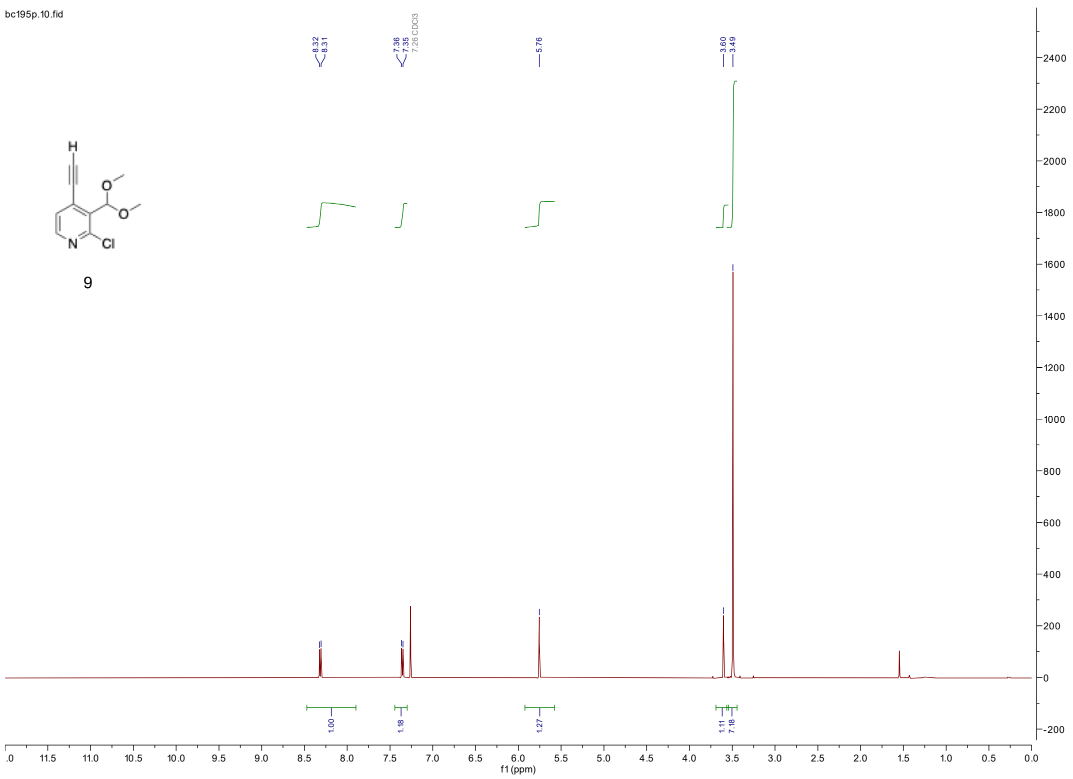
bc195p.10.fid
8.32
8.31
7.36
7.35
7.26 CDCl3
5.76
3.60
3.49
9
1.00
1.18
1.27
1.11
7.18
f1 (ppm)

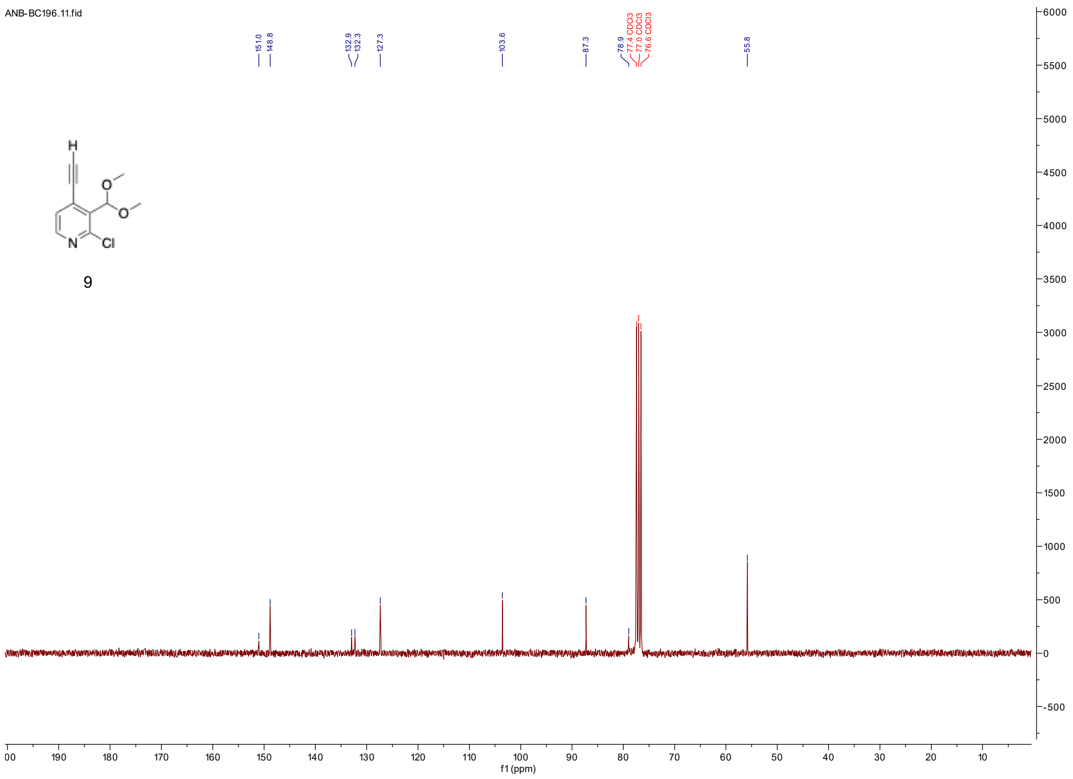
ANB-BC196.11.fid
151.0
148.8
132.9
132.3
127.3
103.6
87.3
78.9
77.4 CDCl3
77.0 CDCl3
76.6 CDCl3
55.8
9
f1 (ppm)

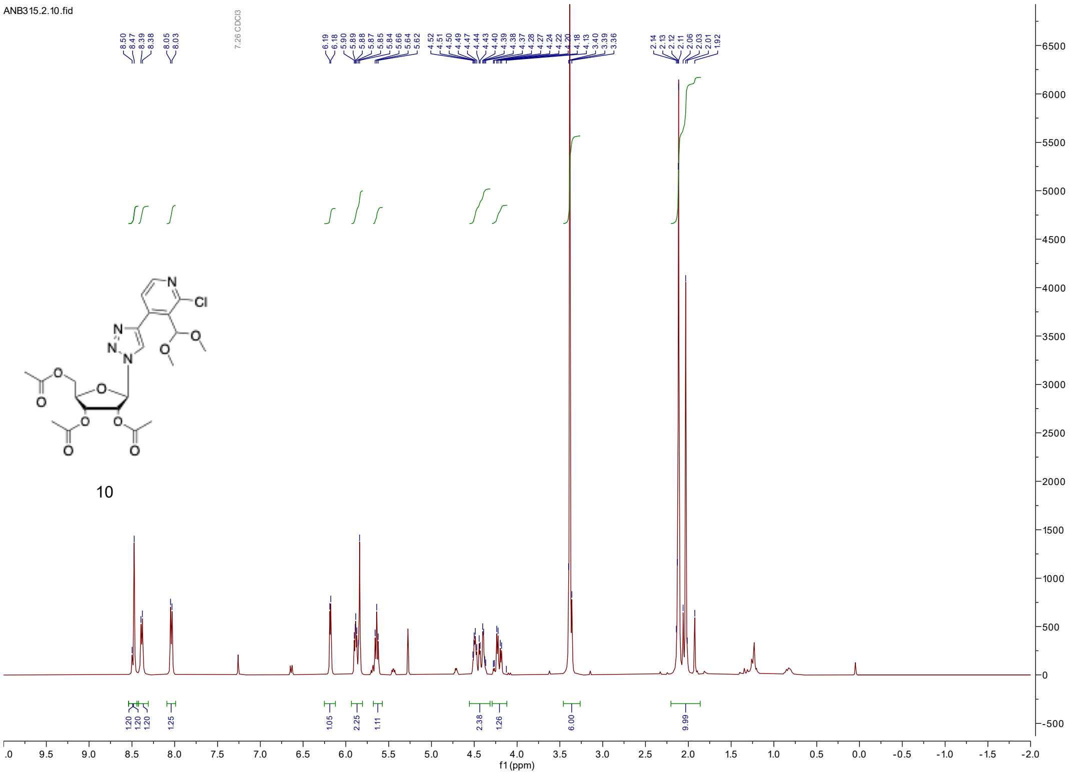
ANB315.2.10.fid
8.50 8.47 8.39 8.38 8.05 8.03
7.26 CDCl3
6.19 6.18 5.90 5.89 5.88 5.87 5.85 5.84 5.66 5.64 5.62
4.52 4.51 4.50 4.49 4.47 4.44 4.43 4.40 4.39 4.38 4.37 4.28 4.27 4.24 4.22 4.20 4.18 4.13 3.40 3.39 3.36
2.14 2.13 2.12 2.11 2.06 2.03 2.01 1.92
1.20 1.20 1.20 1.25 1.05 2.25 1.11 2.38 1.26 6.00 9.99
Cl
N
O
10
f1 (ppm)

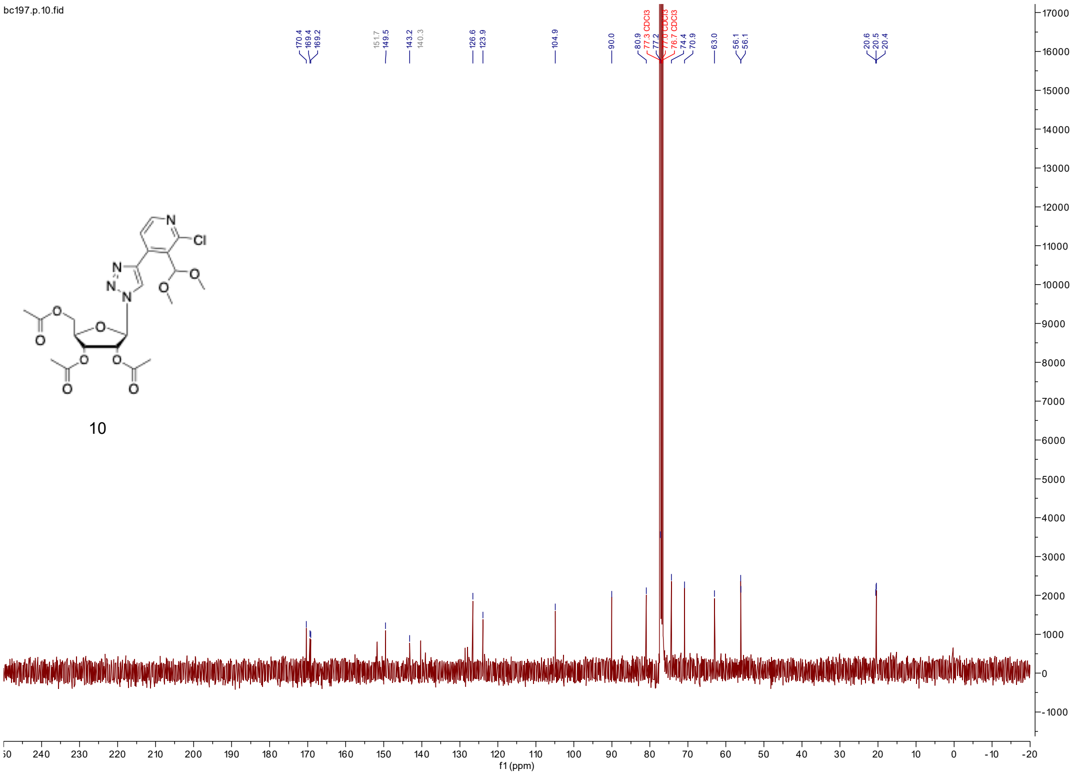
bc197.p.10.fid
170.4 169.4 169.2
151.7 149.5
143.2 140.3
126.6 123.9
104.9
90.0
80.9 77.3 CDCl3 77.2 77.0 CDCl3 76.7 CDCl3 74.4 70.9
63.0
56.1 56.1
20.6 20.5 20.4
Cl
N
O
10
f1 (ppm)

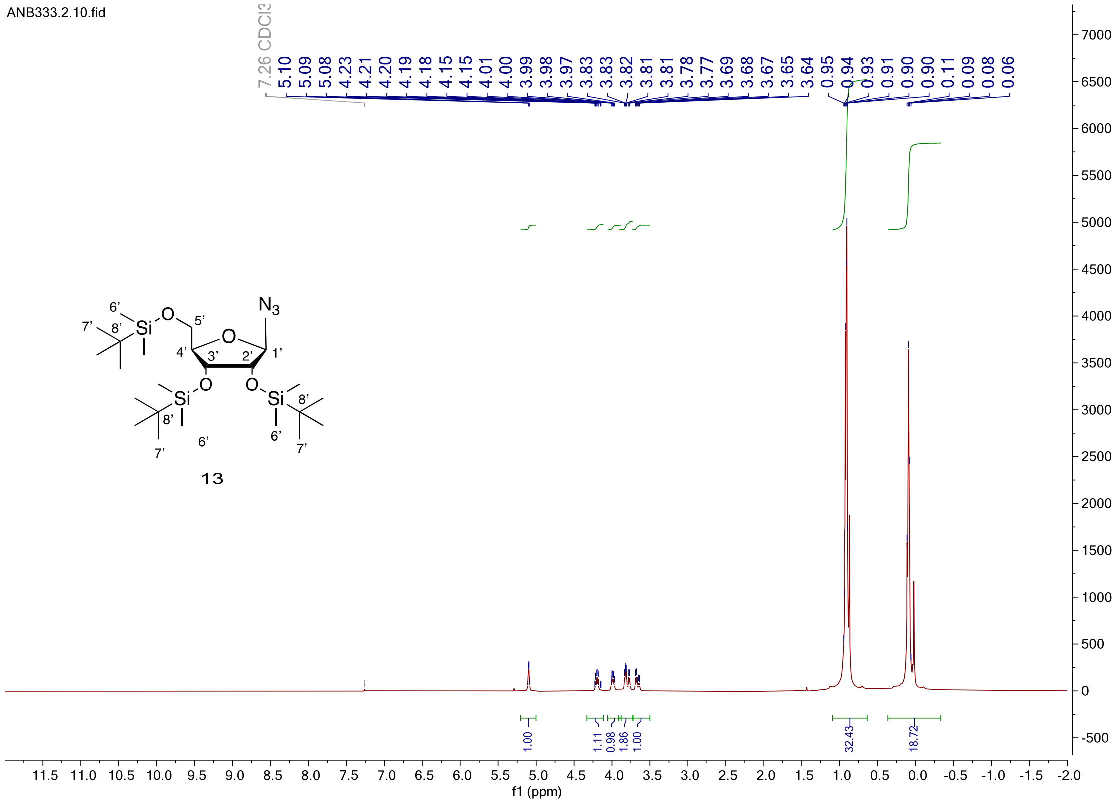
ANB333.2.10.fid
7.26 CDCl3
5.10 5.09 5.08 4.23 4.21 4.20 4.19 4.18 4.15 4.15 4.01 4.00 3.99 3.98 3.97 3.83 3.83 3.82 3.81 3.81 3.78 3.77 3.69 3.68 3.67 3.65 3.64 0.95 0.94 0.93 0.91 0.90 0.90 0.11 0.09 0.08 0.06
1.00
1.11
0.98
1.86
1.00
32.43
18.72
N3
O
Si
6'
7'
8'
5'
4'
3'
2'
1'
13
f1 (ppm)

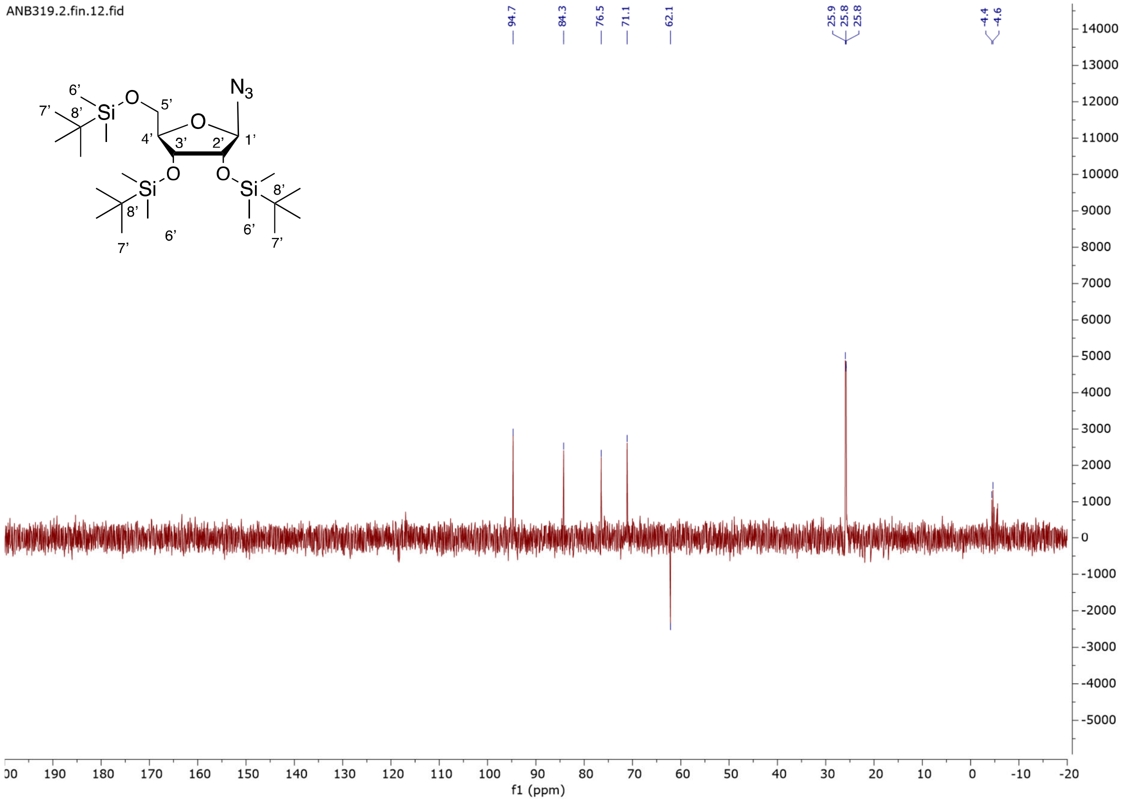
ANB319.2.fin.12.fid
94.7
84.3
76.5
71.1
62.1
25.9
25.8
25.8
-4.4
-4.6
N3
O
Si
6'
7'
8'
5'
4'
3'
2'
1'
f1 (ppm)

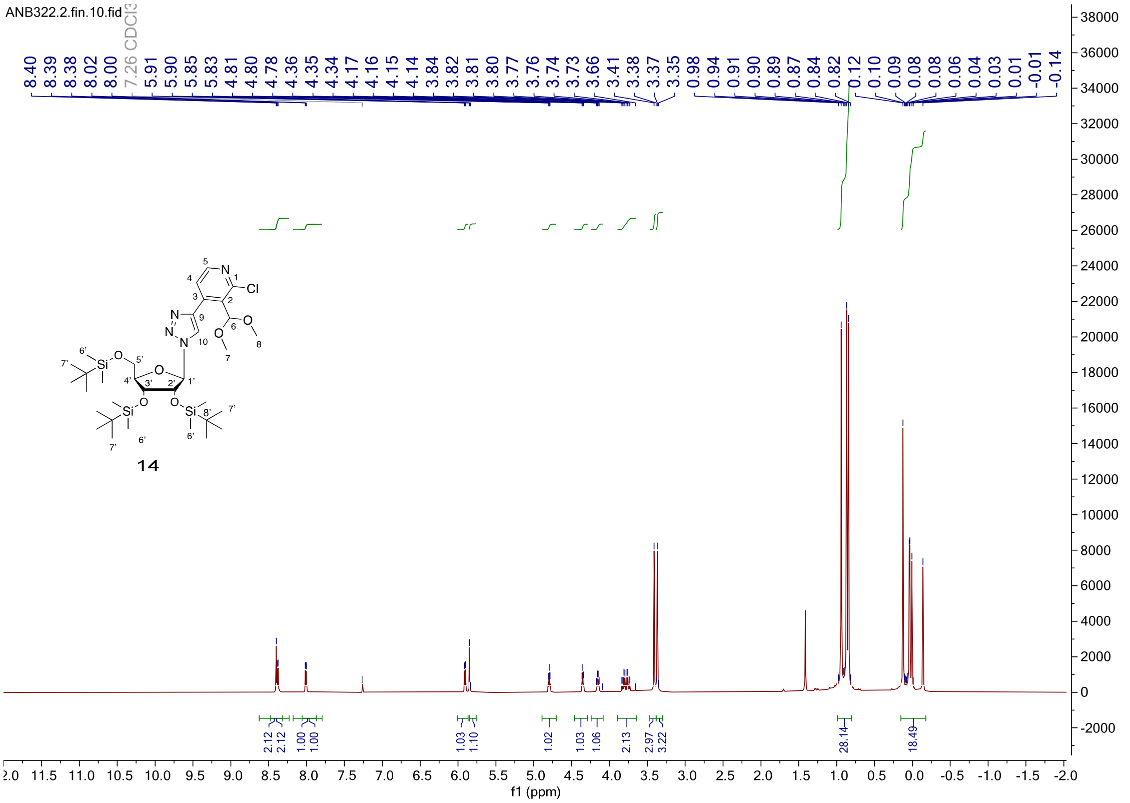
ANB322.2.fin.10.fid
8.40 8.39 8.38 8.02 8.00 7.26 CDCl3 5.91 5.90 5.85 5.83 4.81 4.80 4.78 4.36 4.35 4.34 4.17 4.16 4.15 4.14 3.84 3.82 3.81 3.80 3.77 3.76 3.74 3.73 3.66 3.41 3.38 3.37 3.35 0.98 0.94 0.91 0.90 0.89 0.87 0.84 0.82 0.12 0.10 0.09 0.08 0.08 0.06 0.04 0.03 0.01 -0.01 -0.14
2.12 2.12 1.00 1.00 1.03 1.10 1.02 1.03 1.06 2.13 2.97 3.22 28.14 18.49
14
f1 (ppm)

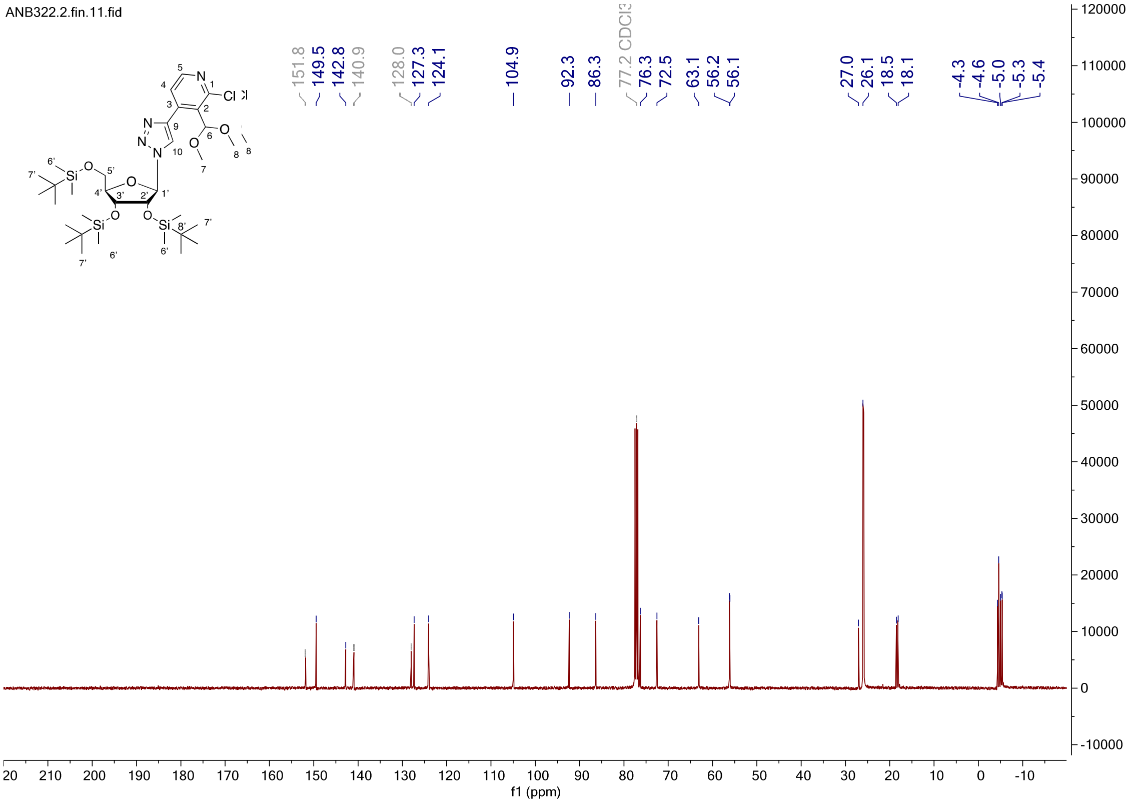
ANB322.2.fin.11.fid
151.8 149.5 142.8 140.9 128.0 127.3 124.1 104.9 92.3 86.3 77.2 CDCl3 76.3 72.5 63.1 56.2 56.1 27.0 26.1 18.5 18.1 -4.3 -4.6 -5.0 -5.3 -5.4
f1 (ppm)

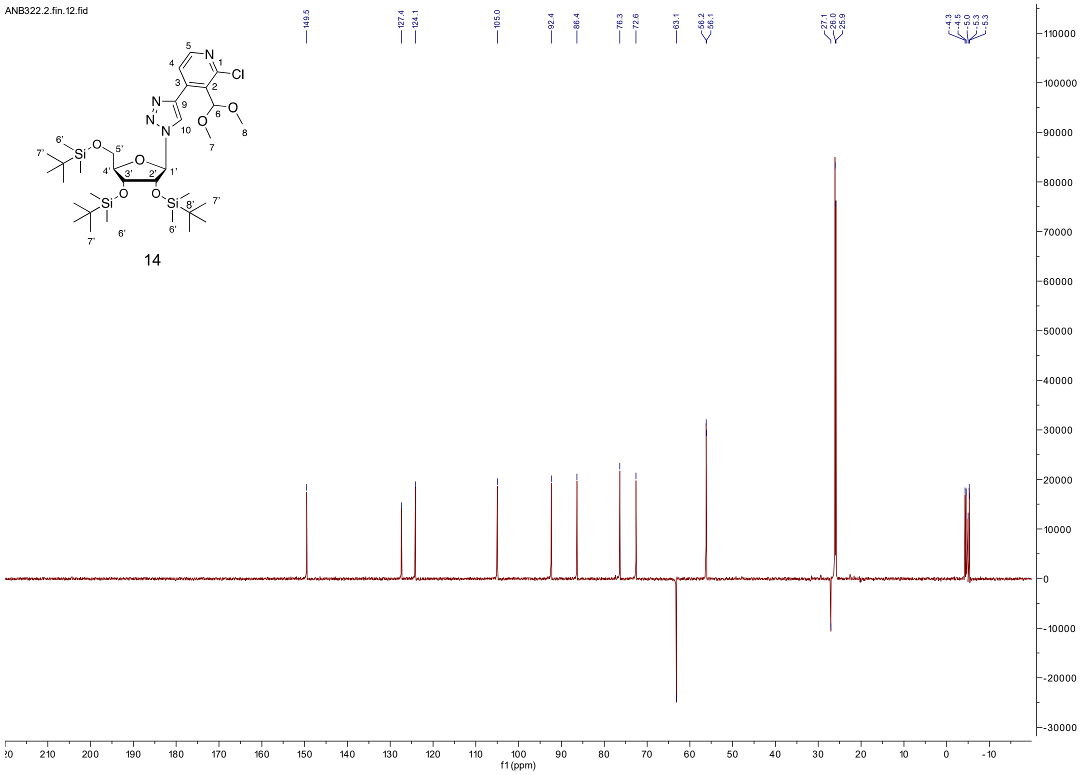

HSQC spectrum of molecule 14 at 400 MHz is shown below

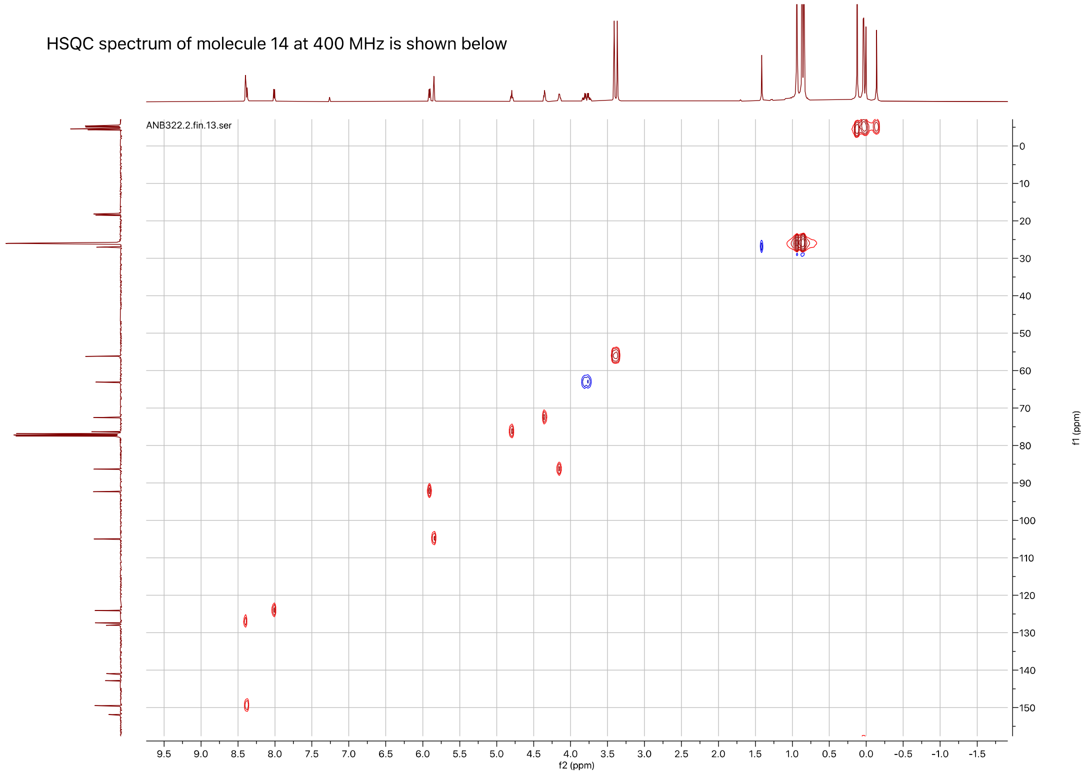

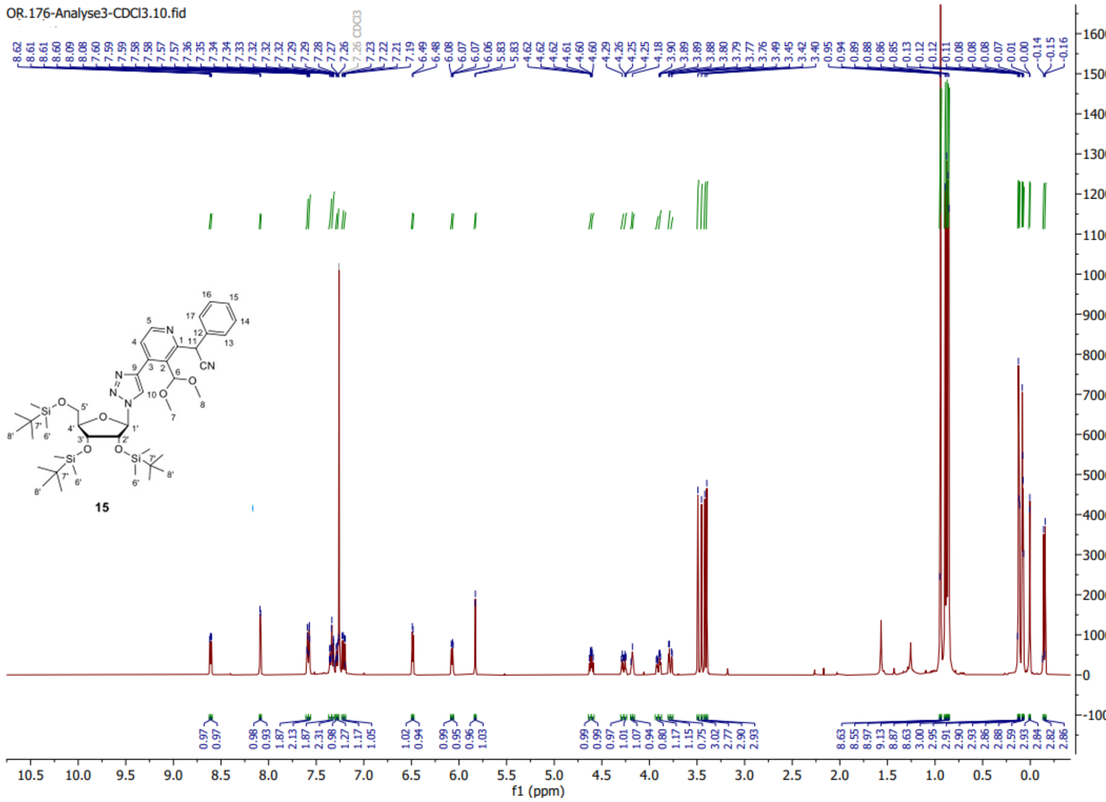

Zoom area 5 – 9 ppm

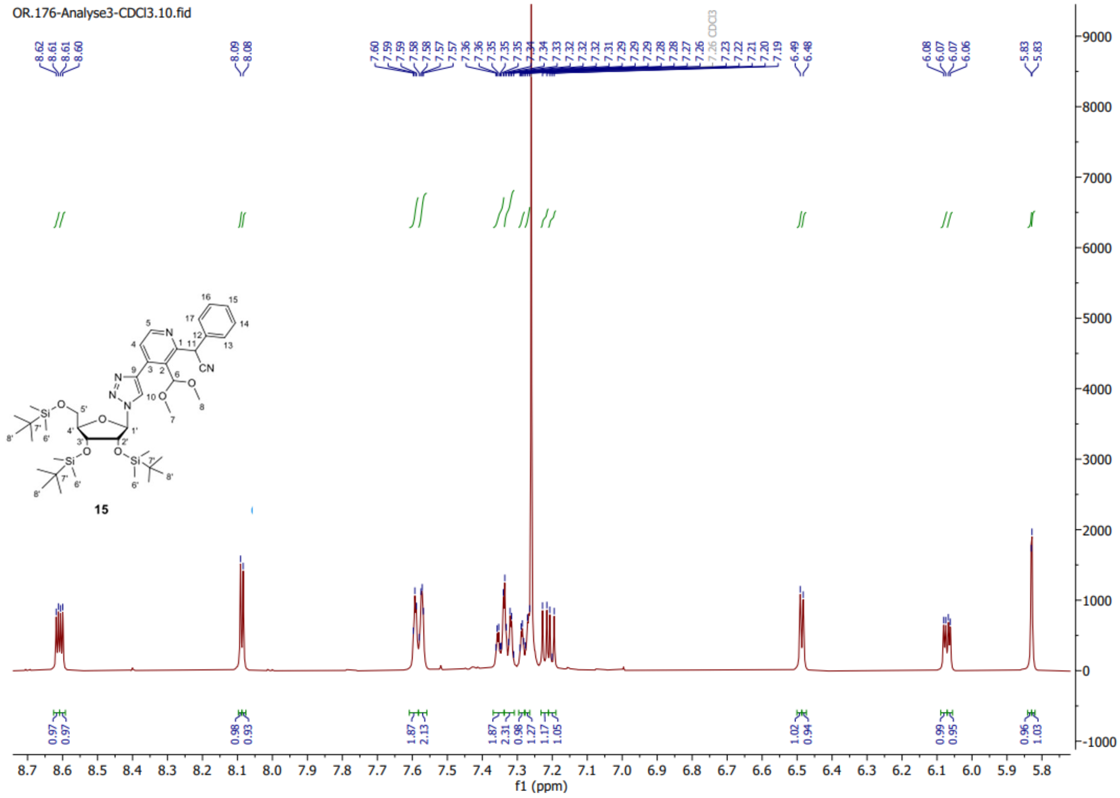

Zoom area 3 – 5 ppm

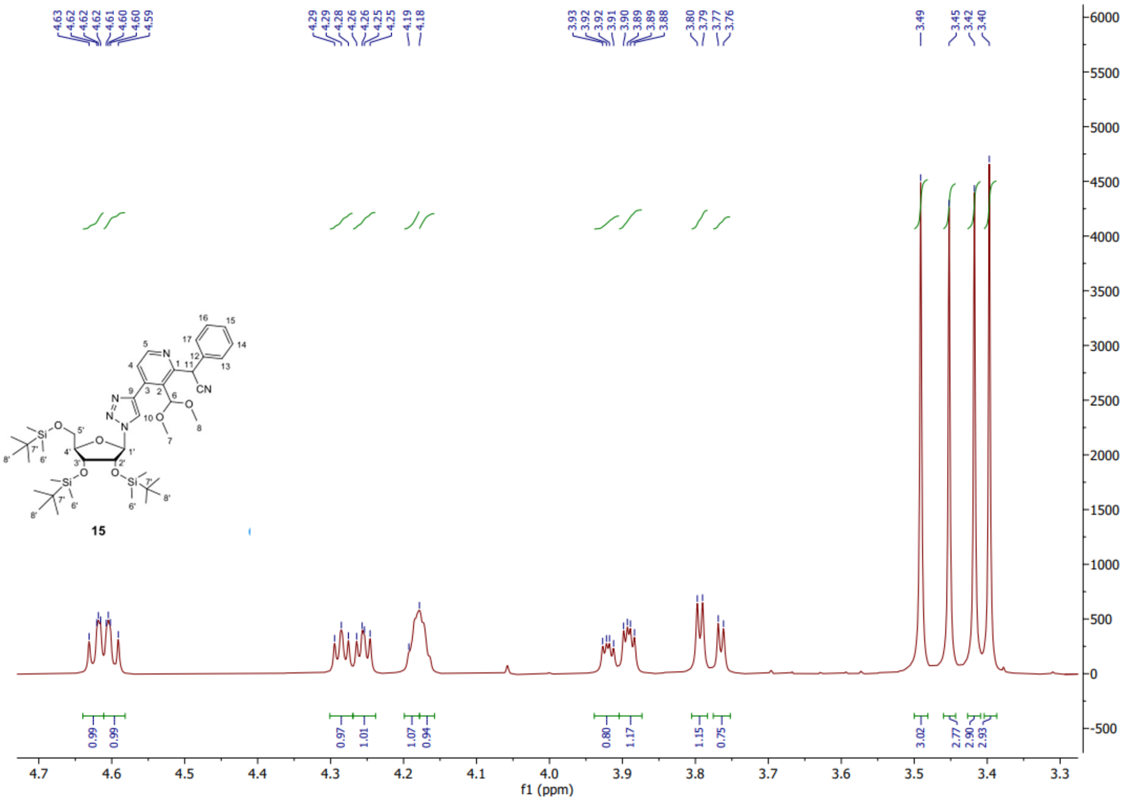

OR.176-Analyse3-CDCl3.11.fid

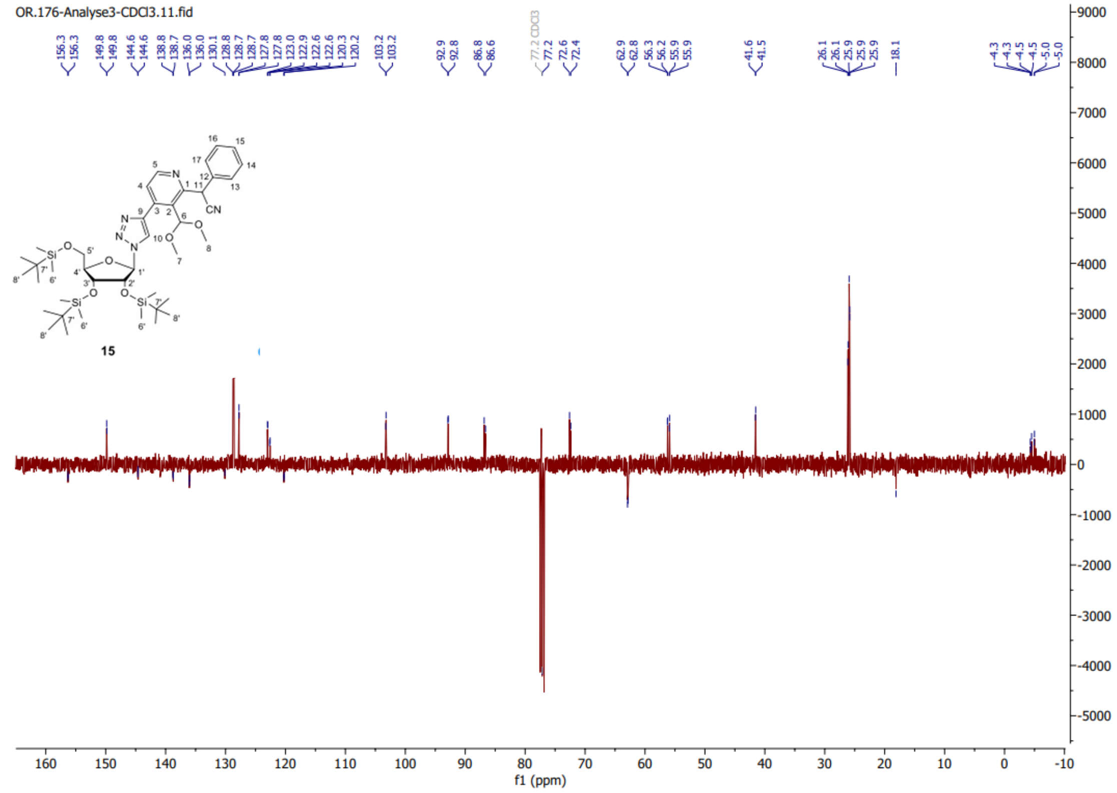

COSY spectrum of molecule **15** at 400 MHz

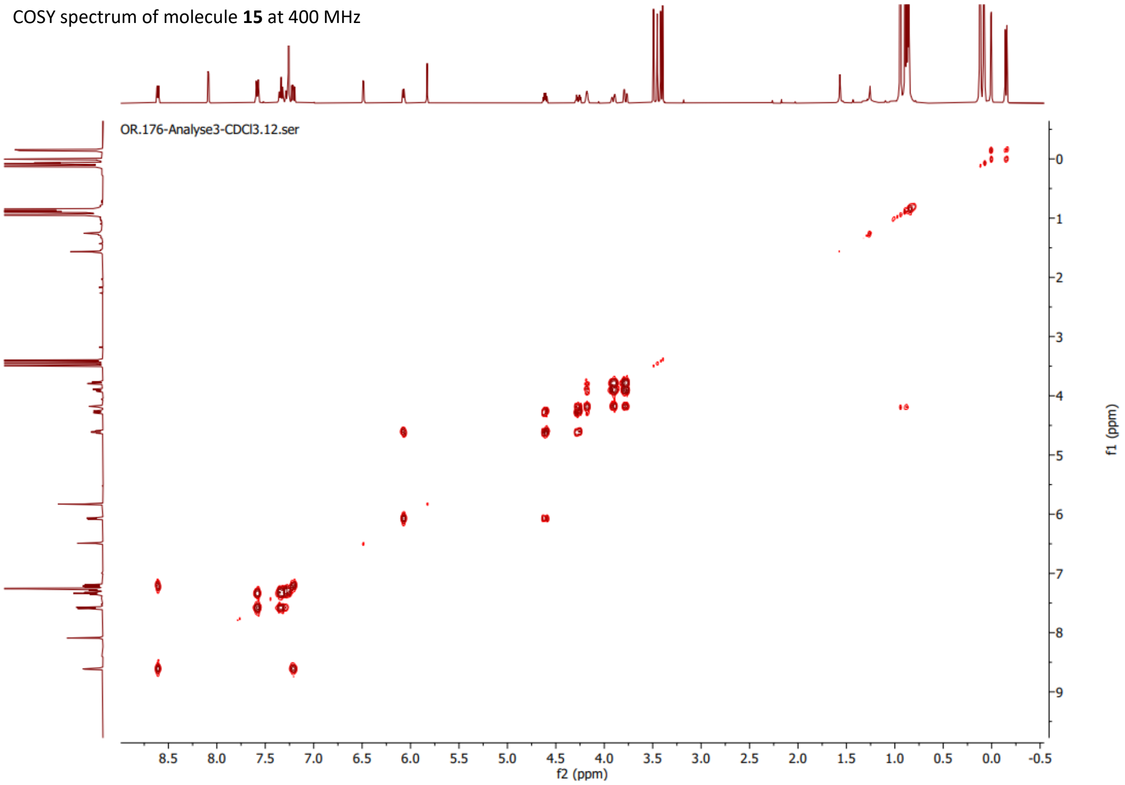

HSQC spectrum of molecule **15** at 400 MHz

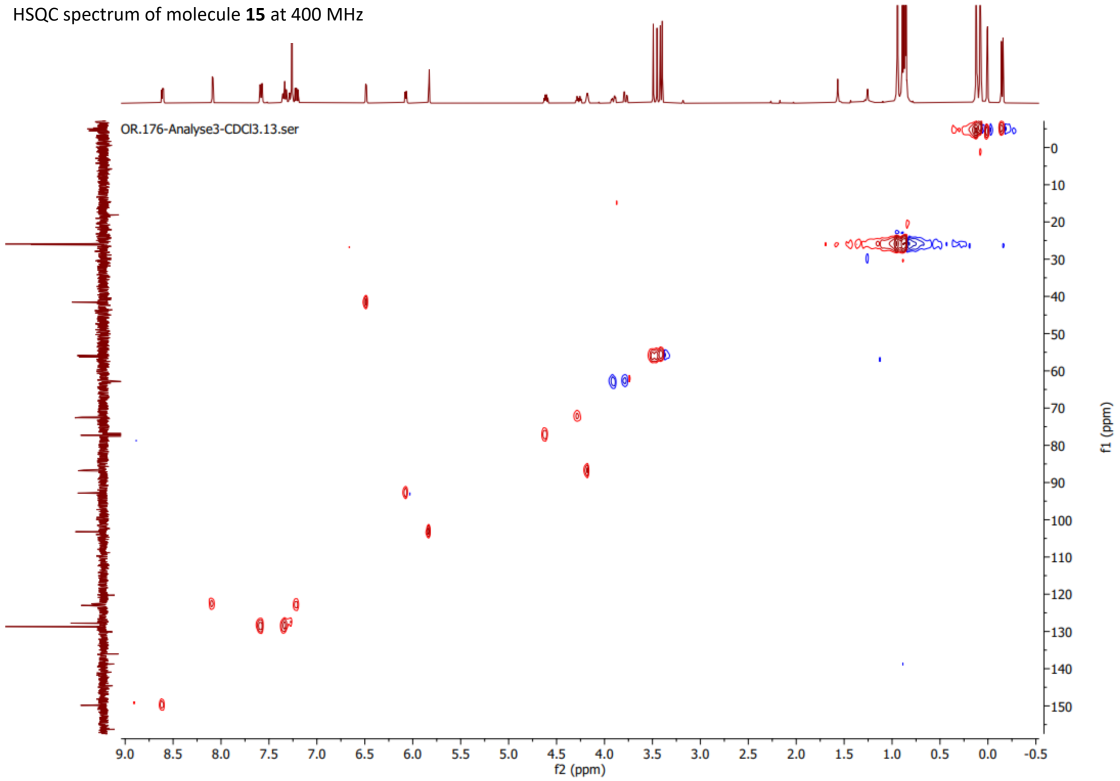

HMBC spectrum of molecule **15** at 400 MHz

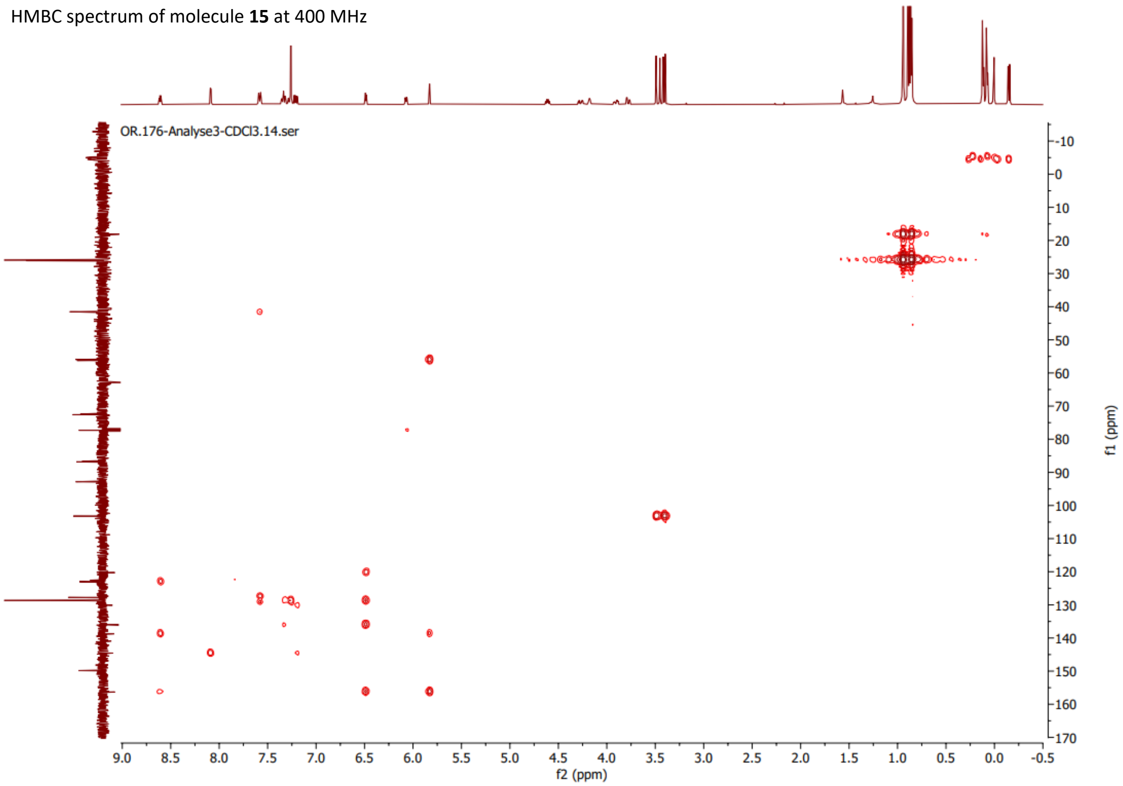

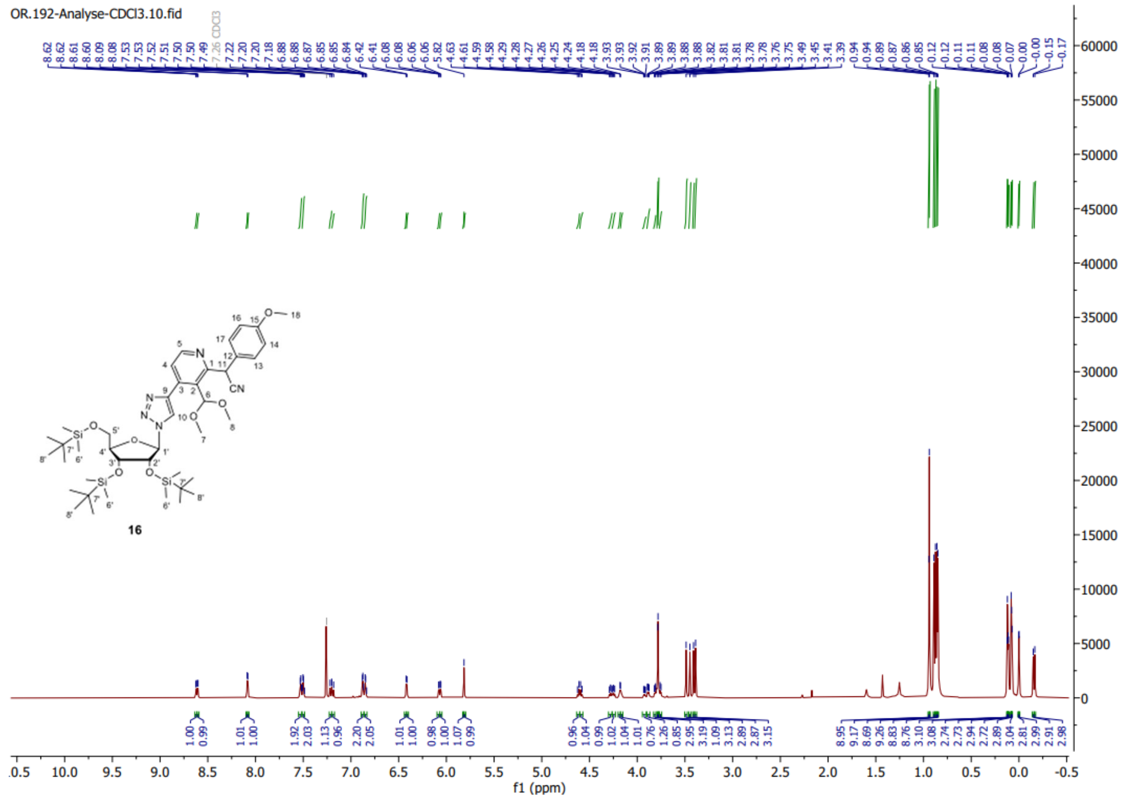

Zoom area 5 -9 ppm

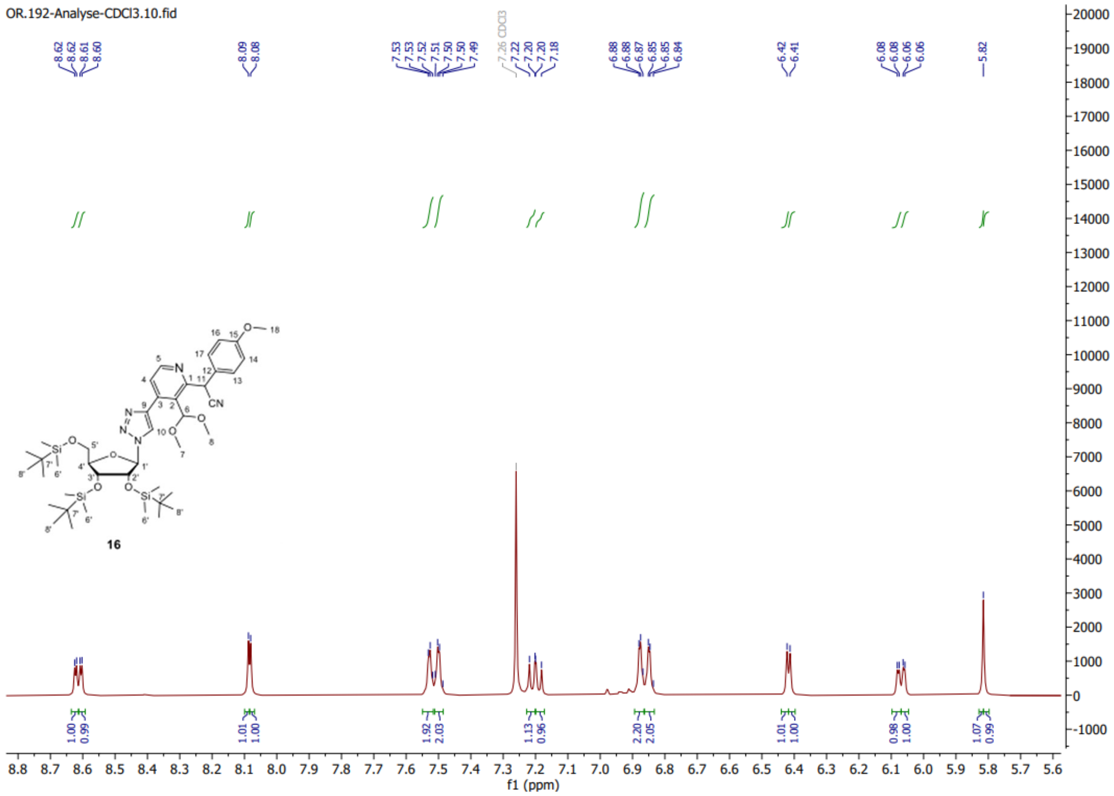

Zoom area 3 – 5 ppm

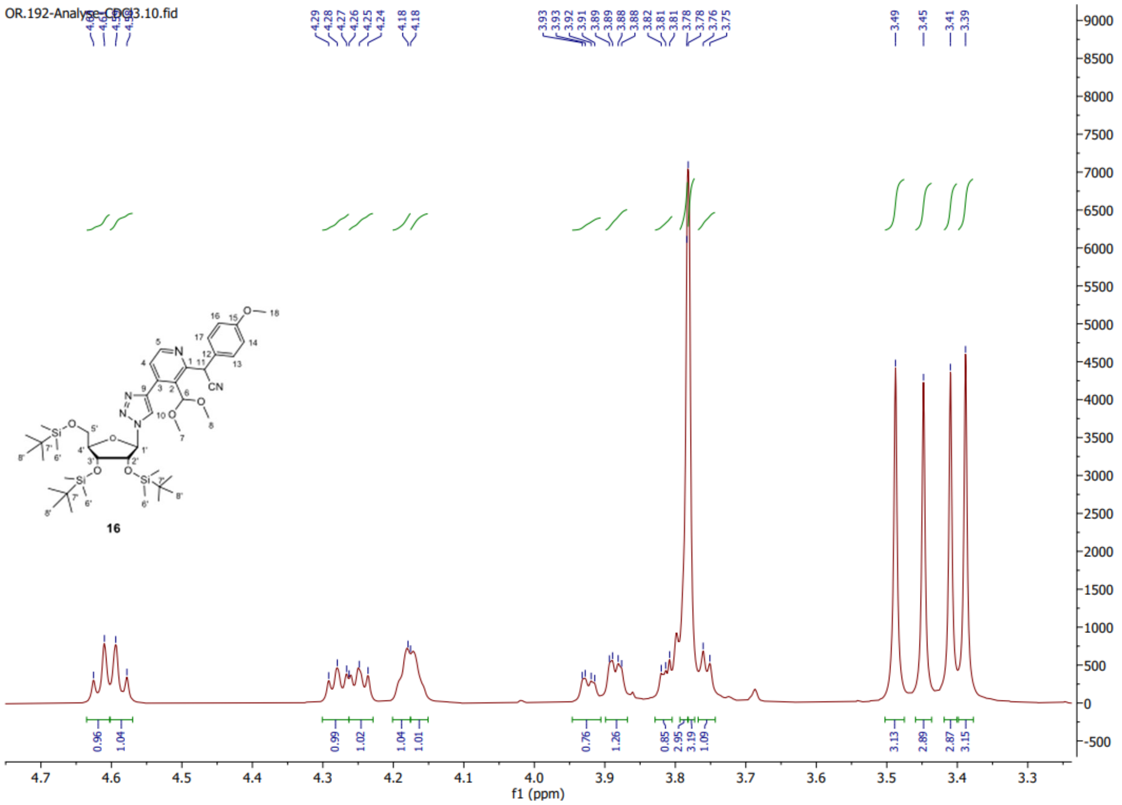
OR.192-Analyse-CDCl3.10.fid
16
f1 (ppm)

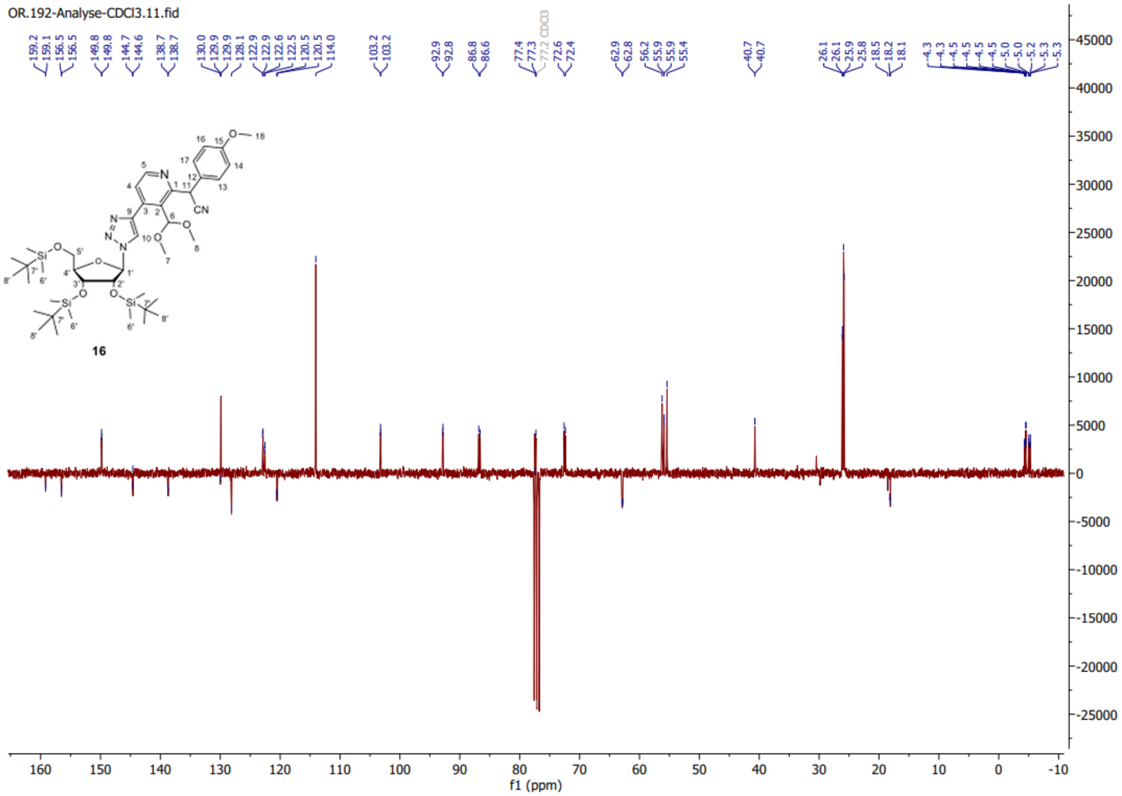
OR.192-Analyse-CDCl3.11.fid
16
f1 (ppm)

COSY spectrum of molecule **16** at 400 MHz

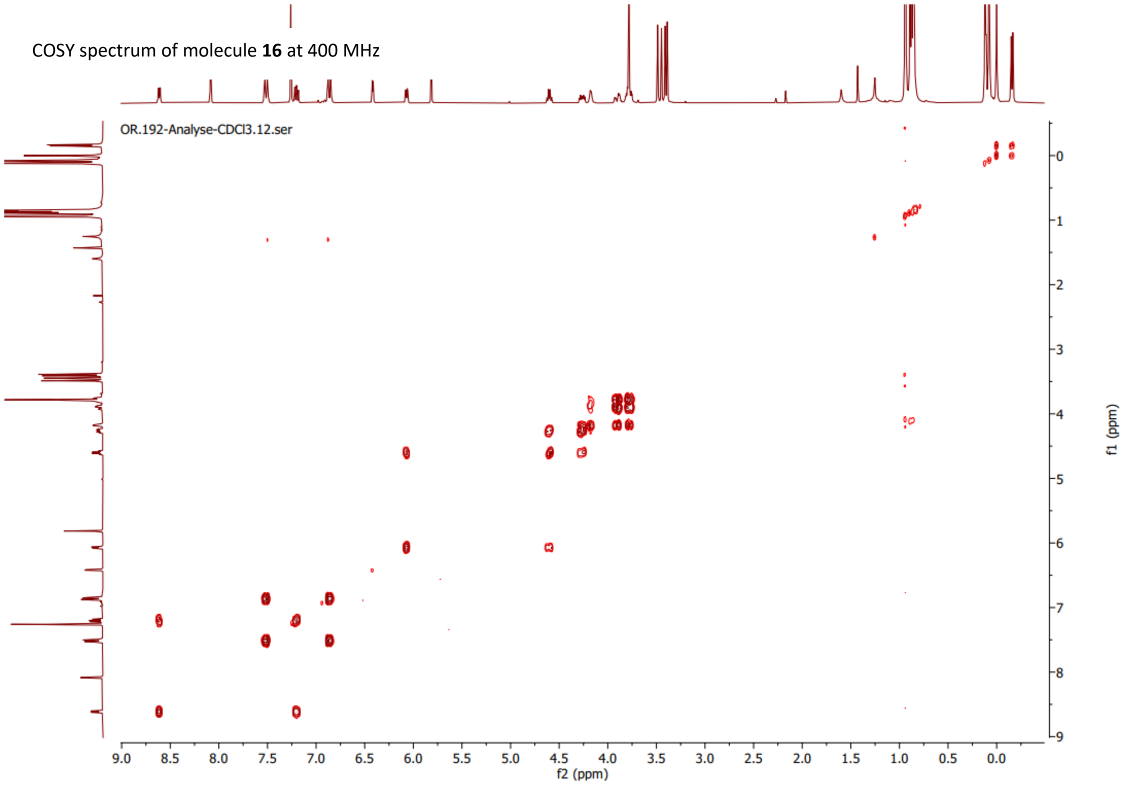

HSQC spectrum of molecule **16** at 400 MHz

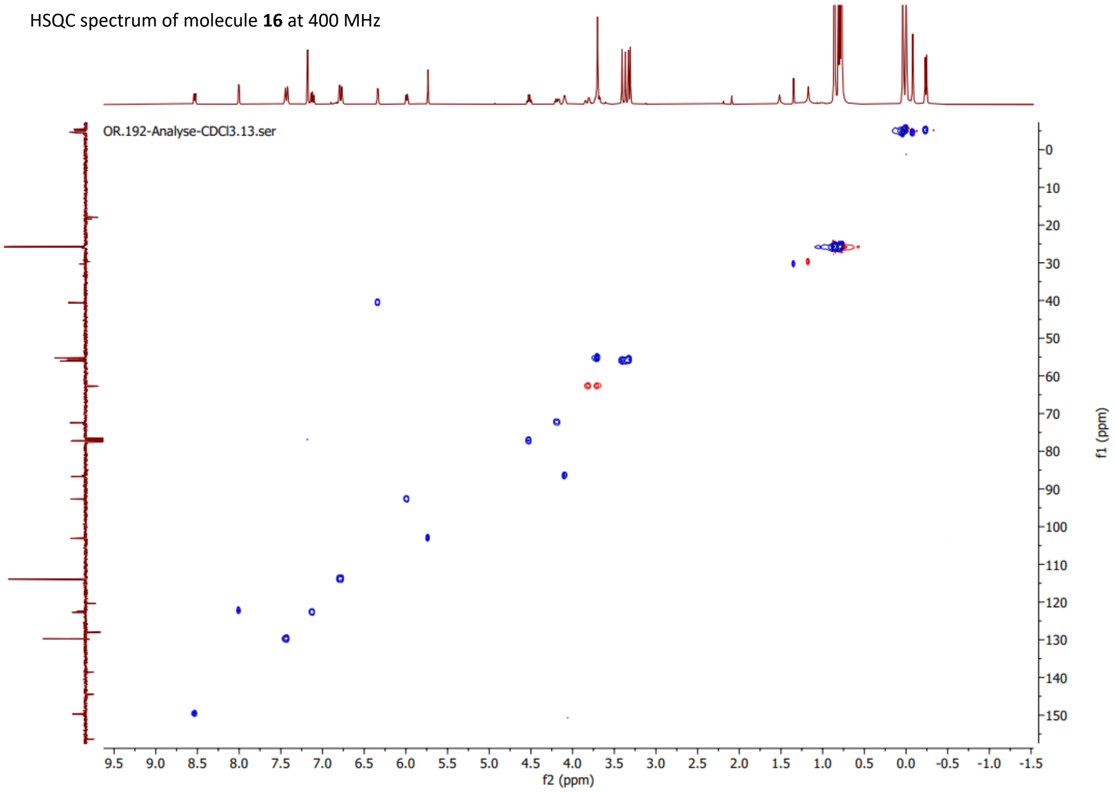

HMBC spectrum of molecule **16** at 400 MHz

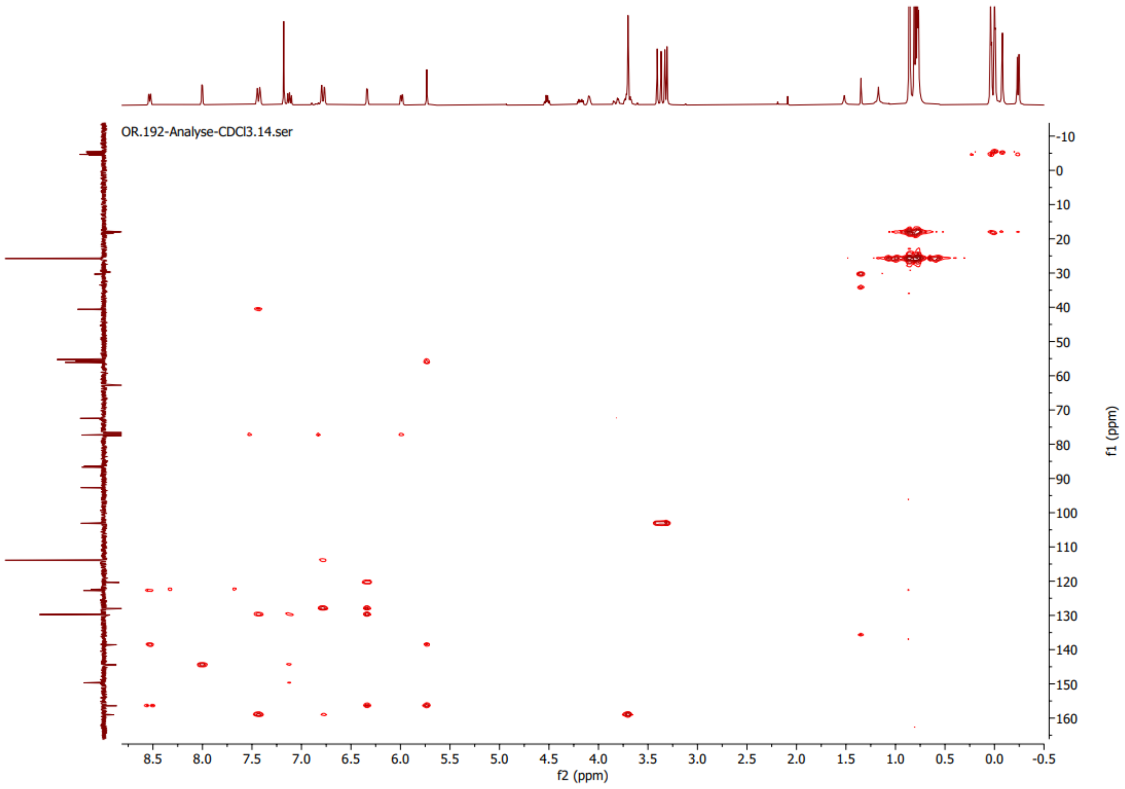

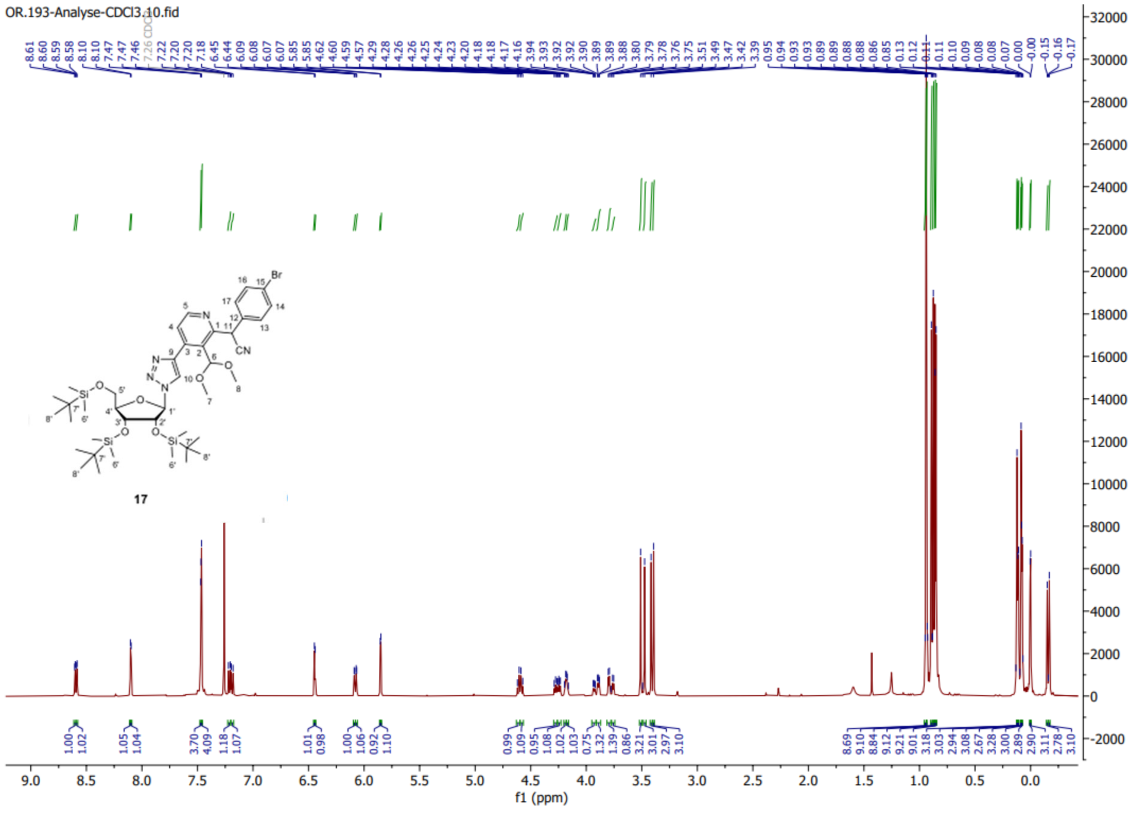

Zoom area 5 – 9 ppm

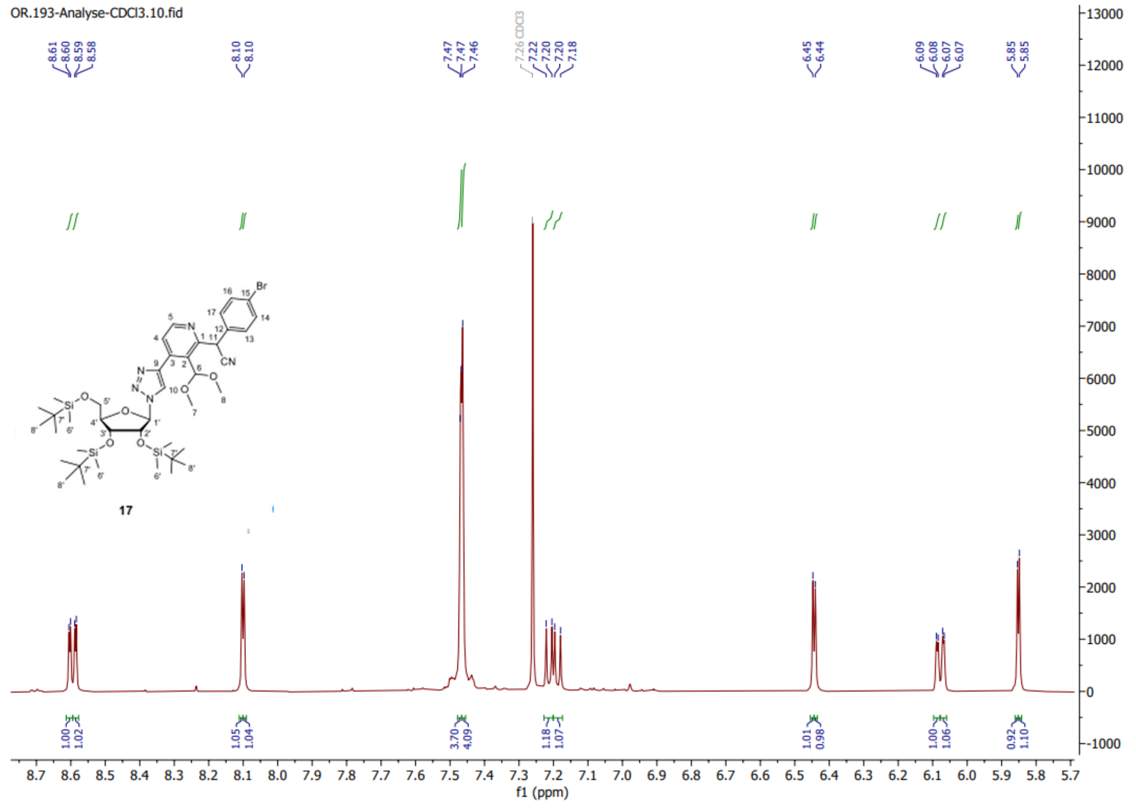

Zoom area 3 – 5 ppm

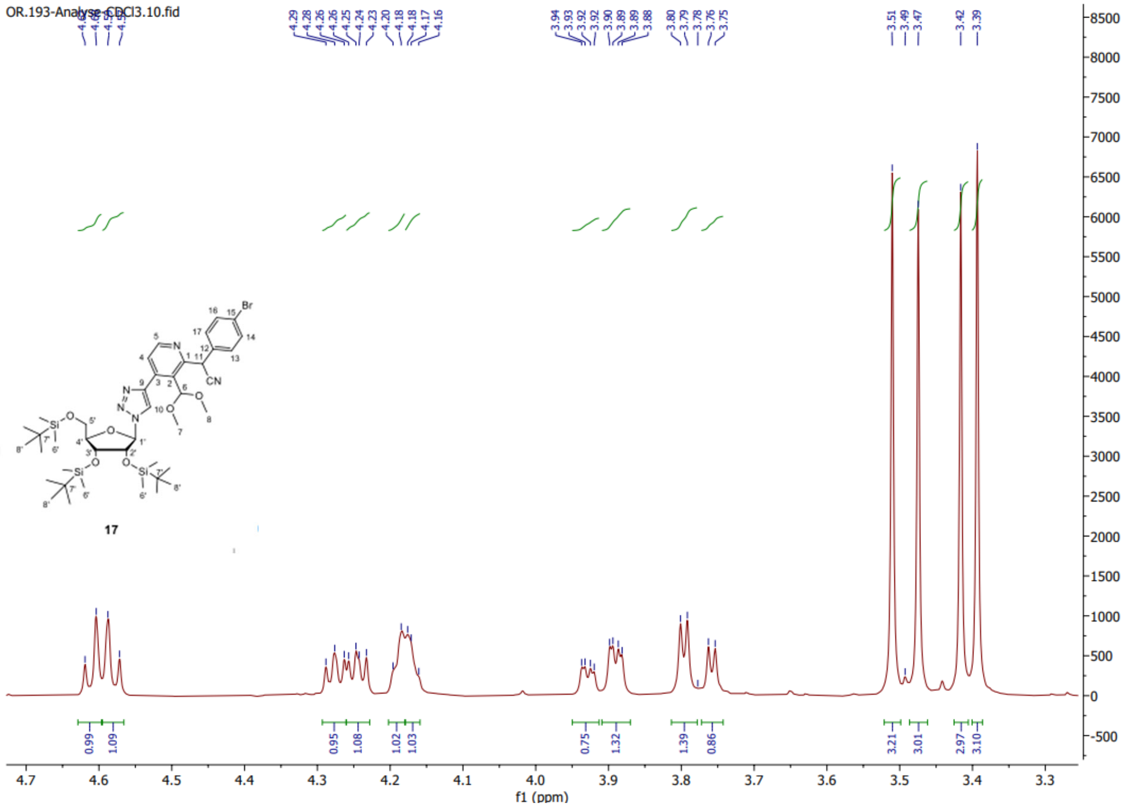

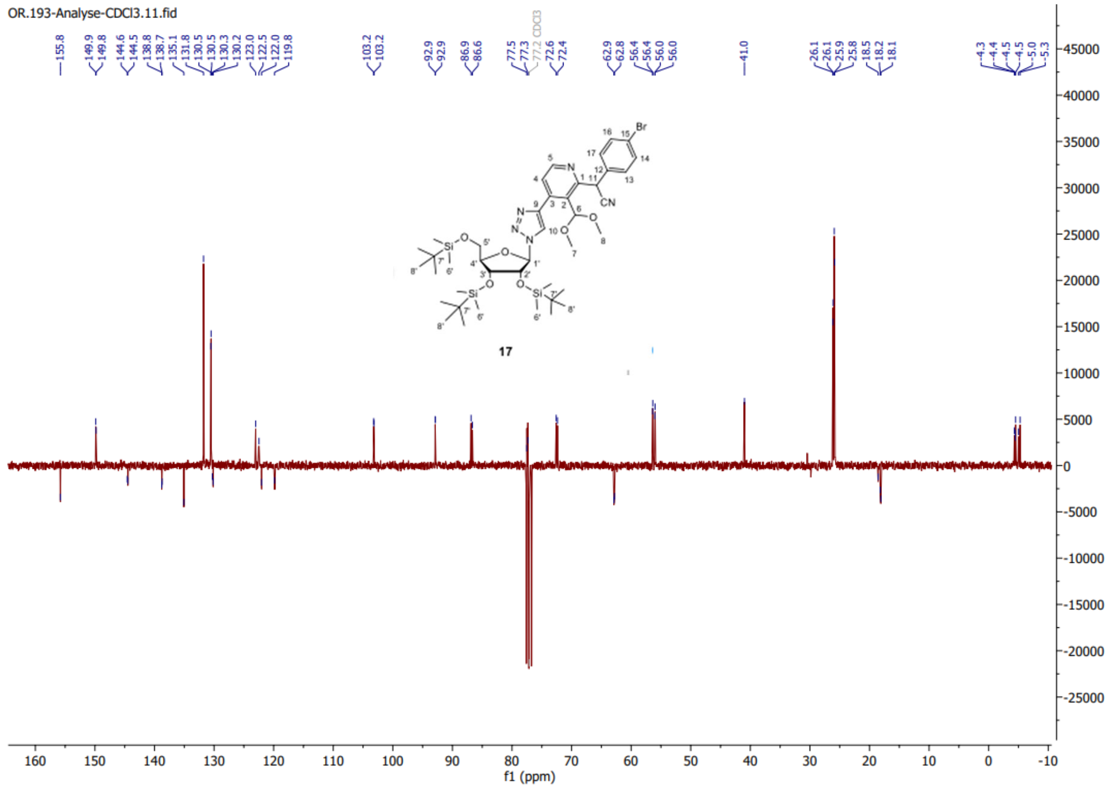

COSY spectrum of molecule **17** at 400 MHz

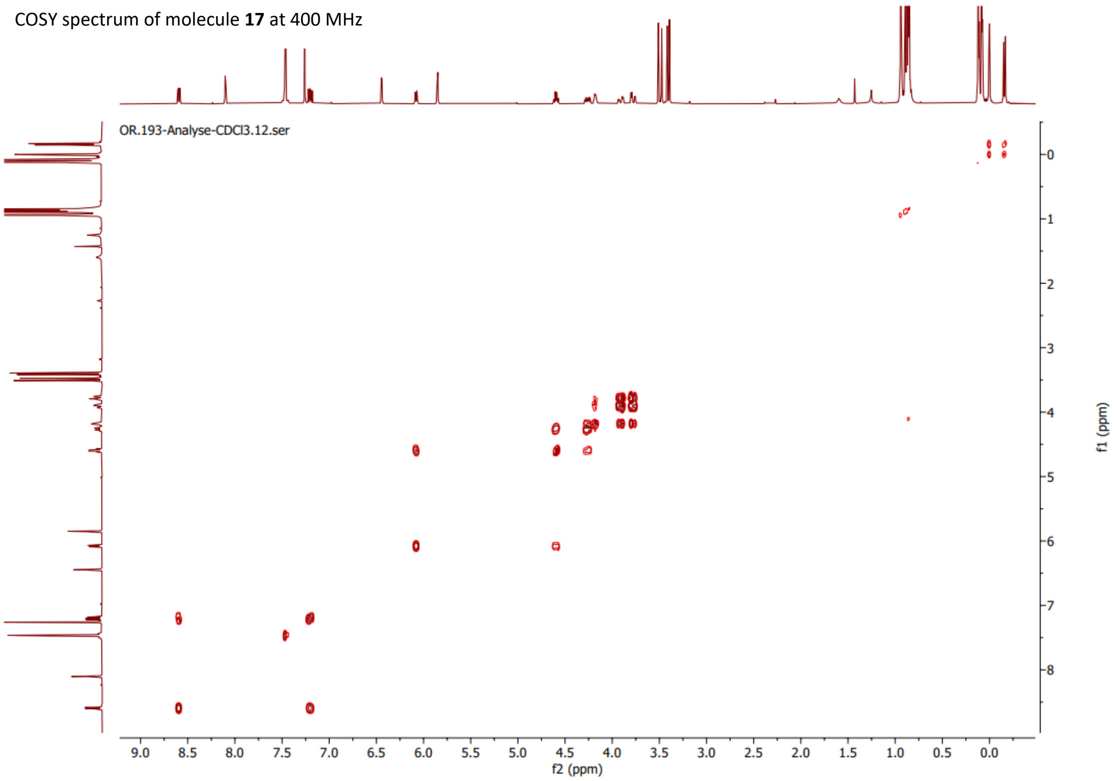

HSQC spectrum of molecule **17** at 400 MHz

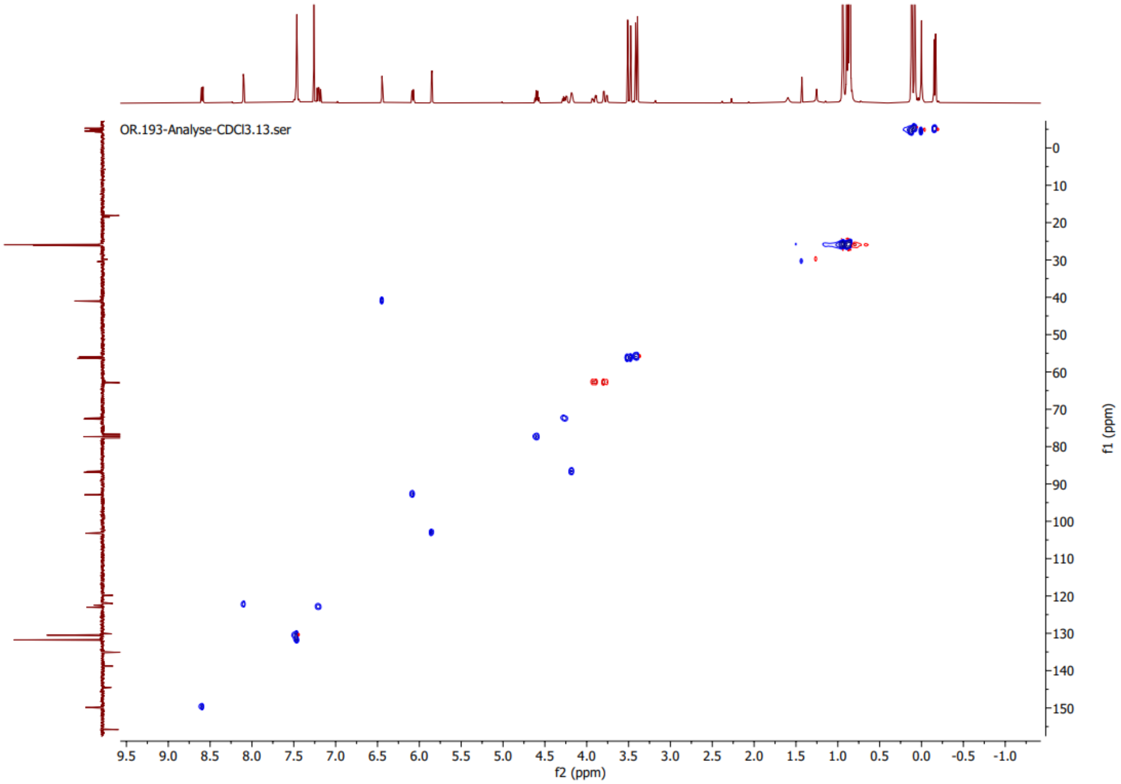

HMBC spectrum of molecule **17** at 400 MHz

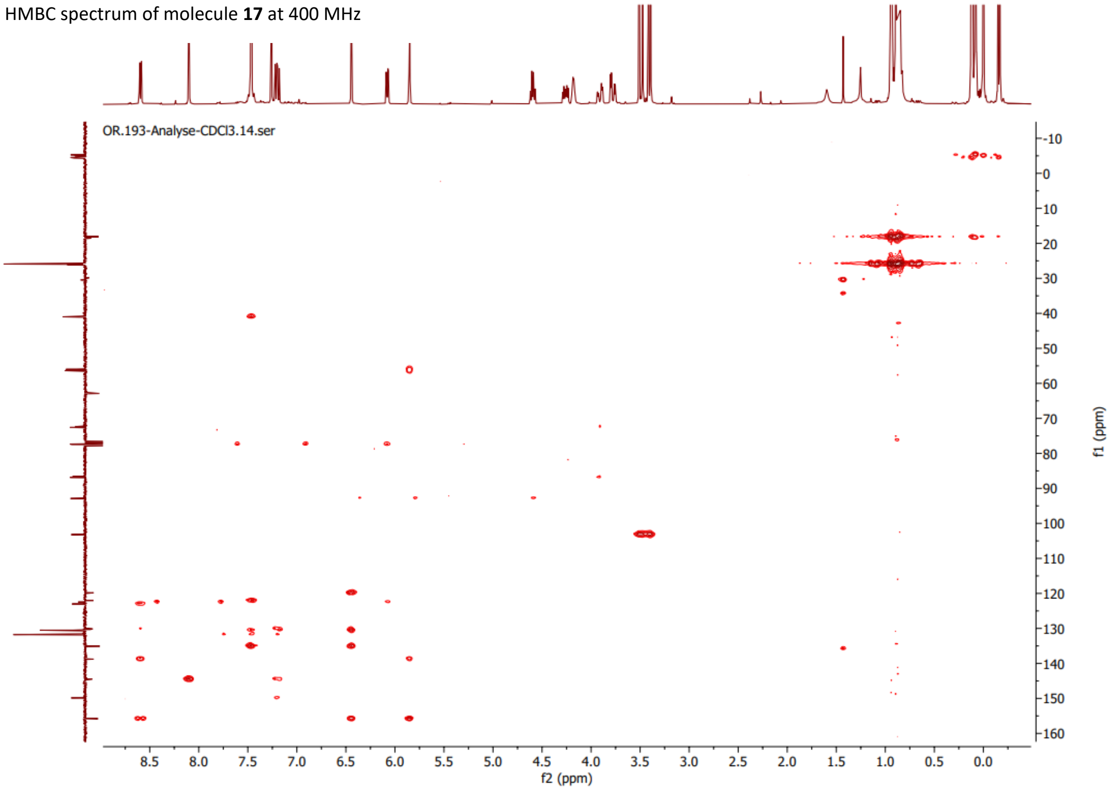

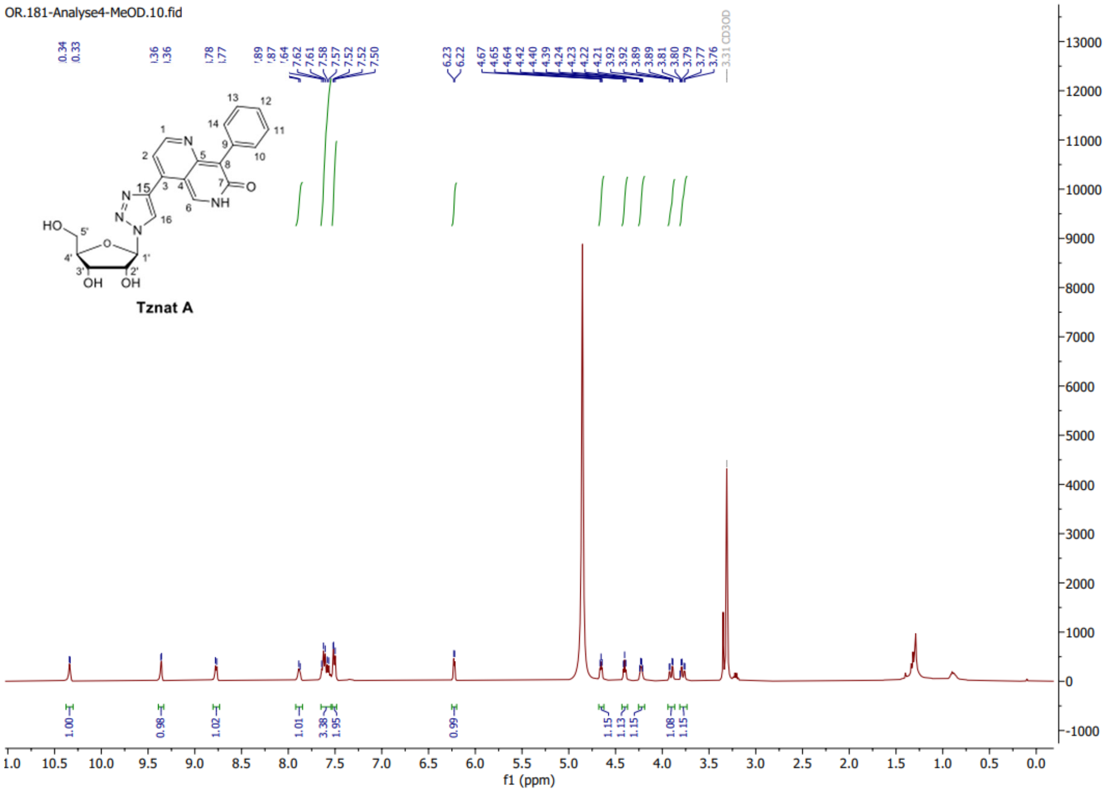
OR.181-Analyse4-MeOD.10.fid
Tznat A
f1 (ppm)

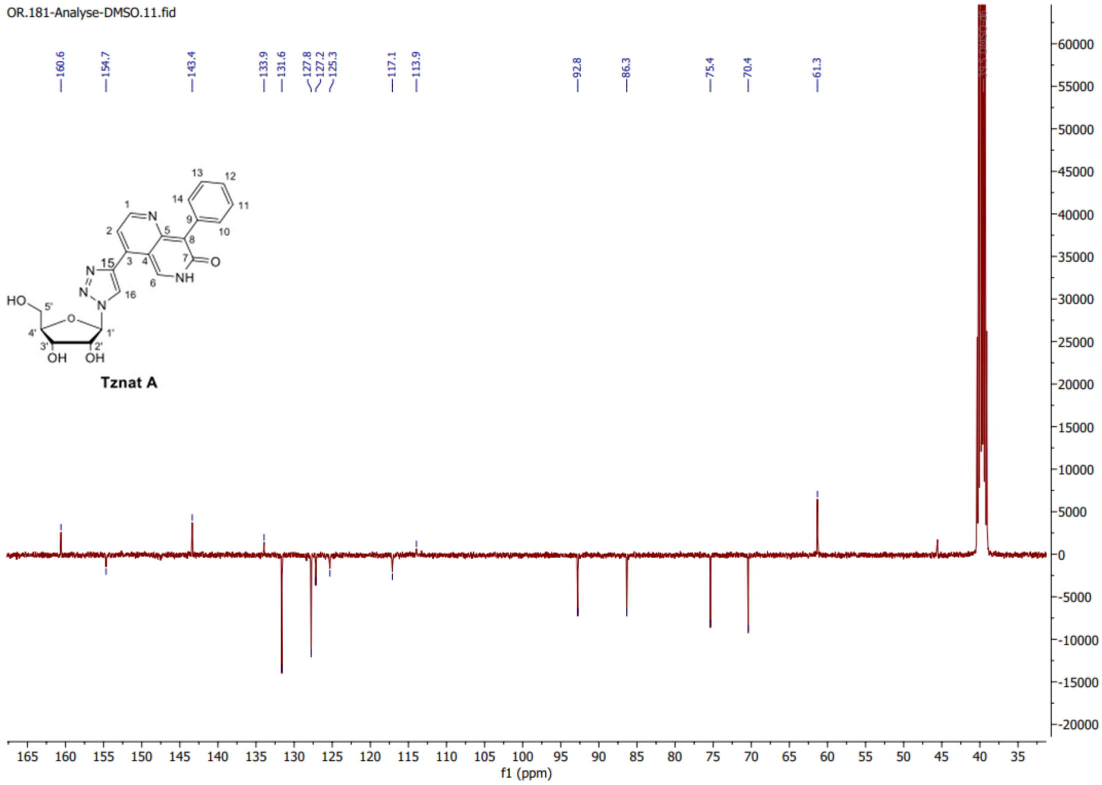
OR.181-Analyse-DMSO.11.fid
160.6
154.7
143.4
133.9
131.6
127.8
127.2
125.3
117.1
113.9
92.8
86.3
75.4
70.4
61.3
Tznat A
f1 (ppm)

COSY spectrum of **Tznat A** at 400 MHz

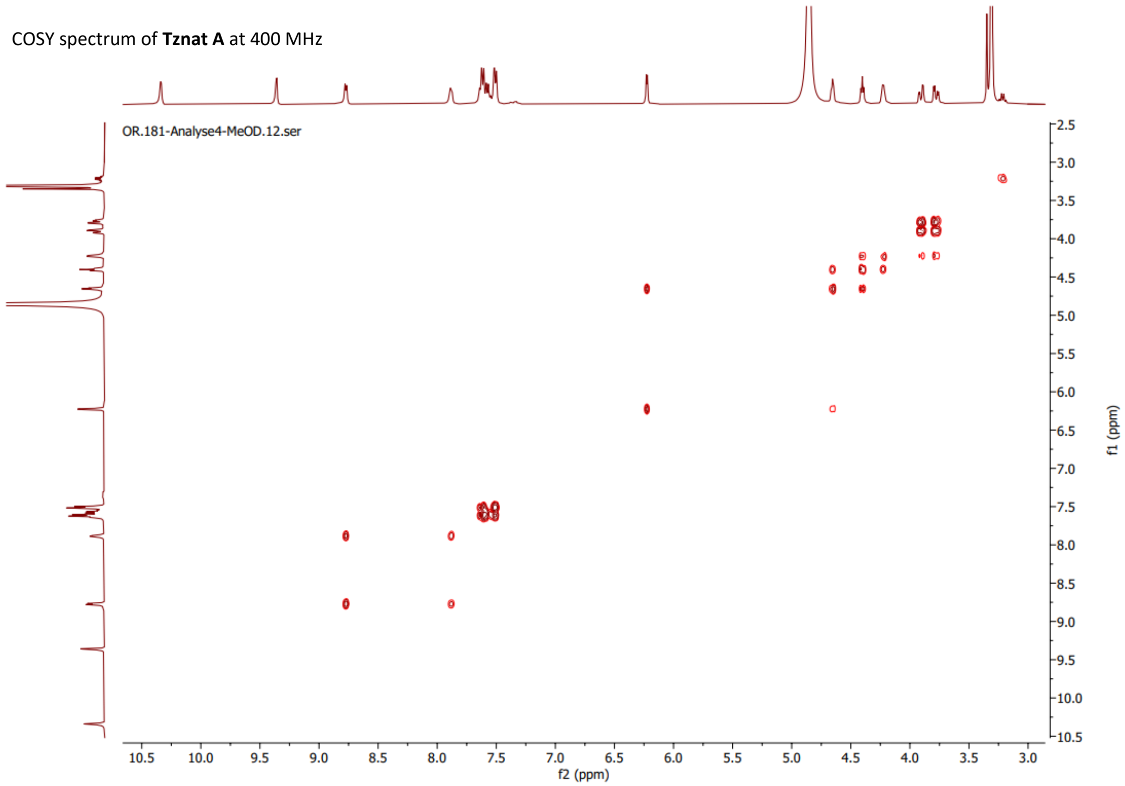

HSQC spectrum of **Tznat A** at 400 MHz

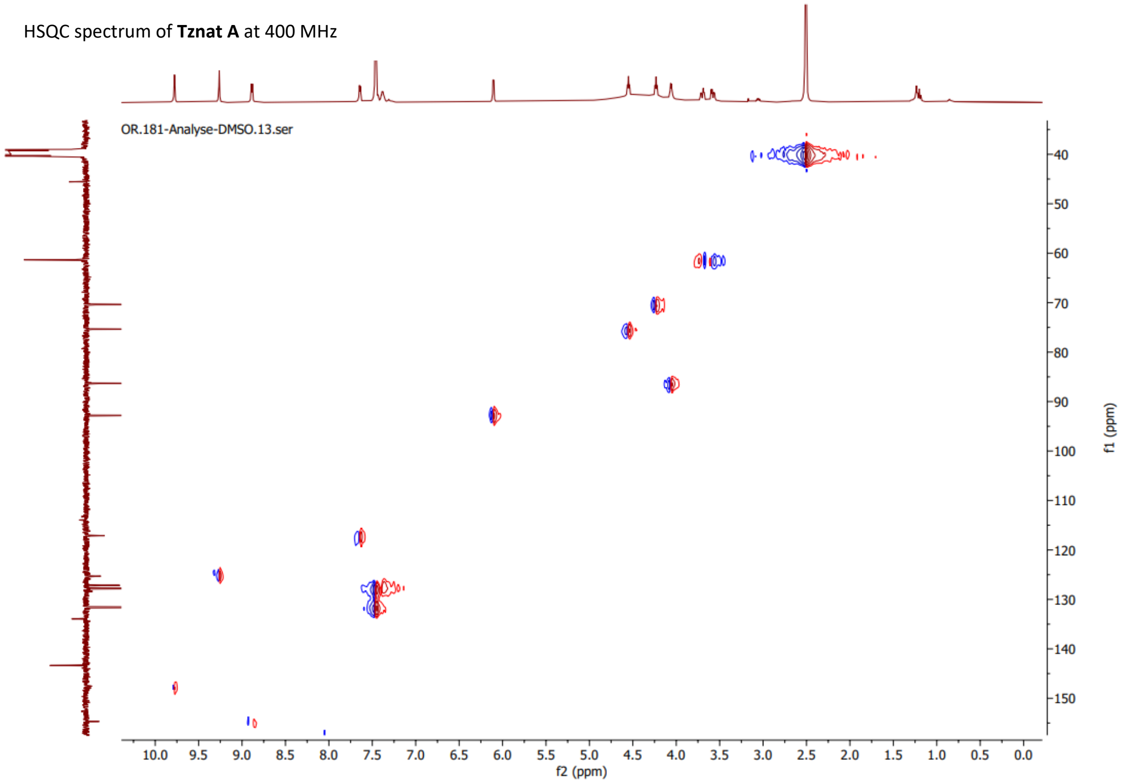

HMBC spectrum of **Tznat A** at 400 MHz

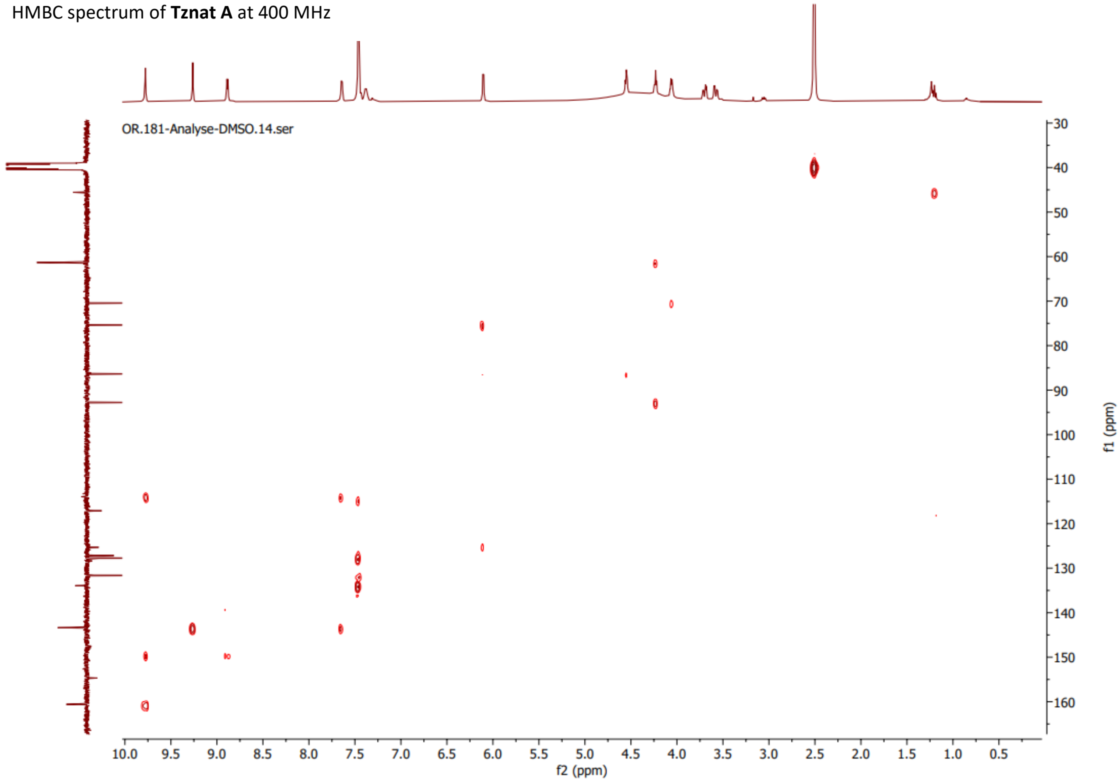

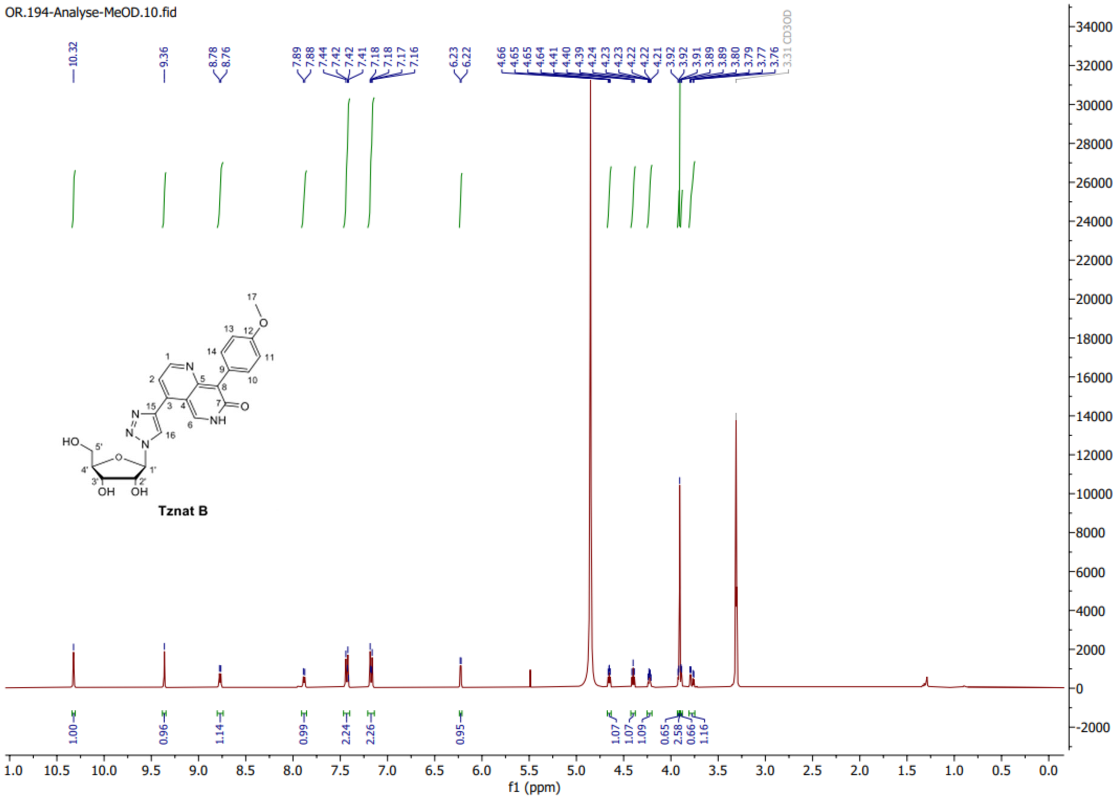
OR.194-Analyse-MeOD.10.fid
Tznat B
f1 (ppm)

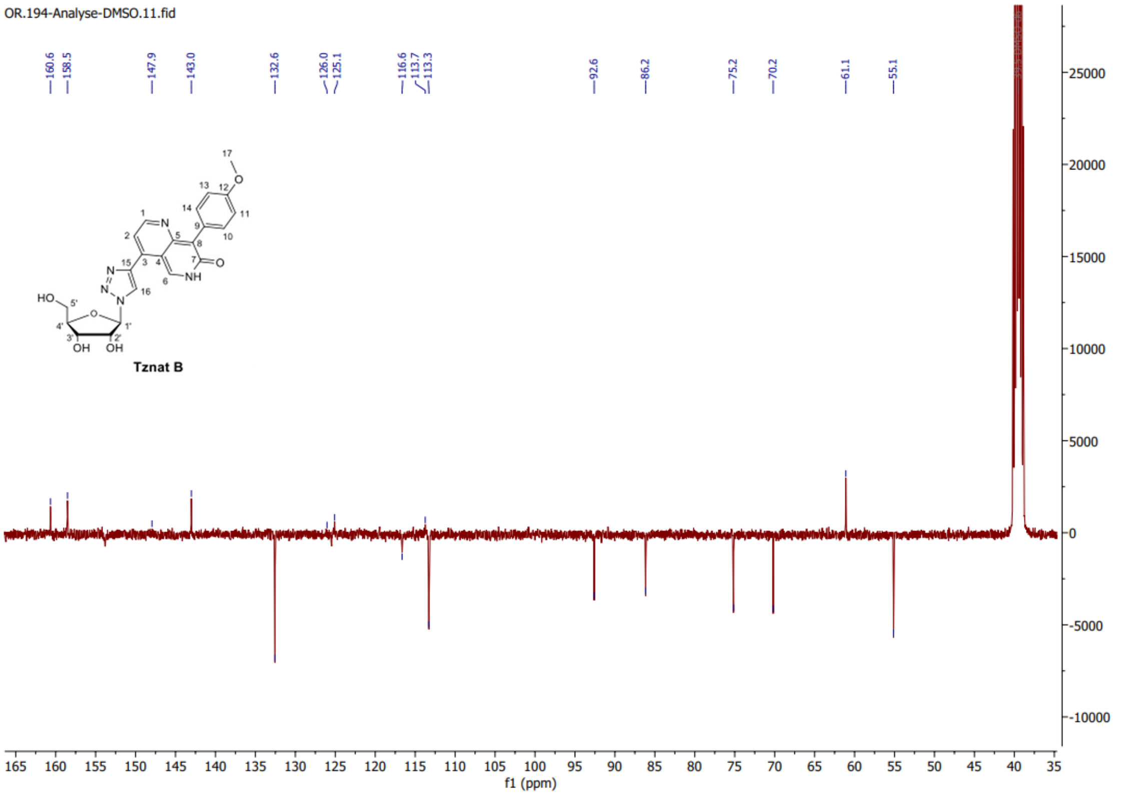
OR.194-Analyse-DMSO.11.fid
Tznat B
f1 (ppm)

COSY spectrum of **Tznat B** at 400 MHz

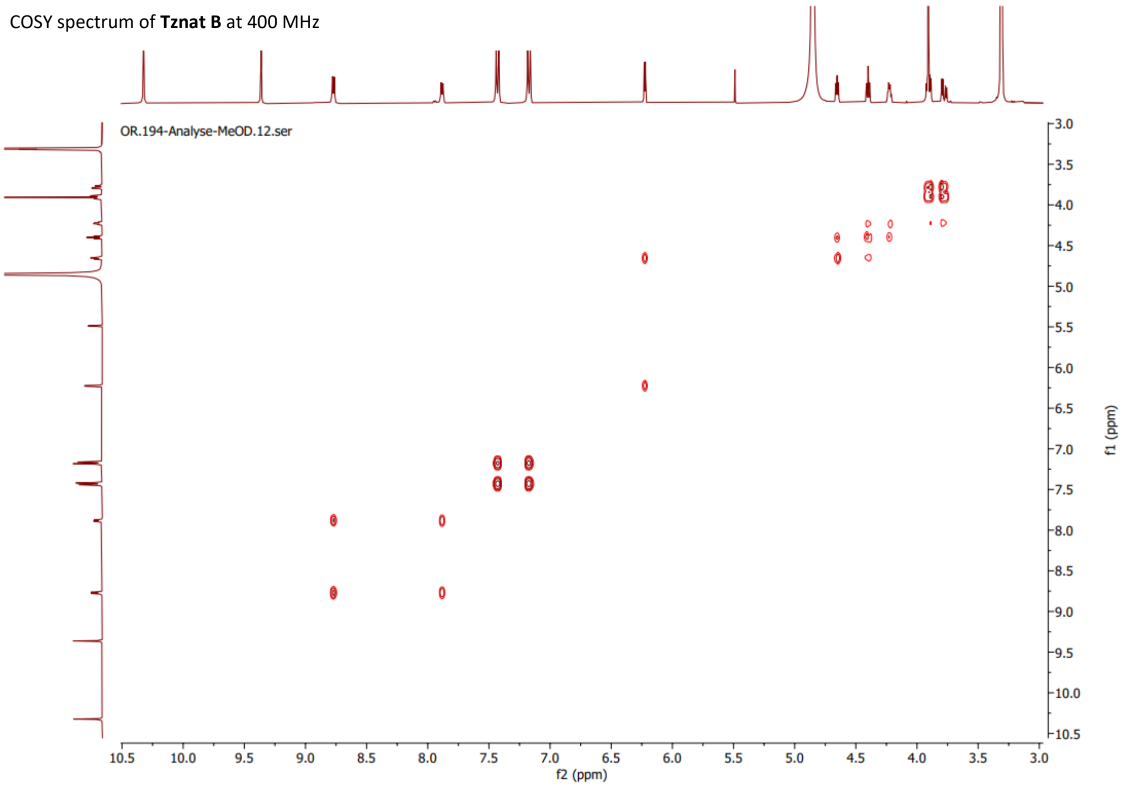

HSQC spectrum of **Tznat B** at 400 MHz

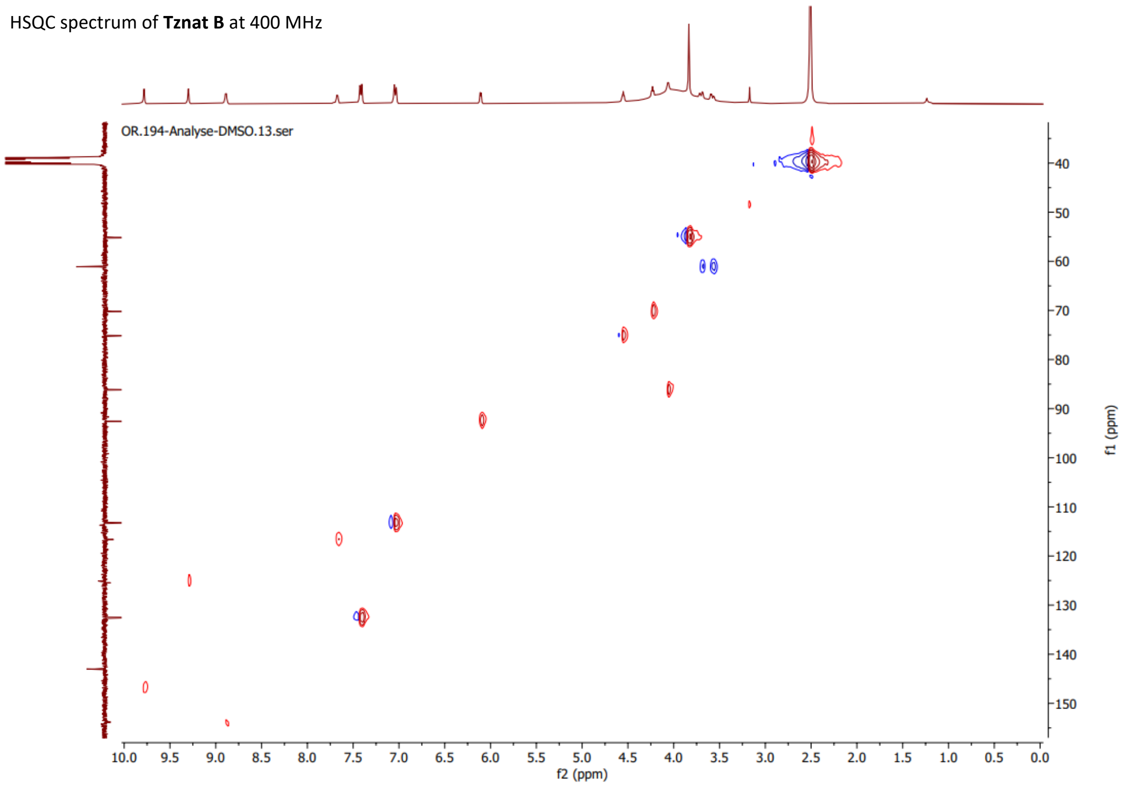

HMBC spectrum of **Tznat B** at 400 MHz

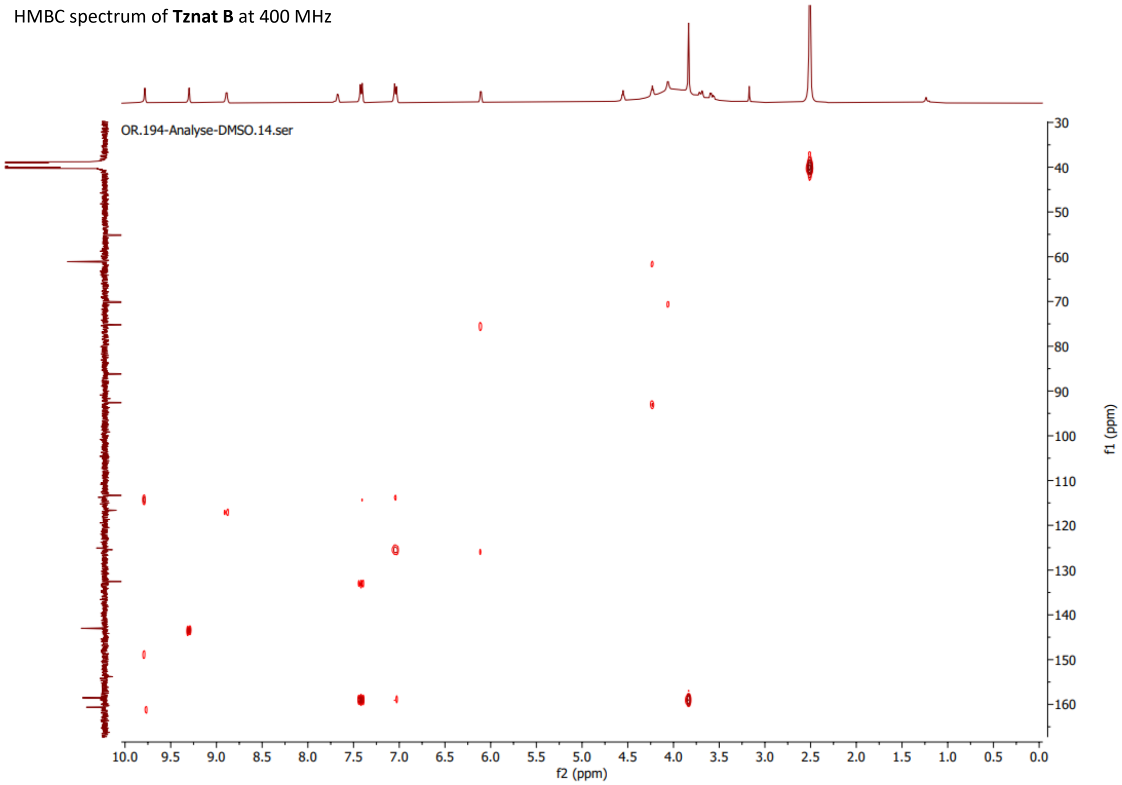

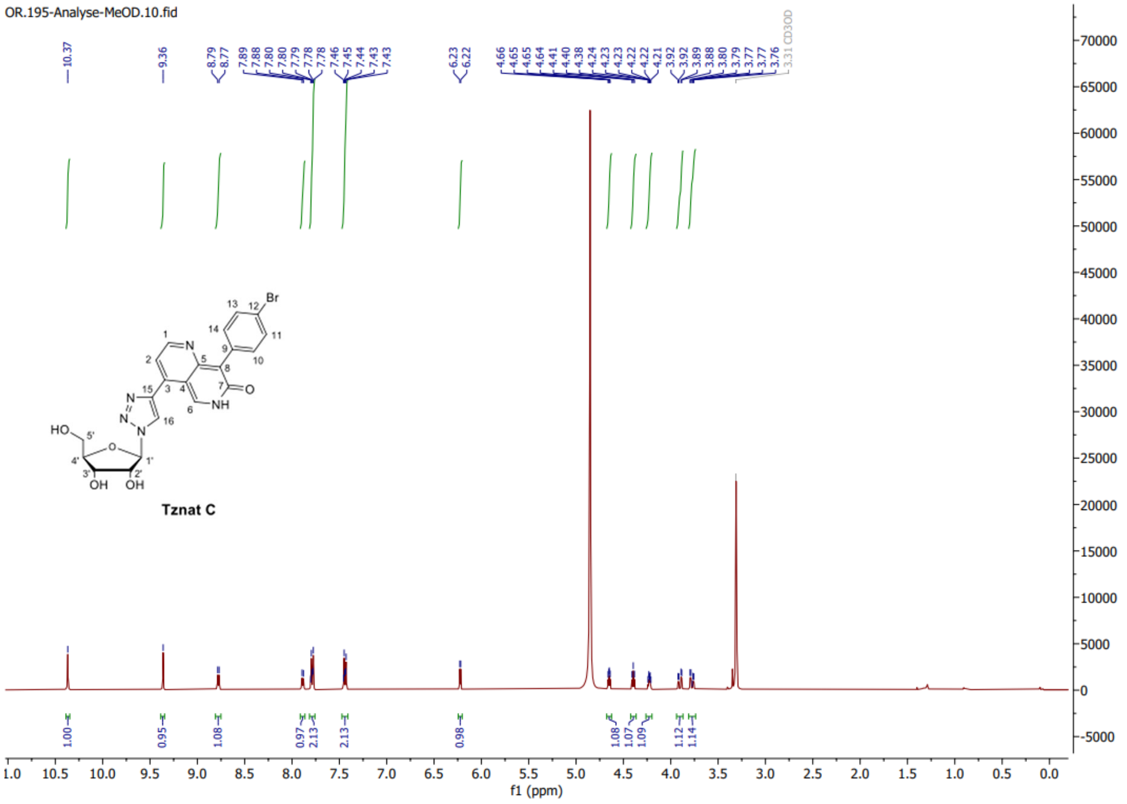

OR.195-Analyse-MeOD.10.fid
Tznat C
f1 (ppm)

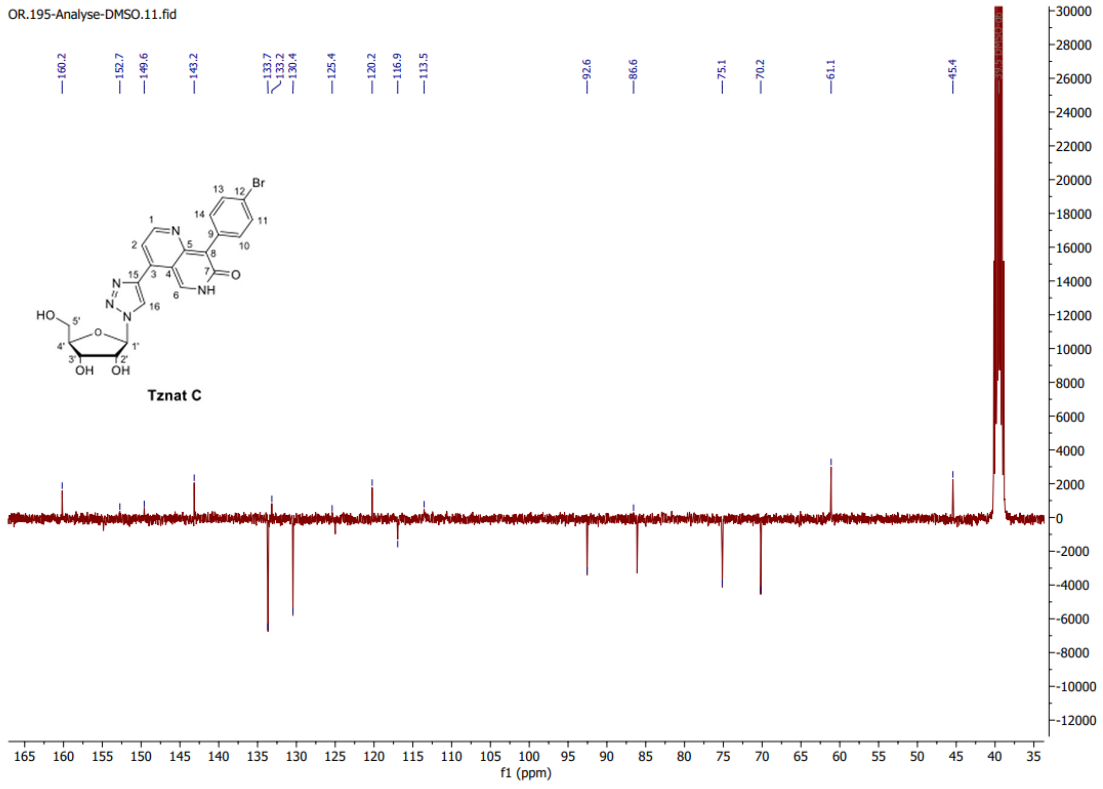

OR.195-Analyse-DMSO.11.fid
Tznat C
f1 (ppm)

COSY spectrum of **Tznat C** at 400 MHz

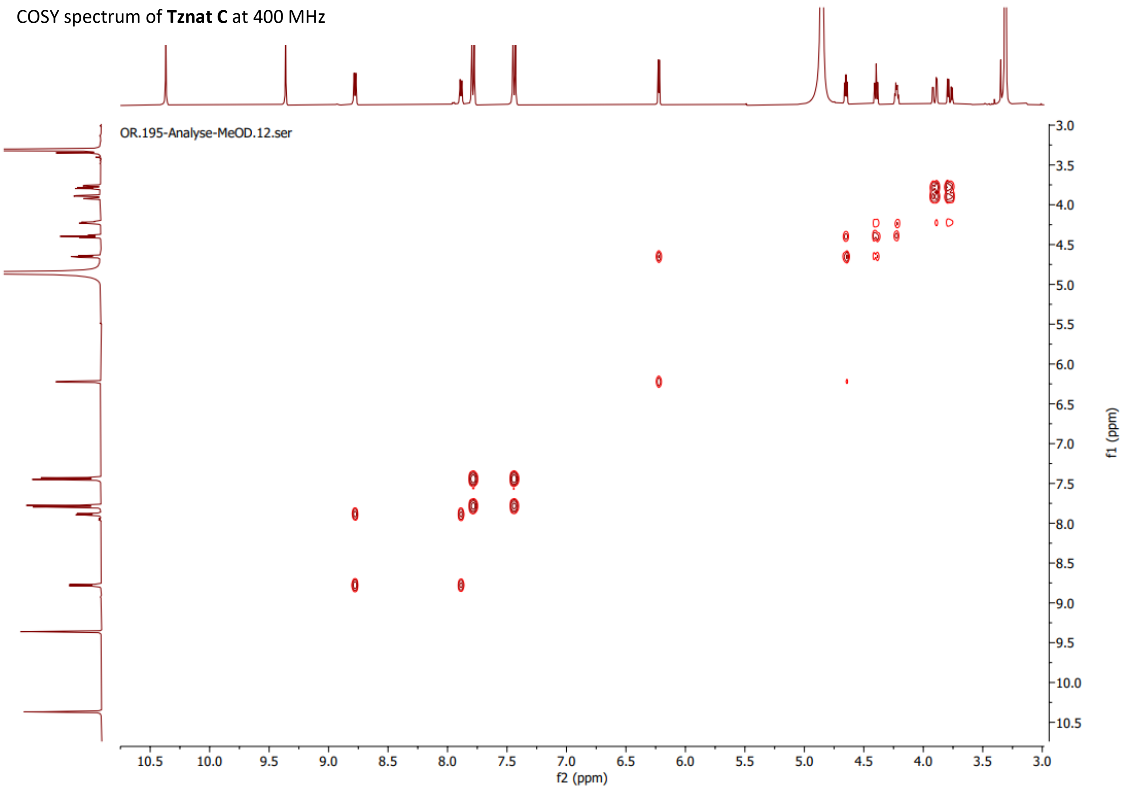

HSQC spectrum of **Tznat C** at 400 MHz

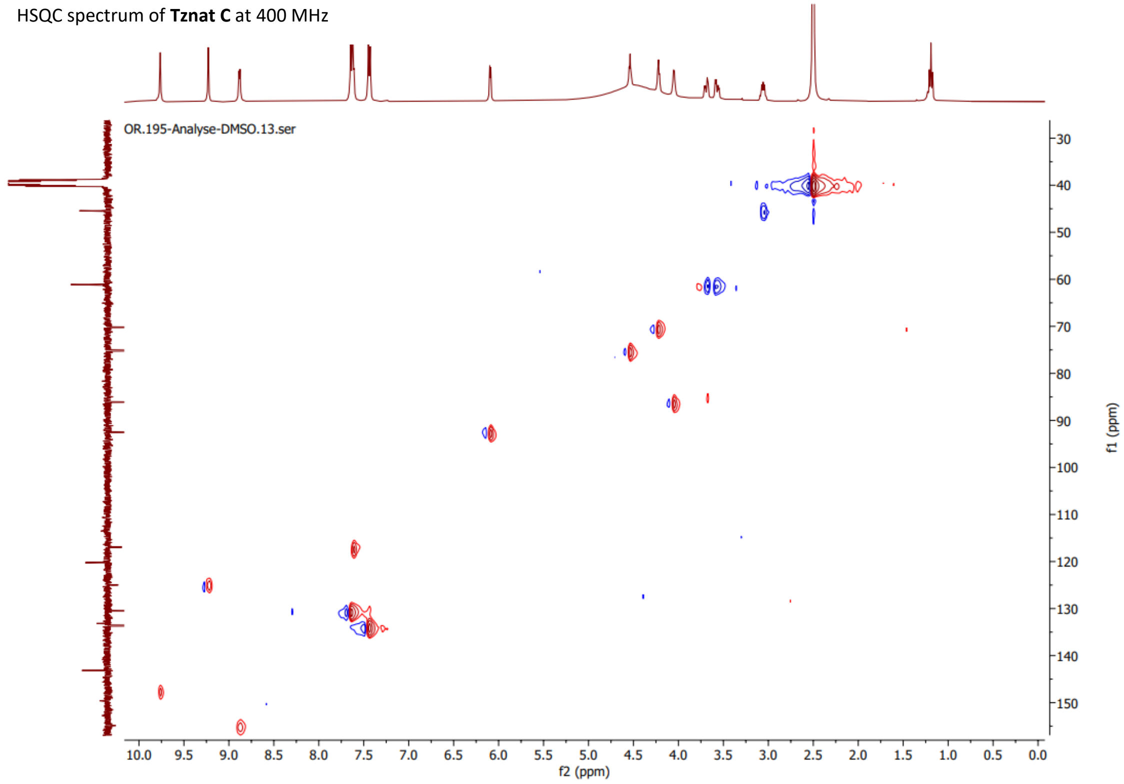

HMBC spectrum of **Tznat C** at 400 MHz

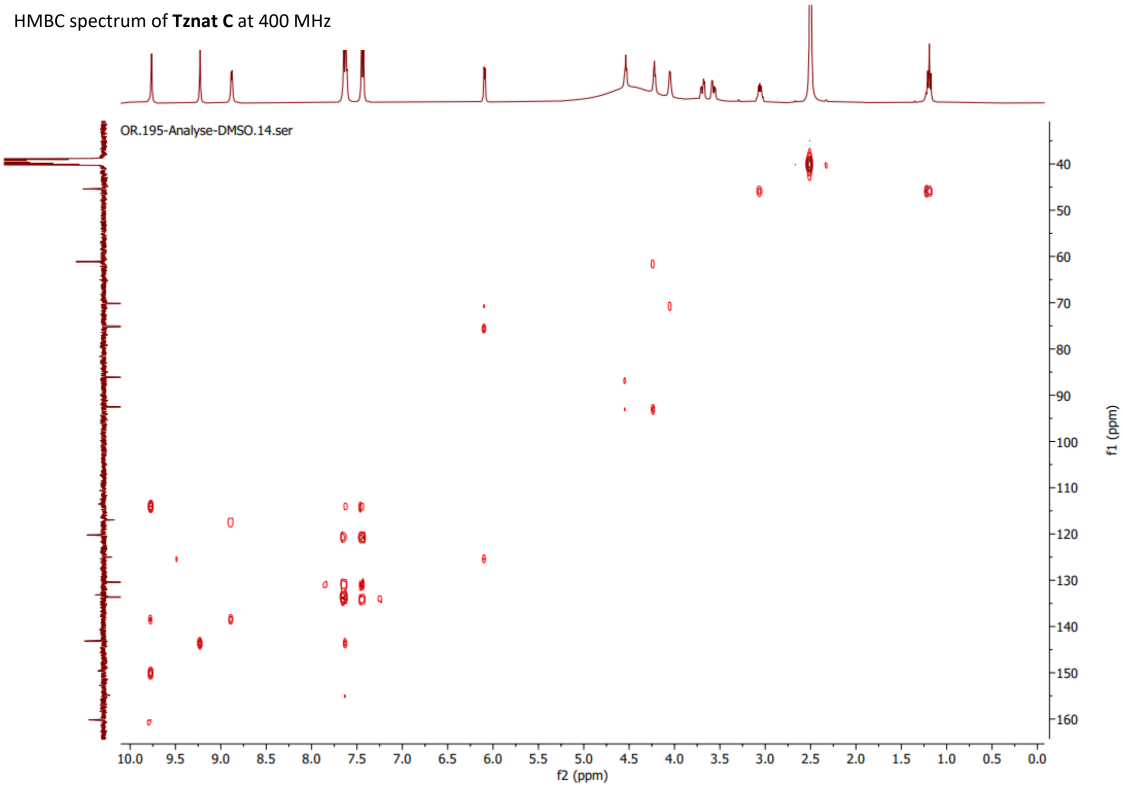

Supplement: Supplementary file 1 [file molecules-29-00687-s001.zip › molecules-2789808-supplementary.pdf]
